# Four new lanostane triterpenoids featuring extended *π*-conjugated systems from the stems of *Kadsura coccinea*

**DOI:** 10.1007/s13659-023-00376-1

**Published:** 2023-04-06

**Authors:** Qi-Qi Zhang, Kun Hu, Han-Dong Sun, Pema-Tenzin Puno

**Affiliations:** 1 State Key Laboratory of Phytochemistry and Plant Resources in West China, Yunnan Key Laboratory of Natural Medicinal Chemistry, Kunming Institute of Botany, Chinese Academy of Sciences, Kunming, 650201 Yunnan People’s Republic of China; 2 University of Chinese Academy of Sciences, Beijing, 100049 People’s Republic of China

**Keywords:** *Kadsura coccinea*, Lanostane triterpenoids, Extended π-conjugated systems, Quantum chemical calculation

## Abstract

Four new 14(13 → 12)-abeolanostane triterpenoids featuring extended *π*-conjugated systems, kadcoccitanes E–H (**1**–**4**), were obtained from the stems of *Kadsura coccinea* through using a HPLC − UV-guided approach. Their structural and configurational determination was accomplished through extensive spectroscopic analysis coupled with quantum chemical calculations. Kadcoccitanes E–H were tested for their cytotoxic activities against five human tumor cell lines (HL-60, A-549, SMMC-7721, MDA-MB-231, SW-480) but none of them exhibited activities at the concentration 40 μM.

**Graphical Abstract:**

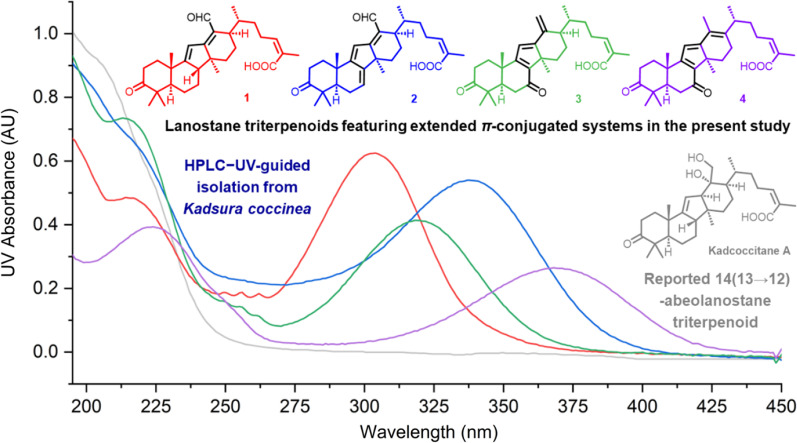

**Supplementary Information:**

The online version contains supplementary material available at 10.1007/s13659-023-00376-1.

## Introduction

The plant family Schisandraceae is composed of two genera, *Schisandra* and *Kadsura*. Plant species within this family have attracted much attention for serving as an eminent source of triterpenoids with diverse structures and various bioactivities [1, 2]. Triterpenoids from the Schisandraceae family can be categorized into three major groups on the basis of their different carbon frameworks: lanostanes, cycloartanes, and schinortriterpenoids [1, 2]. Their fascinating architectures have aroused great interest among organic synthetic chemists and a few molecules have been successfully totally synthesized [3].

*Kadsura coccinea* is a vine plant grown widely in southern China. It possesses reputed medicinal value and has been utilized as effective remedies for gastropathy, rheumatic arthritis, etc. In the last decades, considerable efforts in phytochemical research on *K. coccinea* have led to the characterization of many lanostane triterpenoids and more than 10 kinds of novel scaffolds [4–9], including the recently reported neokadcoccitane A [10] possessing an aromatic ring D and an extended *π*-conjugated systems (EPCS). The EPCS moieties are not commonly present in lanostane triterpenoids from the Schisandraceae family, but can be found in a few cycoartane triterpenoids. For example, kadlongilactones A and B were both found to possess an *α*,*β*,*γ*,*δ*-unsaturated lactone moiety and characterized as potent inhibitors of K562 cells [11]. To explore more structurally and biologically fascinating triterpenoids from *K. coccinea*, further phytochemical investigations were carried out on stems collected in Jingzhou County, Hunan Province. During this research, several unusual chromatographic peaks in HPLC with maximum ultraviolet absorption (UV) wavelengths at 300 ~ 380 nm were observed, suggesting the existence of triterpenoids with EPCS. Subsequent UV-guided isolation afforded kadcoccitanes E–H (**1**–**4**), four undescribed 14(13 → 12)-abeolanostane triterpenoids featuring *α*,*β*,*γ*,*δ*-unsaturated aldehyde or *α*,*β*,*γ*,*δ*,ε,ζ-unsaturated aldehyde/ketone. Herein, the isolation and structure elucidation of kadcoccitanes E–H (**1**–**4**), along with their cytotoxicity evaluation were described.

## Results and discussion

Kadcoccitane E (**1**), a light yellow amorphous solid, was assigned the molecular formula of C_30_H_42_O_4_ through its HRESIMS ([M − H]^–^ *m/z* 465.3014, calcd 465.3010), indicating ten degrees of unsaturation (DOUs). The ^1^H NMR spectrum (Table 1) exhibited resonances for two olefinic protons (*δ*_H_ 5.90, m; *δ*_H_ 6.86, d, *J* = 2.1 Hz), an aldehyde group (*δ*_H_ 10.33, s), a doublet methyl group (*δ*_H_ 1.10, d, *J* = 6.8 Hz), and five singlet methyl groups (*δ*_H_ 0.99, 1.05, 1.13, 1.18, and 2.07). The ^13^C NMR and DEPT spectra (Table 2) exhibited 30 carbon signals, including six methyls, eight methylenes, seven methines (two olefinic and one aldehyde), and nine nonprotonated carbons (one carboxyl, one carbonyl, four olefinic, and three quaternary carbons). Apart from the six DOUs occupied by carboxyl, carbonyl, aldehyde and olefinic groups, the remaining four ones uncovered that **1** possessed a tetracyclic architecture.

**Table 1:** ^1^H NMR data of compounds **1**–**4** (*δ* in ppm, *J* in Hz)

No	1^a^	2^a^	3^a^	4^b^
1*α*	1.86, overlap	1.87, overlap	1.82, overlap	1.83, m
1*β*	2.06, m	2.20, overlap	2.18, overlap	2.15, m
2*α*	2.48, ddd (15.6, 6.0, 3.1)	2.43, ddd (15.2, 4.9, 2.8)	2.57, m	2.56, ddd (15.9, 6.8, 3.3)
2*β*	2.71, ddd (15.6, 12.6, 6.0)	2.79, overlap	2.79, overlap	2.77, m
5	1.49, overlap	1.82, overlap	2.39, dd (13.8, 3.4)	2.41, dd (13.9, 3.3)
6*α*	1.59, m	2.21, overlap	2.51, dd (16.6, 3.4)	2.51, dd (16.5, 3.3)
6*β*	1.50, overlap	2.15, m	2.68, dd (16.6, 13.8)	2.65, overlap
7*α*	1.34, m	5.72, dt (6.5, 2.1)		
7*β*	1.76, m			
8	2.55, m			
11	6.86, d (2.1)	7.08, s	6.31, s	6.30, s
15*α*	1.78, overlap	1.62, m	2.48, m	2.74, dd (12.5, 5.7)
15*β*	1.69, overlap	1.80, overlap	1.46, td (13.8, 4.1)	1.48, m
16*α*	1.81, overlap	1.85, overlap	1.83, overlap	2.33, m
16*β*	1.66, overlap	1.85, overlap	1.94, m	2.27, dd (18.4, 5.8)
17	2.88, m	2.94, dd (7.2, 3.0)	2.21, overlap	
18a	10.33, s	10.37, s	5.09, d (2.4)	2.02, s
18b			4.96, d (2.4)	
19	1.13, s	1.17, s	1.26, s	1.24, s
20	2.42, m	2.60, overlap	1.38, overlap	2.92, m
21	1.10, d (6.8)	1.10, d (6.8)	0.95, d (6.7)	1.06, d (6.9)
22a	1.63, m	1.49, m	1.65, m	1.59, m
22b	1.30, m	1.21, overlap	1.24, m	1.54, m
23a	2.84, m	2.81, overlap	2.75, overlap	2.70, m
23b	2.61, m	2.60, m	2.75, overlap	2.65, overlap
24	5.90, m	5.84, brs	5.93, brs	5.98, t (7.7)
27	2.07, s	2.05, s	2.12, s	2.12, s
28	0.99, s	1.22, s	1.36, s	1.38, s
29	1.05, s	1.07, s	1.09, s	1.10, s
30	1.18, s	1.16, s	1.12, s	1.12, s

^a^Recorded at 500 MHz, in pyridine-*d*_5_. ^b^Recorded at 800 MHz, in pyridine-*d*_5_

**Table 2:** ^13^C NMR data of compounds **1**–**4** (*δ* in ppm)

No	1^a^	2^a^	3^a^	4^b^
1	35.7, CH_2_	35.4, CH_2_	34.9, CH_2_	35.0, CH_2_
2	34.6, CH_2_	34.8, CH_2_	34.5, CH_2_	34.6, CH_2_
3	214.8, C	214.1, C	213.9, C	214.1, C
4	47.9, C	47.8, C	47.3, C	47.3, C
5	52.5, CH	51.2, CH	51.3, CH	51.5, CH
6	22.1, CH_2_	23.9, CH_2_	36.8, CH_2_	36.8, CH_2_
7	25.8, CH_2_	118.2, CH	192.6, C	192.3, C
8	52.0, CH	149.8, C	144.6, C	141.6, C
9	169.6, C	163.6, C	169.7, C	170.6, C
10	38.4, C	36.3, C	36.2, C	36.2, C
11	116.5, CH	118.5, CH	120.2, CH	117.5, CH
12	173.8, C	171.9, C	168.5, C	169.1, C
13	128.7, C	128.6, C	146.4, C	123.2, C
14	45.9, C	44.2, C	54.0, C	50.1, C
15	31.6, CH_2_	28.7, CH_2_	31.2, CH_2_	31.1, CH_2_
16	19.1, CH_2_	18.9, CH_2_	23.8, CH_2_	22.7, CH_2_
17	36.3, CH	37.1, CH	50.7, CH	145.5, C
18	190.4, CH	190.0, CH	111.5, CH_2_	14.4, CH_3_
19	21.0, CH_3_	20.8, CH_3_	19.0, CH_3_	19.2, CH_3_
20	36.1, CH	36.0, CH	32.0, CH	35.8, CH
21	19.0, CH_3_	19.0, CH_3_	17.7, CH_3_	19.7, CH_3_
22	32.9, CH_2_	32.3, CH_2_	33.7, CH_2_	34.9, CH_2_
23	28.9, CH_2_	28.8, CH_2_	27.1, CH_2_	28.5, CH_2_
24	142.0, CH	142.1, CH	141.9, CH	141.8, CH
25	129.1, C	129.0, C	129.4, C	129.0, C
26	170.6, C	170.6, C	170.9, C	170.6, C
27	21.6, CH_3_	22.2, CH_3_	21.5, CH_3_	21.6, CH_3_
28	24.5, CH_3_	28.9, CH_3_	19.9, CH_3_	20.3, CH_3_
29	21.9, CH_3_	21.5, CH_3_	21.1, CH_3_	21.2, CH_3_
30	26.3, CH_3_	25.7, CH_3_	25.8, CH_3_	25.8, CH_3_

^a^Recorded at 125 MHz, in pyridine-*d*_5_. ^b^Recorded at 200 MHz, in pyridine-*d*_5_

**Fig. 1:**
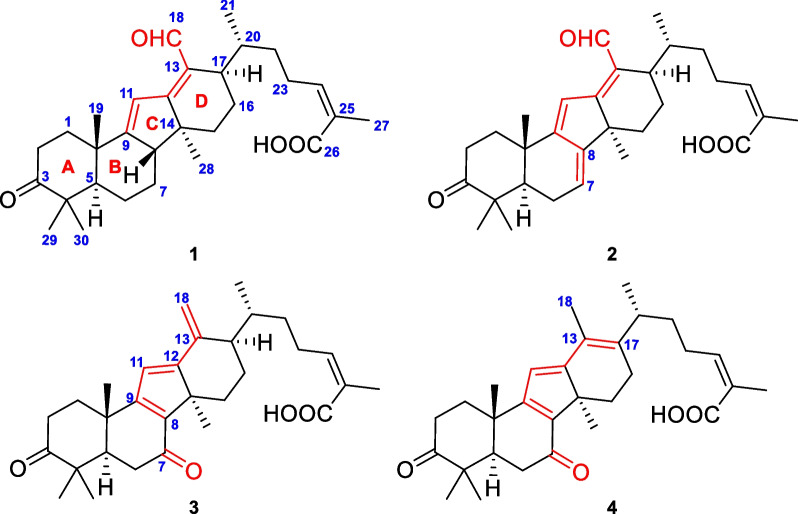
Chemical structures of kadcoccitanes E–H (**1**–**4**)

**Fig. 2:**
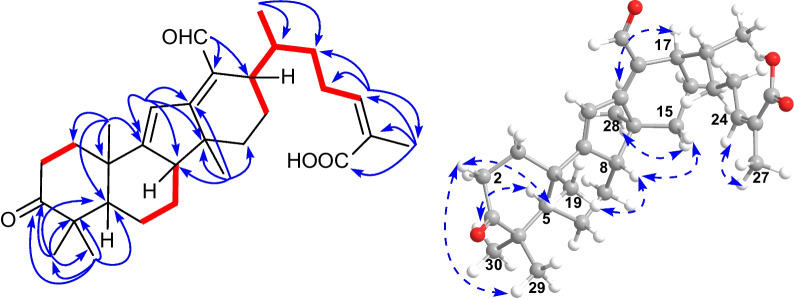
Key ^1^H–^1^H COSY (red and bold), HMBC (blue arrows), and ROESY (dashed arrows) correlations of **1**

Detailed analysis of the NMR spectra of **1** indicated that it was a 14(13 → 12)-abeolanostane triterpenoid featuring 6/6/5/6-fused ring system. The HMBC correlations (Fig. 2) from H_3_-19 (*δ*_H_ 1.13) to C-1 (*δ*_C_ 35.7), C-5 (*δ*_C_ 52.5), C-9 (*δ*_C_ 169.6), and C-10 (*δ*_C_ 38.4), from H_3_-29 (*δ*_H_ 1.05) to C-3 (*δ*_C_ 214.8), C-4 (*δ*_C_ 47.9), C-5, and C-30 (*δ*_C_ 26.3), and from H_3_-30 (*δ*_H_ 1.18) to C-3, C-4, C-5, and C-29 (*δ*_C_ 21.9), in combination with the ^1^H–^1^H COSY correlations (Fig. 2) of H-1*β* (*δ*_H_ 2.06)/H-2*β* (*δ*_H_ 2.71) and H-6*β* (*δ*_H_ 1.50)/H-7*α* (*δ*_H_ 1.34)/H-8 (*δ*_H_ 2.55) revealed the existence of typical rings A and B in kadcoccitane E (**1**). In addition, the HMBC correlations from H-11 (*δ*_H_ 6.86) to C-8 (*δ*_C_ 52.0), C-9, C-10, C-12 (*δ*_C_ 173.8), and C-14 (*δ*_C_ 45.9), from H_3_-28 (*δ*_H_ 0.99) to C-8, C-12, C-14, and C-15 (*δ*_C_ 31.6), and from H-18 (*δ*_H_ 10.33) to C-13 (*δ*_C_ 128.7) and C-17 (*δ*_C_ 36.3), in combination with the ^1^H–^1^H COSY correlations of H-16*α* (*δ*_H_ 1.81)/H-17 (*δ*_H_ 2.88) demonstrated that a six-membered ring with C-12/C-13 double bond and an aldehyde group at C-13 fused with a five-membered ring, which revealed the existence of rings C and D in kadcoccitane E. Furthermore, the HMBC correlations from H_3_-21 (*δ*_H_ 1.10) to C-17 and C-22 (*δ*_C_ 32.9), from H-24 (*δ*_H_ 5.90) to C-22, C-23 (*δ*_C_ 28.9), C-26 (*δ*_C_ 170.6), and C-27 (*δ*_C_ 21.6), and from H_3_-27 (*δ*_H_ 2.07) to C-24 (*δ*_C_ 142.0), C-25 (*δ*_C_ 129.1), and C-26, in combination with the ^1^H–^1^H COSY correlations of H-17/H-20 (*δ*_H_ 2.42)/H-22b (*δ*_H_ 1.30)/H-23a (*δ*_H_ 2.84)/H-24 revealed the existence of typical side chain (C-20–C-27) in kadcoccitane E (**1**). Thus, the planar structure of **1** could be established as shown in Fig. 1.

With respect to the stereochemistry of **1**, the C-24/C-25 double bond was assigned *Z* geometry due to the H-24/H_3_-27 correlation in the ROESY spectrum (Fig. 2). Besides, correlations of H_3_-30/H-5*α* (*δ*_H_ 1.49), H-2*β*/H_3_-29, H_3_-19/H-2*β*, H-8/H_3_-19, H-15*β* (*δ*_H_ 1.69)/H-8, H_3_-28/H-15*α* (*δ*_H_ 1.78), H-17/H_3_-28 indicated that both H-8 and H_3_-19 adopted *β*-orientations, while both H-17 and H_3_-28 adopted *α*-orientations. According to these deductions, as well as biosynthetic considerations, the absolute configuration of **1** was determined as 5*R*, 8*S*, 10*S*, 14*S*, 17*R*, and 20*R*.

**Fig. 3:**
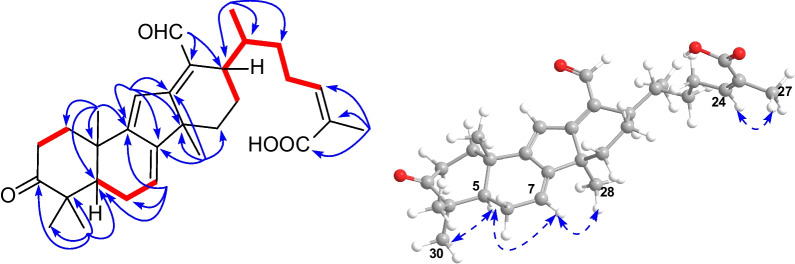
Key ^1^H–^1^H COSY (red and bold), HMBC (blue arrows), and ROESY (dashed arrows) correlations of **2**

Kadcoccitane F (**2**) was obtained as a light yellow amorphous solid and its molecular formula was determined as C_30_H_40_O_4_ by its HRESIMS data ([M − H]^–^ *m/z* 463.2852, calcd 463.2854). The ^1^H NMR spectrum (Table 1) displayed signals for three olefinic protons (*δ*_H_ 5.72, dt, *J* = 6.5, 2.1 Hz; *δ*_H_ 5.84, brs; *δ*_H_ 7.08, s), an aldehyde group (*δ*_H_ 10.37, s), a doublet methyl group (*δ*_H_ 1.10, d, *J* = 6.8 Hz), and five singlet methyl groups (*δ*_H_ 1.07, 1.16, 1.17, 1.22, and 2.05). The ^13^C NMR and DEPT spectra (Table 2) showed 30 carbon resonances, including six methyls, seven methylenes, seven methines (three olefinic and one aldehyde), and ten nonprotonated carbons (one carboxyl, one carbonyl, five olefinic, and three quaternary carbons). Apart from the seven DOUs generated by carboxyl, carbonyl, aldehyde and olefinic groups, the remaining four ones manifested the tetracyclic system of **2**. Analysis of the NMR spectra of **2** indicated that its structure was closely similar to that of **1**, except that **2** possessed one additional double assumed to be located in ring B. This deduction could be supported by HMBC correlations (Fig. 3) from H-7 (*δ*_H_ 5.72) to C-5 (*δ*_C_ 51.2), C-6 (*δ*_C_ 23.9), C-9 (*δ*_C_ 163.6), and C-14 (*δ*_C_ 44.2), as well as ^1^H–^1^H COSY correlations (Fig. 3) of H-5 (*δ*_H_ 1.82)/H-6*β* (*δ*_H_ 2.15)/H-7. Upon carefully analyzing the ROESY spectrum of **2**, in combination with comparisons between NMR data of **2** and **1**, and biosynthetic considerations, the absolute configuration of **2** was determined as 5*R*, 10*S*, 14*S*, 17*R*, and 20*R*.

Kadcoccitane G (**3**) was isolated as a light yellow amorphous solid and its molecular formula was determined as C_30_H_40_O_4_, with eleven DOUs based on its HRESIMS data ([M – H]^–^ *m/z* 463.2854, calcd 463.2854). The ^1^H NMR spectrum (Table 1) showed signals for four olefinic protons (*δ*_H_ 4.96, d, *J* = 2.4 Hz; *δ*_H_ 5.09, d, *J* = 2.4 Hz; *δ*_H_ 5.93, brs; *δ*_H_ 6.31, s), a doublet methyl group (*δ*_H_ 0.95, d, *J* = 6.7 Hz), and five singlet methyl groups (*δ*_H_ 1.09, 1.12, 1.26, 1.36, and 2.12). Totally 30 carbon signals could be observed in the ^13^C NMR and DEPT spectra (Table 2), including six methyls, eight methylenes (one olefinic), five methines (two olefinic), and eleven nonprotonated carbons (one carboxyl, two carbonyl, five olefinic, and three quaternary carbons). Apart from the seven DOUs generated by carboxyl, carbonyl and olefinic groups, the remaining four ones disclosed the existence of four rings in its structure.

**Fig. 4:**
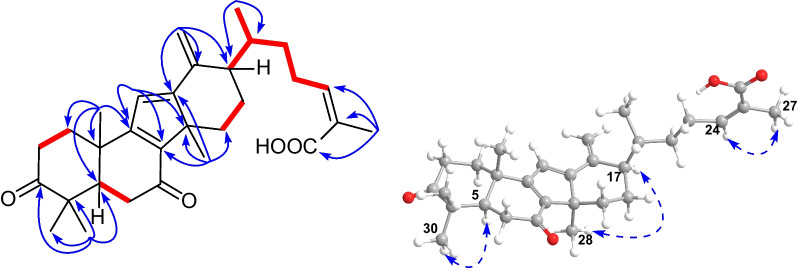
Key ^1^H–^1^H COSY (red and bold), HMBC (blue arrows), and ROESY (dashed arrows) correlations of** 3**

Further analysis of the 1D and 2D NMR spectra of **3** indicated that it possessed the same scaffold as kadcoccitanes E and F (**1** and **2**). The HMBC correlations (Fig. 4) from H-11 (*δ*_H_ 6.31) to C-8 (*δ*_C_ 144.6), C-9 (*δ*_C_ 169.7) and C-14 (*δ*_C_ 54.0), from H_3_-28 (*δ*_H_ 1.36) to C-8, C-12 (*δ*_C_ 168.5), C-14, and C-15 (*δ*_C_ 31.2), and from H_2_-18 (*δ*_H_ 4.96, 5.09) to C-12, C-13 (*δ*_C_ 146.4) and C-17 (*δ*_C_ 50.7), in combination with the ^1^H–^1^H COSY correlations (Fig. 4) of H-5 (*δ*_H_ 2.39)/H-6*β* (*δ*_H_ 2.68), H-15*α* (*δ*_H_ 2.48)/H-16*α* (*δ*_H_ 1.83)/H-17 (*δ*_H_ 2.21) uncovered the existence of an *α*,*β*,*γ*,*δ*,ε,ζ-unsaturated ketone system distributed in rings B–D. The ROESY correlations (Fig. 4) suggested that **3** possessed identical relative configuration as that of **2**. Finally, the absolute configuration of **3** was determined as 5*R*, 10*S*, 14*R*, 17*R*, and 20*R* through biosynthetic considerations.

Kadcoccitane H (**4**) was obtained as a yellow amorphous solid and had the molecular formula of C_30_H_40_O_4_ as determined by its HRESIMS data ([M + H]^+^ *m/z* 465.2999, calcd 465.2999). The ^1^H NMR spectrum (Table 1) showed signals for two olefinic protons (*δ*_H_ 5.98, t, *J* = 7.7 Hz; *δ*_H_ 6.30, s), a doublet methyl group (*δ*_H_ 1.06, d, *J* = 6.9 Hz), and six singlet methyl groups (*δ*_H_ 1.10, 1.12, 1.24, 1.38, 2.02 and 2.12). The ^13^C NMR and DEPT spectra (Table 2) exhibited 30 carbon signals, including seven methyls, seven methylenes, four methines (two olefinic), and twelve nonprotonated carbons (one carboxyl, two carbonyl, six olefinic, and three quaternary carbons). Apart from the seven DOUs occupied by carboxyl, carbonyl and olefinic groups, the remaining four ones revealed that **4** had a tetracyclic structure.

**Fig. 5:**
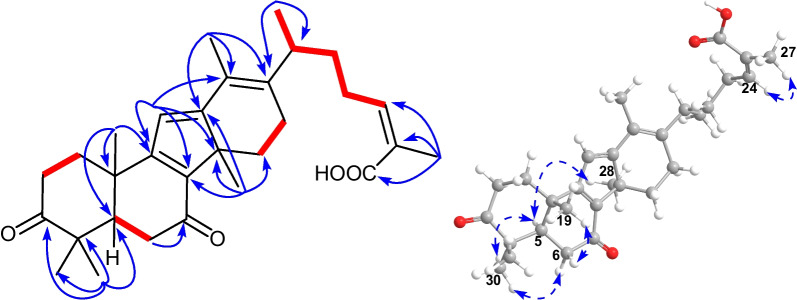
Key ^1^H–^1^H COSY (red and bold), HMBC (blue arrows), and ROESY (dashed arrows) correlations of** 4**

An in-depth analysis of the 1D and 2D NMR spectra of **4** indicated that its structure resembled closely to that of **3**, and only one variation could be observed. The exocyclic C-13/C-18 double bond in **3** became C-13/C-17 double bond in **4**, which could be revealed by HMBC correlations (Fig. 5) from H_3_-18 (*δ*_H_ 2.02) to C-12 (*δ*_C_ 169.1), C-13 (*δ*_C_ 123.2) and C-17 (*δ*_C_ 145.5).

**Fig. 6:**
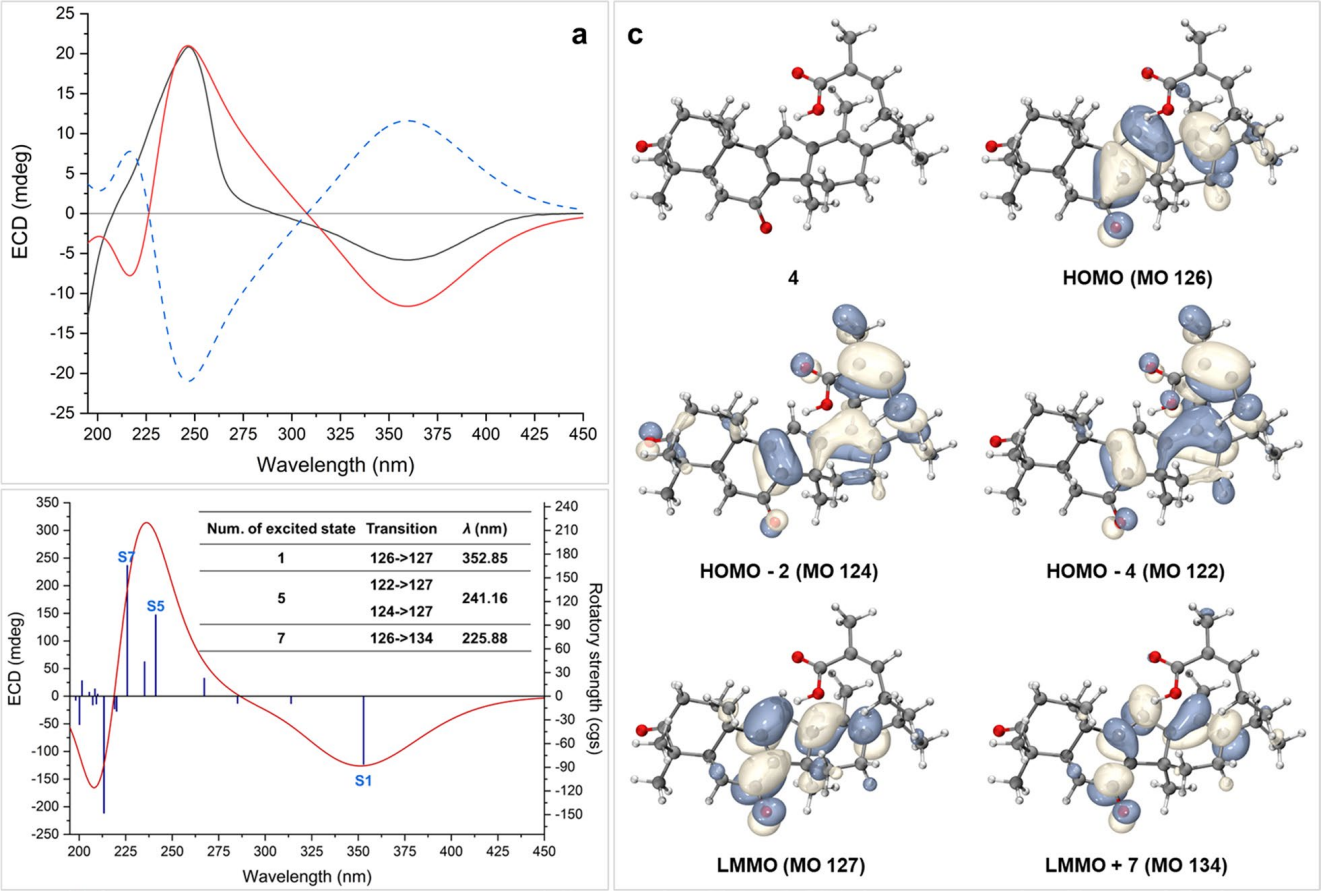
**a** Experimental ECD spectrum of **4** (black). Calculated ECD spectra (shift = + 8 nm) of (5*R*,10*S*,14*R*,20*R*)-**4** (**4aA**) (red) and *ent*-**4aA** (blue). **b** Calculated ECD spectrum (curve) of conformer **4a-1** with rotatory strength (bar), and the key transitions in three important excited states (table). **c** Key MOs of conformer **4a-1**

The relative configuration of rings A–D in **4** was elucidated to be the same as that in **3** through analysis of the ROESY spectrum (Fig. 5) and NMR data comparison. However, it is difficult to determine the configuration of C-20 which was located on the conformationally flexible side chain as well as on an allyl position. Thus, (5R*, 10S*, 14R*, 20R*)-**4** (**4a**) and (5R*, 10S*, 14R*, 20S*)-**4** (**4b**) were subjected to GIAO NMR calculations at mPW1PW91-SCRF/6–31 + G(d,p)//B3LYP-D3/6-31G(d) level of theory. Then, considering the flexibility of compound **4**, DP4 + analysis based on random conformational amplitudes [12] was employed to distinguish **4a** and **4b**. As a result, **4a** and **4b** got a probability of 67.1% and 32.9%, respectively (Fig. S53). In addition, as the calculated chemical shifts of the *α*,*β*-unsaturated carboxyl moiety usually endure relatively large errors, which is also the case in the present study, an additional DP4 + analysis was undertaken using partial data in which ^1^H and ^13^C chemical shifts/shielding tensors in C-24–C-27 moiety were excluded from experimental/calculated data. As a result, **4a** and **4b** got a probability of 66.1% and 33.9%, respectively (Fig. S54). Thus, the relative configuration of **4** was determined as 5R*,10S*,14R*, and 20R*. Subsequent TDDFT ECD calculation on (5*R*,10*S*,14*R*,20*R*)-**4** (**4aA**) afforded a theoretical curve which matched with the experimental spectrum very well (Fig. 6a). Then, the dominant conformer **4a-1** was subjected to molecular orbital (MO) analysis, and the π → π* transition from HOMO to LUMO MOs contributed significantly to the Cotton effect around 350 nm (Fig. 6b and c). Finally, the absolute configuration of **4** was determined as 5*R*, 10*S*, 14*R*, and 20*R*.

The potential cytotoxic activities of kadcoccitanes E–H (**1**–**4**) against five human tumor cell lines, HL-60 (leukemia), A-549 (lung cancer), SMMC-7721 (liver cancer), MDA-MB-231 (breast cancer), and SW-480 (colon cancer) were tested, but no remarkable activities were observed (Table 3).

**Table 3:** Cytotoxicity of compounds **1**–**4** against five human tumor cell lines, cell inhibition (%)

No	HL-60		A-549		SMMC-7721		MDA-MB-231		SW-480	
	Average	SD	Average	SD	Average	SD	Average	SD	Average	SD
1	1.93	1.83	8.33	0.69	17.95	1.89	6.47	1.98	− 4.65	3.00
2	–29.46	0.80	12.39	1.36	25.06	1.95	6.98	1.90	7.13	2.76
3	–44.33	1.19	17.38	2.36	22.99	2.62	1.93	0.12	11.42	1.39
4	7.09	3.55	9.52	2.02	6.97	1.80	3.74	0.25	2.66	1.92

## Experimental

### General experimental procedures

General experimental procedures can be found in the Supplementary Material (part 1).

### Plant material

The source of the plant material is the same as described in ref 9 or can be found in the Supplementary Material (part 2), but the investigated parts in the present research are stems, instead of roots in ref 9.

### Extraction and isolation

The detailed procedures for extraction and isolation can be found in in the Supplementary Material (part 3).

### Characteristic data of compounds 1–4

Kadcoccitane E (**1**): light yellow amorphous powder; [*α*]_D_^23^ − 73.57 (*c* 0.107, MeOH); UV(MeOH) *λ*_max_ (log *ε*): 195 (4.16), 217 (4.02), 265 (3.57), 304 (4.13) nm; ECD (MeOH) *λ*_max_ (Δ*ε*): 195 (+ 8.00), 223 (− 15.29), 303 (+ 12.01), 343 (− 8.88) nm; IR (KBr) *ν*_max_ 3407, 2951, 1707, 1658, 1605, 1205, 1132, 869, 577 cm^−1^; negative HRESIMS *m/z* 465.3014 [M − H]^–^ (calcd for C_30_H_41_O_4_, 465.3010).

Kadcoccitane F (**2**): light yellow amorphous powder; [*α*]_D_^25^ − 51.09 (*c* 0.092, MeOH); UV(MeOH) *λ*_max_ (log *ε*): 195 (4.17), 272 (3.53), 337 (3.94) nm; ECD (MeOH) *λ*_max_ (Δ*ε*): 202 (+ 4.30), 229 (− 4.93), 256 (+ 2.36), 322 (+ 5.08), 368 (− 4.93) nm; IR (KBr) *ν*_max_ 3427, 2962, 2933, 2872, 1706, 1652, 1381, 1204, 581 cm^−1^; negative HRESIMS *m/z* 463.2852 [M − H]^–^ (calcd for C_30_H_39_O_4_, 463.2854).

Kadcoccitane G (**3**): light yellow amorphous powder; [*α*]_D_^19^ + 103.68 (*c* 0.086, MeOH); UV(MeOH) *λ*_max_ (log *ε*): 195 (4.25), 270 (3.20), 318 (3.90) nm; ECD (MeOH) *λ*_max_ (Δ*ε*): 195 (+ 5.48), 217 (− 2.60), 337 (+ 5.16) nm; IR (KBr) *ν*_max_ 3430, 2964, 2935, 2872, 1707, 1645, 1457, 1392, 1245, 596 cm^−1^; negative HRESIMS *m/z* 463.2854 [M − H]^–^ (calcd for C_30_H_39_O_4_, 463.2854).

Kadcoccitane H (**4**): yellow amorphous powder; [*α*]_D_^20^ –275.76 (*c* 0.198, MeOH); UV(MeOH) *λ*_max_ (log *ε*): 199 (4.04), 224 (4.19), 285 (2.95), 368 (4.01) nm; ECD (MeOH) *λ*_max_ (Δ*ε*): 195 (–15.23), 247 (+ 24.69), 358 (–6.89) nm; IR (KBr) *ν*_max_ 3428, 2963, 2931, 1708, 1638, 1394, 1191, 1100, 601, 575 cm^−1^; positive HRESIMS *m/z* 465.2999 [M + H]^+^ (calcd for C_30_H_41_O_4_, 465.2999).

For ^1^H NMR data of kadcoccitanes E–H (**1**–**4**), see Table 1. For their ^13^C NMR data, see Table 2.

### The cytotoxicity assay

The cytotoxicity assay has been described previously [13].

## Supplementary Information

Below is the link to the electronic supplementary material.

Supplementary file1 It includes general experimental procedures; plant material; extraction and isolation; 1D NMR, 2D NMR, HRESIMS, UV, ECD, IR spectra, and OR of compounds 1–4, as well as computational methods and data for compound 4.

## Data Availability

The data that support the findings of this study are openly available in the Science Data Bank at https://doi.org/10.57760/sciencedb.j00144.00004 (Under review).
