# Supplementary material for: Four new lanostane triterpenoids featuring extended π-conjugated systems from the stems of Kadsura coccinea
Source: Nat Prod Bioprospect. 2023 Apr 6;13(1):12. doi: 10.1007/s13659-023-00376-1 (PMC10076471; doi:10.1007/s13659-023-00376-1)
Supplement: Supplementary file 1 — Supplementary file1 It includes general experimental procedures; plant material; extraction and isolation; 1D NMR, 2D NMR, HRESIMS, UV, ECD, IR spectra, and OR of compounds 1–4, as well as computational methods and data for compound 4. [file 13659_2023_376_MOESM1_ESM.pdf]

Supplementary Material

Four new lanostane triterpenoids featuring extended π-conjugated systems from the stems of *Kadsura coccinea*

Qi-Qi Zhang[1,2], Kun Hu[1], Han-Dong Sun[1], Pema-Tenzin Puno[1,*]

[1] State Key Laboratory of Phytochemistry and Plant Resources in West China, Kunming Institute of Botany, Chinese Academy of Sciences, and Yunnan Key Laboratory of Natural Medicinal Chemistry, Kunming 650201, Yunnan, People's Republic of China

[2] University of Chinese Academy of Sciences, Beijing 100049, People's Republic of China

[*]Corresponding author tel.: (86) 871-65223616

E-mail: punopematenzin@mail.kib.ac.cn

Contents of Supplementary Material

1. General experimental procedures 3

2. Plant material 3

3. Extraction and isolation 3

4. NMR, MS, UV, ECD, IR spectra, and OR of kadcoccitane E (1) 4

5. NMR, MS, UV, ECD, IR spectra, and OR of kadcoccitane F (2) 12

6. NMR, MS, UV, ECD, IR spectra, and OR of kadcoccitane G (3) 19

7. NMR, MS, UV, ECD, IR spectra, and OR of kadcoccitane H (4) 26

8. Computational data of 4a and 4b. 33

1. General experimental procedures

1D and 2D NMR spectra were recorded on Bruker AV III 500 MHz or Bruker Ascend 800 MHz spectrometers (Bruker Corp., Switzerland) with TMS as internal standard. In general, chemical shifts (δ) are expressed in ppm with reference to the solvent signals for pyridine-d_5 (δ_H 8.73/δ_C 149.9). HRESIMS data were performed on an Agilent 6540 QSTAR TOF time-of-flight mass spectrometer (Agilent Corp., America). A Tenor 27 spectrophotometer was used for IR spectra (Bruker Corp., Switzerland), using KBr pellets. Optical rotations were measured with Horiba SEPA-300 and Jasco P-1020 polarimeters, respectively. ECD spectra were measured on a Chirascan V100 instrument (Applied Photophysics Limited, Britain). UV spectra were obtained using a Shimadzu UV-2401A spectrophotometer. Preparative and semi-preparative HPLC were performed on Agilent 1100/1200/1260 liquid chromatograph with ZORBAX SB-C18 (9.4 mm ×250 mm) column or COSMOSIL π NAP (10 ID × 250 mm) columns. Column chromatography (CC) was performed using silica gel (80–100 mesh and 100–200 mesh, Qingdao Marine Chemical, Inc., Qingdao, P. R. China), Lichroprep RP-18 gel (40–63 μm, Merck, Darmstadt, Germany), and MCI gel (75–150 μm, Mitsubishi Chemical Corporation, Tokyo, Japan). Fractions were monitored by thin layer chromatography (TLC), which was carried out on silica gel 60 F254 on glass plate (Qingdao Marine Chemical, Inc.). Spots were visualized by UV light (254 nm) and by heating silica gel plates sprayed with 10% H_2SO_4 in ethanol. All solvents used in column chromatography were distilled.

2. Plant material

The stems of *Kadsura coccinea* (Lem.) A. C. Smith were collected in Jingzhou Miao and Dong Autonomous County in Hunan Province, People's Republic of China, in June 2016 and identified by Prof. Heng Li, Kunming Institute of Botany. A voucher specimen (KIB 2016062101) has been deposited in the State Key Laboratory of Phytochemistry and Plant Resources in West China, Kunming Institute of Botany, Chinese Academy of Sciences.

3. Extraction and isolation

The air-dried stems of *K. coccinea* (8 Kg) were extracted four times (3 days each time) with 70% aqueous acetone (50 L) at room temperature and concentrated at reduced pressure to afford a crude

extract, which was partitioned between H_2O and EtOAc. The EtOAc extract (360 g) was eluted with $CHCl_3/Me_2CO$ (1:0–0:1, v/v; gradient system) using silica gel column (4 kg, 80–100 mesh) to give seven fractions (Fr. A−Fr. G).

Fr. B (111 g) was decolorized on MCI gel with 90% MeOH, then on RP-18 silica gel CC ($MeOH/H_2O$, 30% to 100%), to afford subfractions (Fr. B1−Fr. B26). Fr. B11 (2 g) was further fractionated into six subfractions (Fr. B11-1−Fr. B11-6) by silica gel CC ($CHCl_3$/MeOH, 1:0−0:1) based on TLC analysis, and Fr. B11-4(250mg) was separated by preparative HPLC with the same gradient elution (65%, $MeCN/H_2O$, 5 mL/min), followed by semi-preparative HPLC (60%, $MeCN/H_2O$, 3.0 mL/min), to afford compounds **3** (3.0 mg), **4** (35.0 mg). Fr. B12 (4 mg) was separated by silica gel CC (petroleum ether/Me_2CO 0:1–0:1) to give thirty-three minor subfractions (Fr. B12-1−Fr. B12-33) based on TLC analysis. Fr. B12-19 (380 mg) was purified by preparative HPLC with the same gradient elution (75%, $MeCN/H_2O$, 5.0 mL/min) to afford compounds **1** (30.0 mg) and **2** (4.0 mg).

4. NMR, MS, UV, ECD, IR spectra, and OR of kadcoccitane E (1)

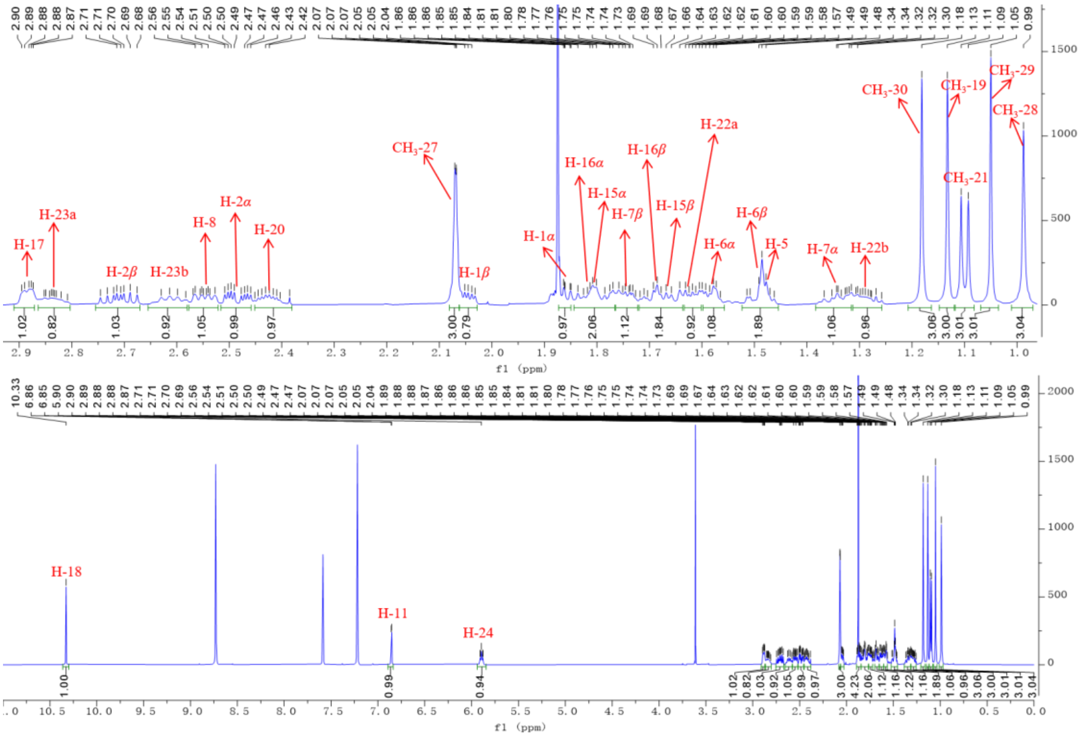

Figure S1 ^{1}H NMR spectrum of kadcoccitane E (**1**) (pyridine-d_5, 500 MHz).

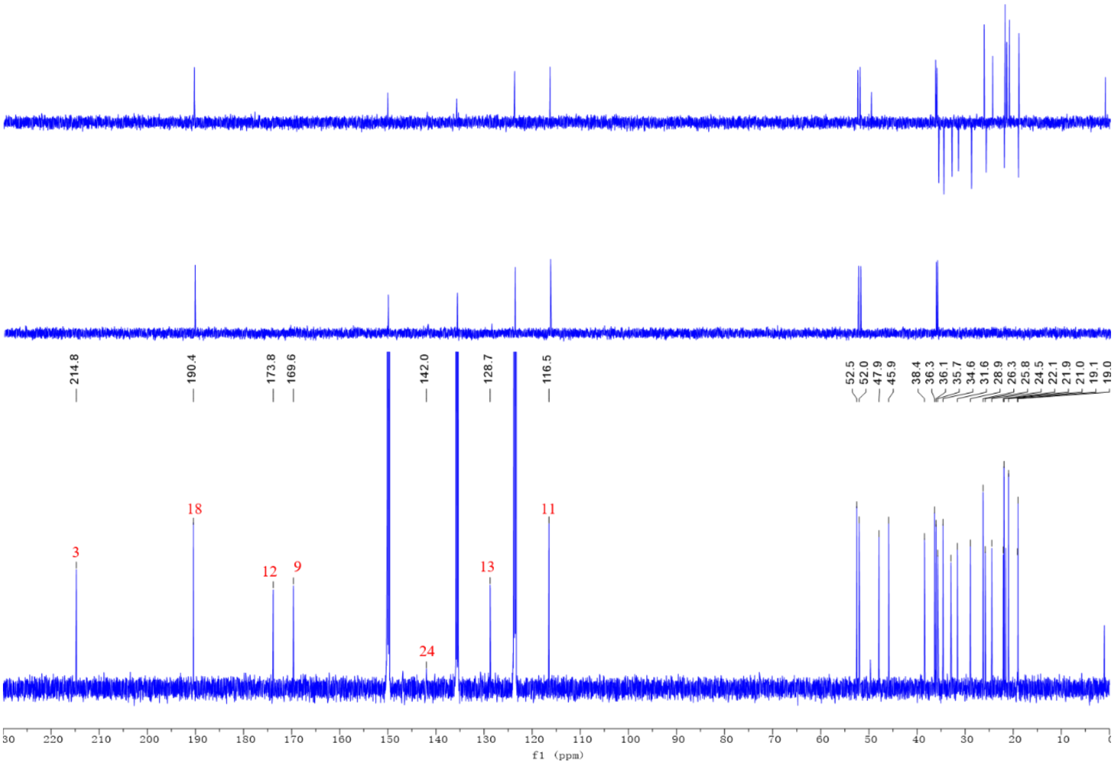

Figure S2 ^{13}C NMR spectrum of kadcoccitane E (**1**) (pyridine-d_5, 125 MHz).

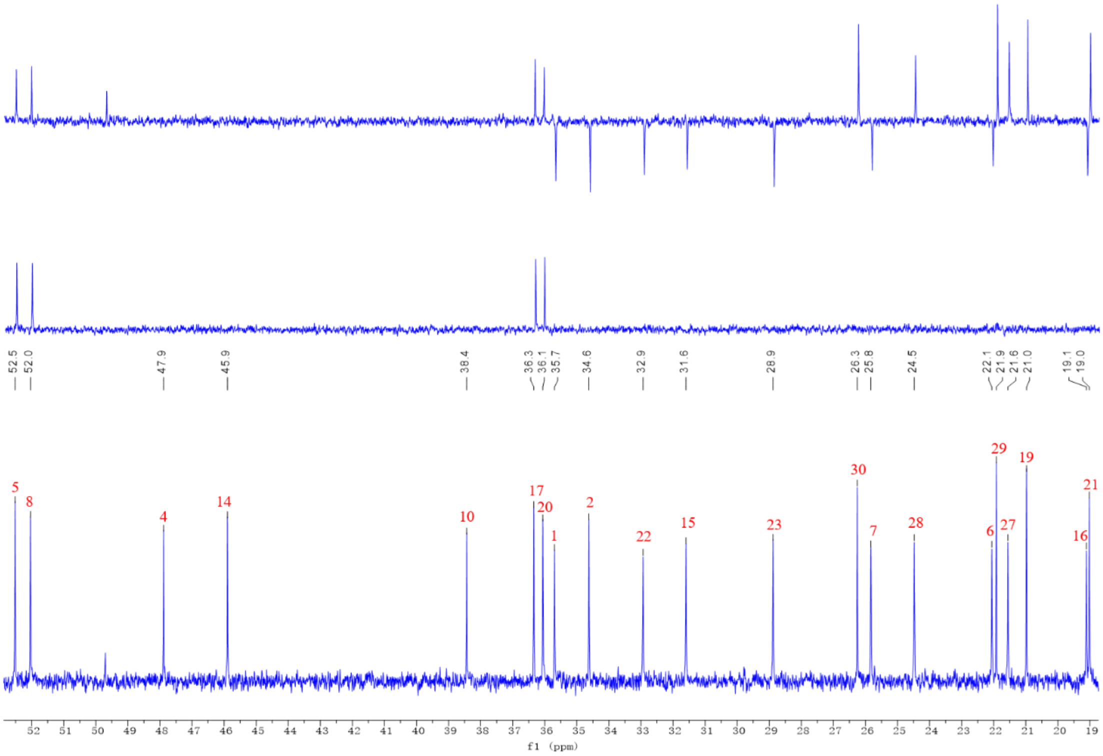

Figure S3 ^{13}C NMR spectrum of kadcoccitane E (**1**) (pyridine-d_5, 125 MHz).

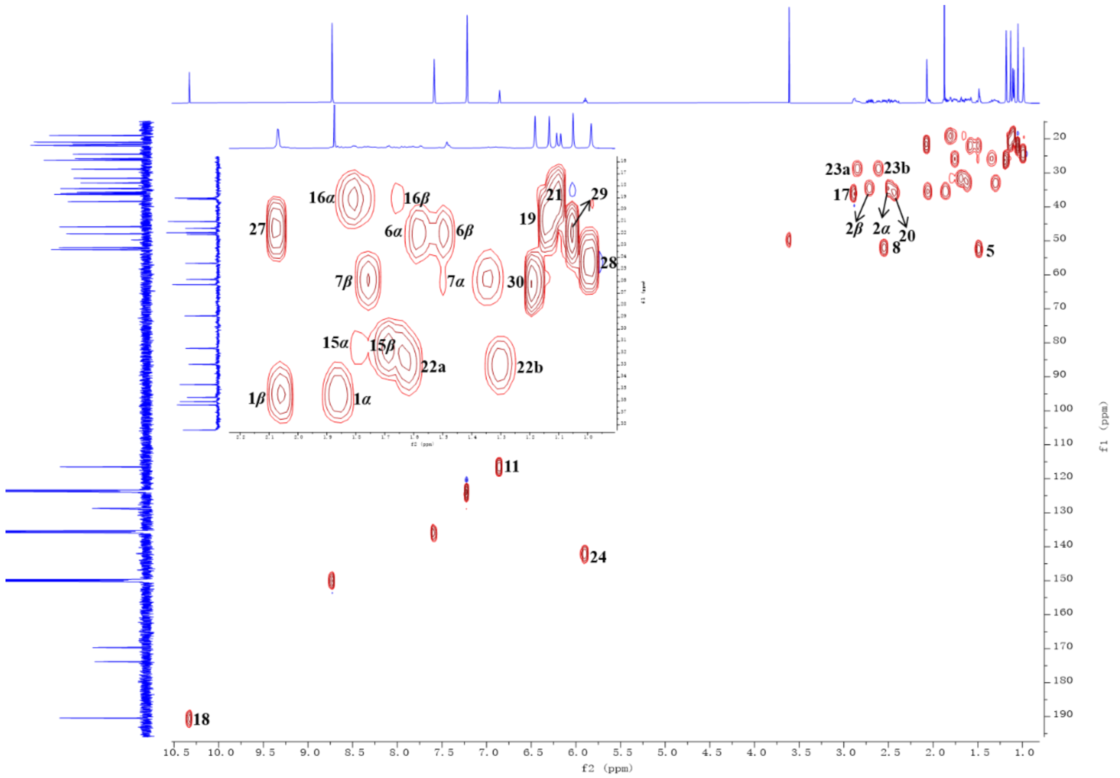

Figure S4 HSQC spectrum of kadcoccitane E (**1**) (pyridine-d_5, 500 MHz).

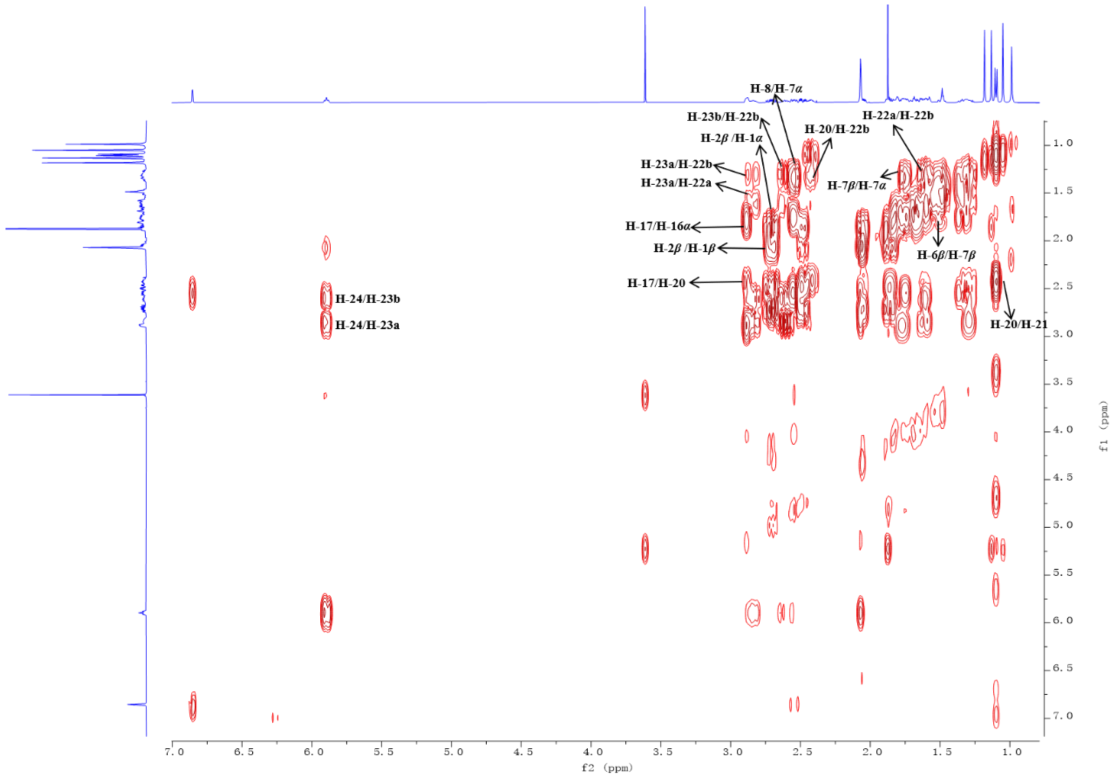

Figure S5 ^{1}H–^{1}H COSY spectrum of kadcoccitane E (**1**) (pyridine-d_5, 500 MHz).

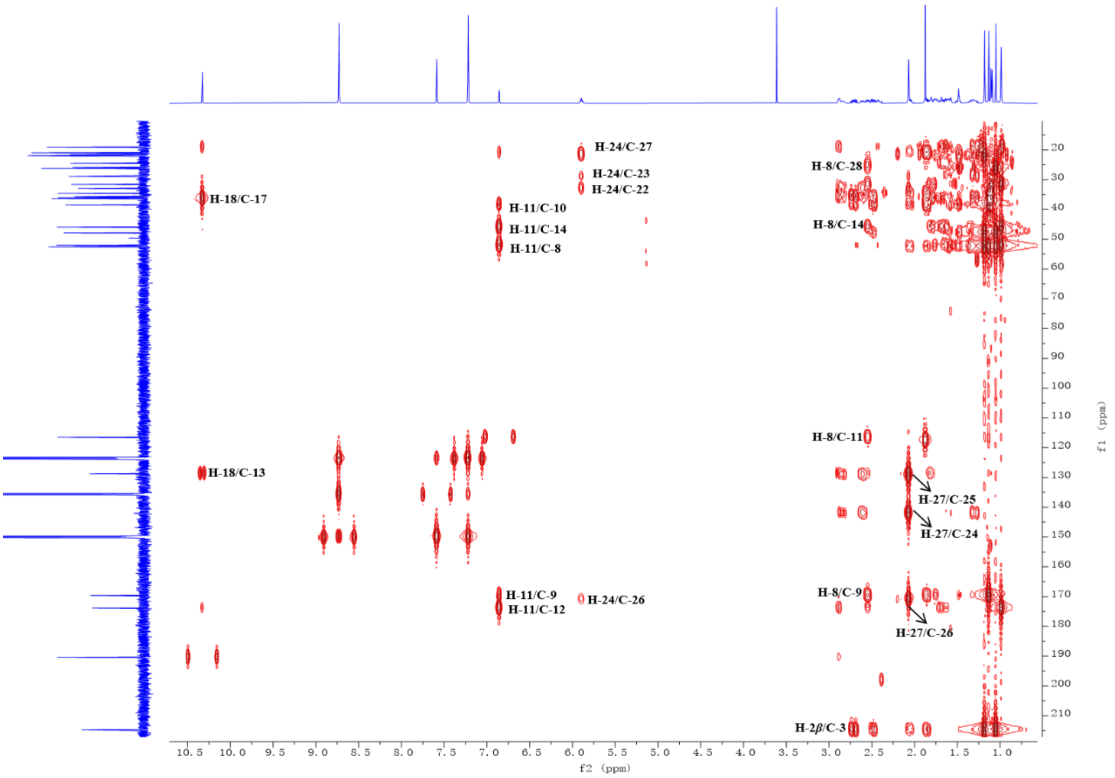

Figure S6 HMBC spectrum of kadcoccitane E (**1**) (pyridine-d_5, 500 MHz).

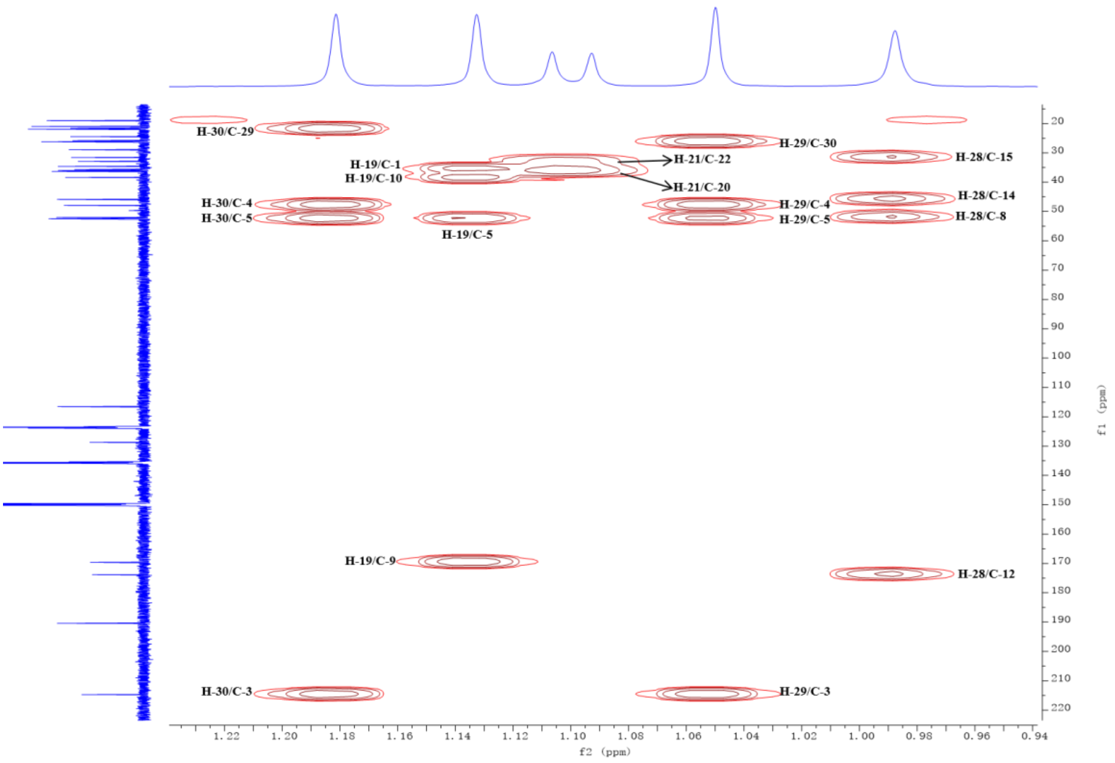

Figure S7 Locally magnified HMBC spectrum of kadcoccitane E (**1**) (pyridine-d_5, 500 MHz).

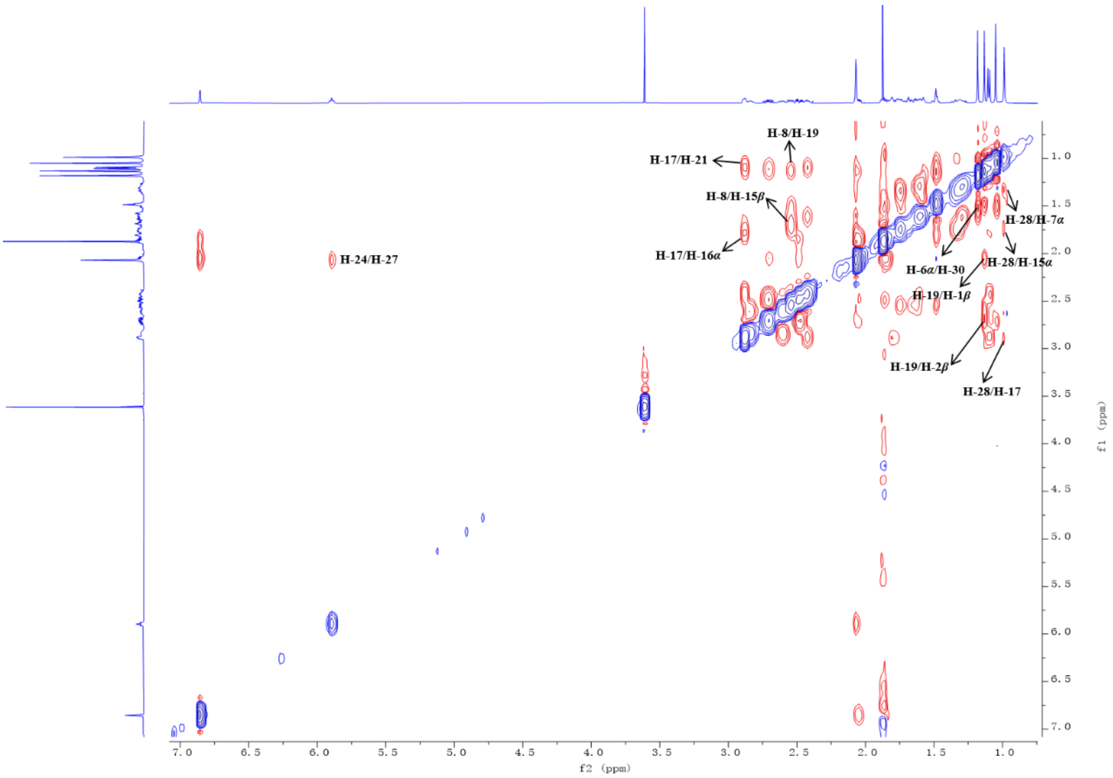

Figure S8 ROESY spectrum of kadcoccitane E (**1**) (pyridine-d_5, 500 MHz).

Qualitative Analysis Report

**Data Filename**	szqq-33NA.d	**Sample Name**	szqq-33NA
**Sample Type**	Sample	**Position**	P1-A5
**Instrument Name**	Instrument 1	**User Name**	
**Acq Method**	s-.m	**Acquired Time**	3/16/2022 4:25:25 PM
**IRM Calibration Status**	Success	**DA Method**	PCDL.m
**Comment**			

**Sample Group**		**Info.**
**Acquisition SW Version**	6200 series TOF/6500 series Q-TOF B.05.01 (B5125.2)	

User Spectra

Fragmentor Voltage	Collision Energy	Ionization Mode
185	0	ESI

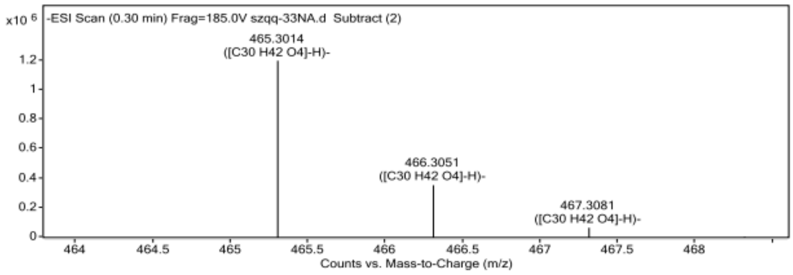

Peak List

m/z	z	Abund	Formula	Ion
465.3014	1	1198316.25	C30 H42 O4	(M-H)-
466.3051	1	357557.97	C30 H42 O4	(M-H)-
467.3081	1	66524.01	C30 H42 O4	(M-H)-
533.2881	1	32926.07		
550.2786	1	33255.34		
931.6078	1	88673.16		
932.6109	1	56780.02		
953.5897	1	33294.09		
1386.3019	1	69675.23		
1387.3059	1	35878.7		

Formula Calculator Element Limits

Element	Min	Max
C	3	60
H	0	120
O	0	30

Formula Calculator Results

Formula	CalculatedMass	CalculatedMz	Mz	Diff. (mDa)	Diff. (ppm)	DBE
C30 H42 O4	466.3083	465.3010	465.3014	-0.40	-0.86	10.0000

--- End Of Report ---

Figure S9 HRESIMS spectrum of kadcoccitane E (**1**).

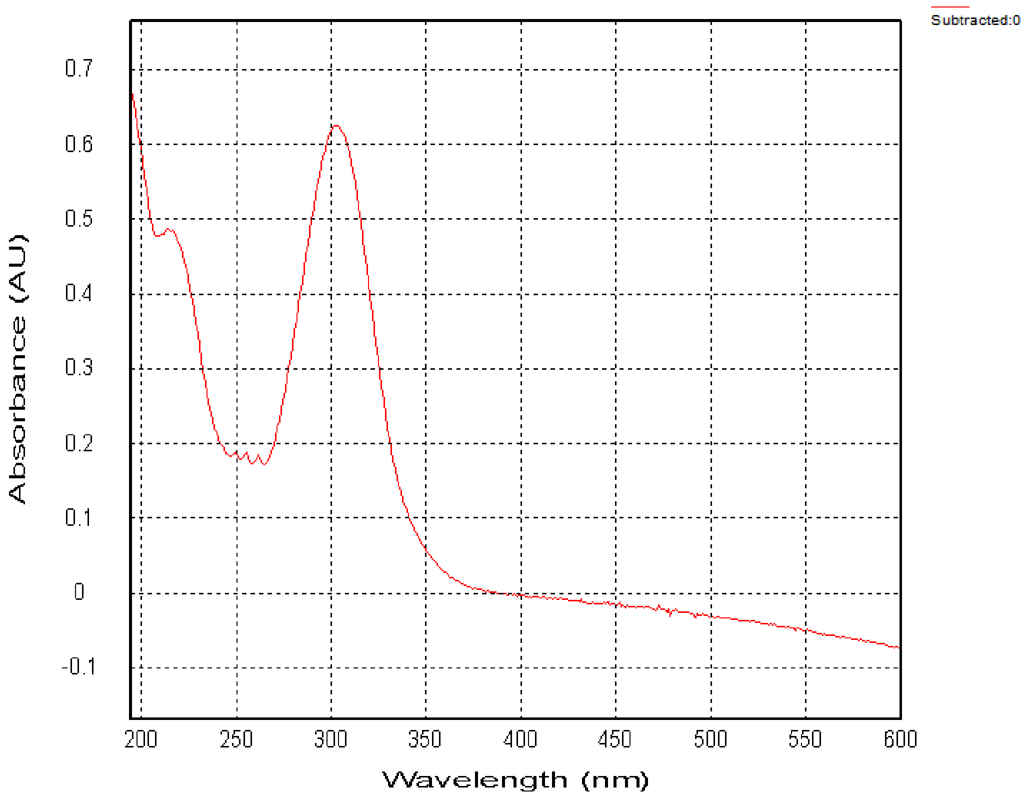

Figure S10 UV spectrum of kadcoccitane E (**1**).

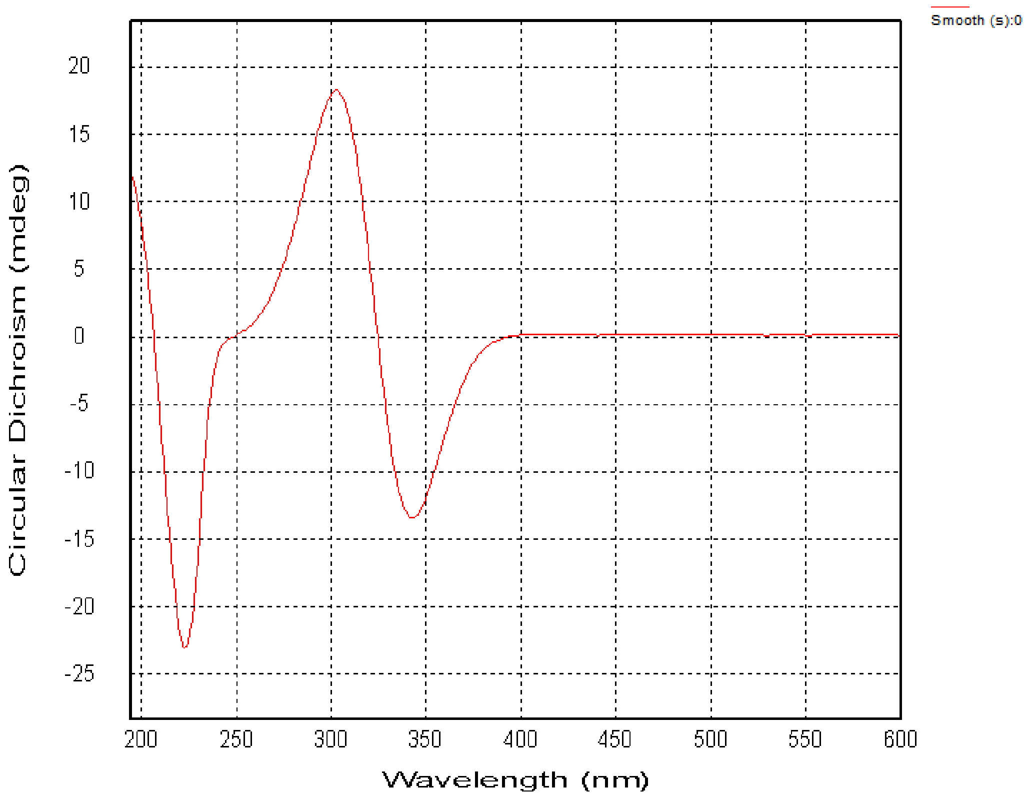

Figure S11 ECD spectrum of kadcoccitane E (**1**).

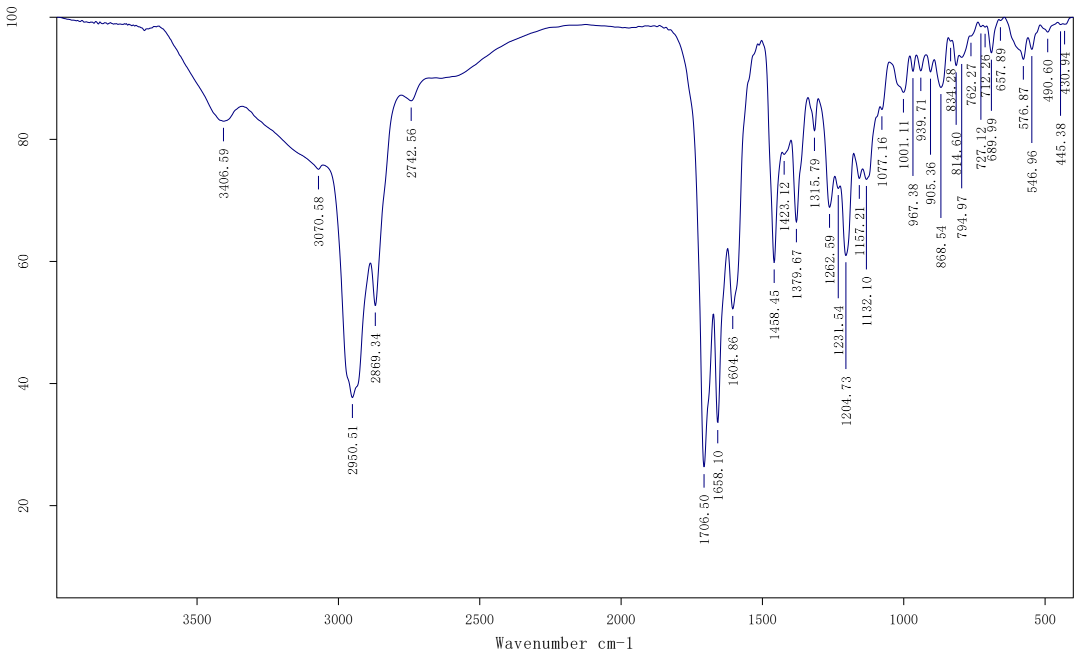

Sample Name: szqq-33NA	Resolution: 4	Beamsplitter Setting: KBr
Sample Form: KBr	Aperture Setting: 6 mm	Source Setting: MIR
Path of File: E:\data	Number of Background Scans: 16	Instrument Type: BRUKER VERTEX 70
Date of Measurement: 2022/3/28	Number of Sample Scans: 16	Soft Version: OPUS8.1

Figure S12 IR spectrum of kadcoccitane E (**1**).

Rudolph Research Analytical

This sample was measured on an Autopol VI, Serial #91058
Manufactured by Rudolph Research Analytical, Hackettstown, NJ, USA.

Measurement Date : Thursday, 24-MAR-2022

Set Temperature : OFF

Time Delay : Disabled

Delay between Measurement : Disabled

n	Average	Std.Dev.	% RSD	Maximum	Minimum
5	-73.57	2.79	-3.79	-70.93	-77.10

S.No	Sample ID	Time	Result	Scale	OR °Arc	WLG.nm	Lg.mm	Conc.g/100ml	Temp.
1	SZQQ-33NA	02:10:49 PM	-75.98	SR	-0.0813	589	100.00	0.107	23.0
2	SZQQ-33NA	02:10:57 PM	-77.10	SR	-0.0825	589	100.00	0.107	23.0
3	SZQQ-33NA	02:11:16 PM	-72.34	SR	-0.0774	589	100.00	0.107	22.9
4	SZQQ-33NA	02:11:24 PM	-71.50	SR	-0.0765	589	100.00	0.107	22.9
5	SZQQ-33NA	02:11:32 PM	-70.93	SR	-0.0759	589	100.00	0.107	22.9

Figure S13 OR of kadcoccitane E (**1**).

5. NMR, MS, UV, ECD, IR spectra, and OR of kadcoccitane F (2)

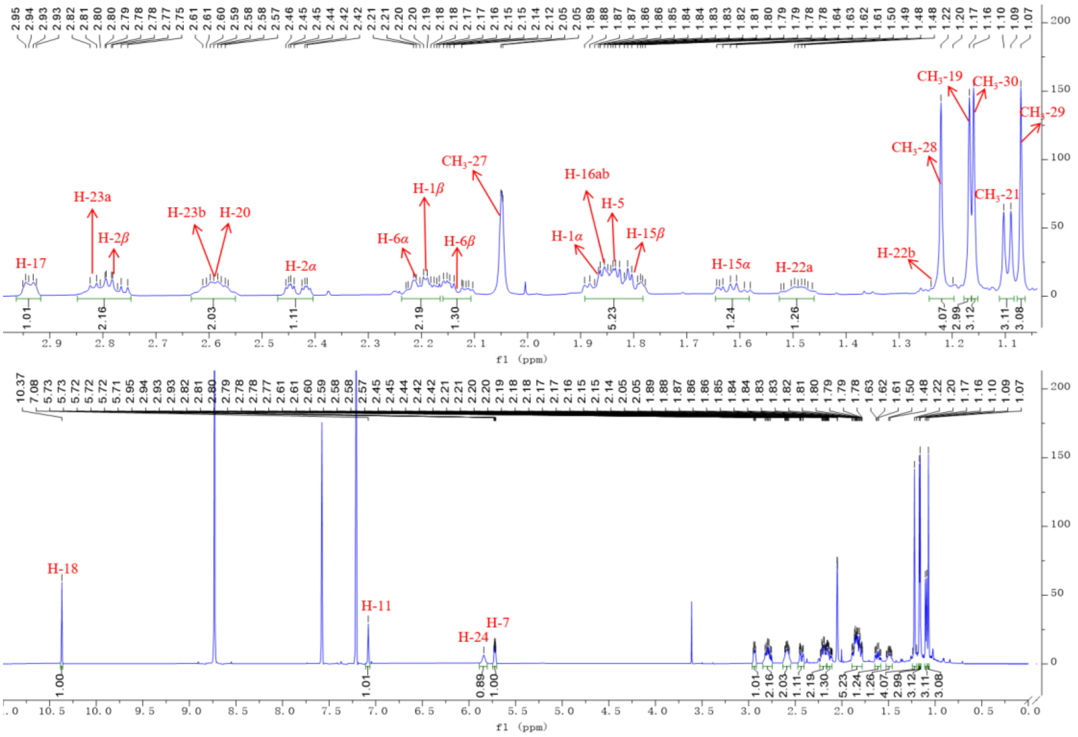

Figure S14 ^{1}H NMR spectrum of kadcoccitane F (**2**) (pyridine-d_5, 500 MHz).

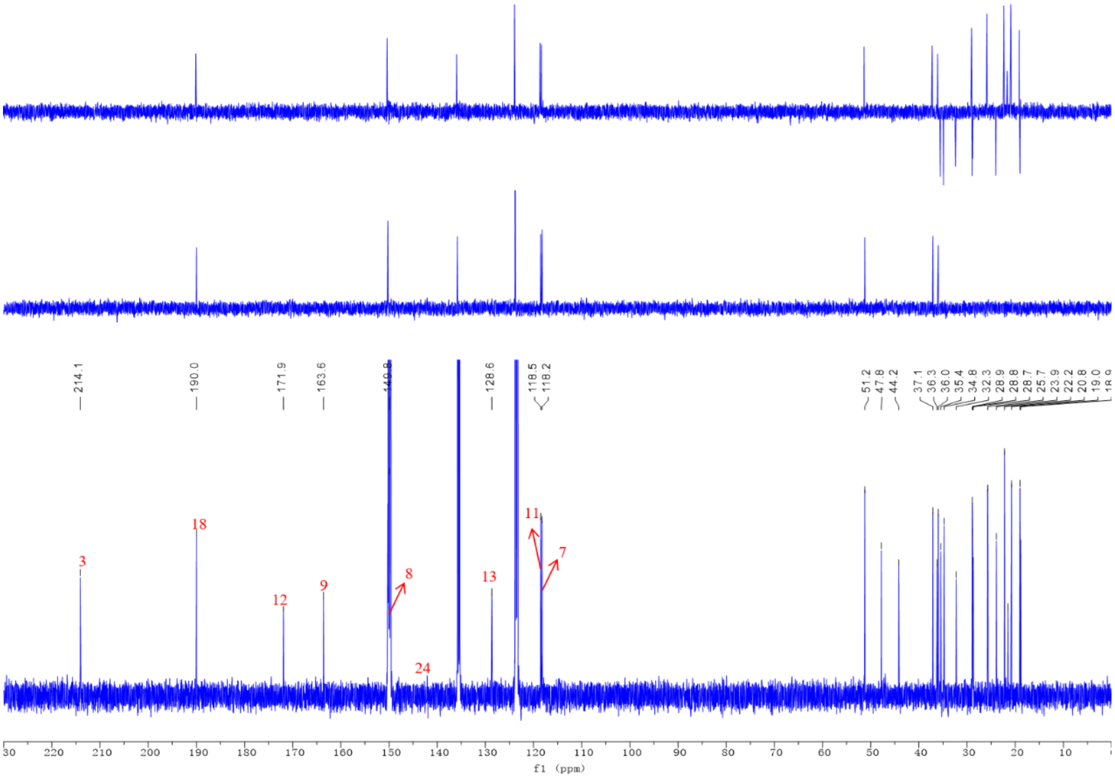

Figure S15 ^{13}C NMR spectrum of kadcoccitane F (**2**) (pyridine-d_5, 125 MHz).

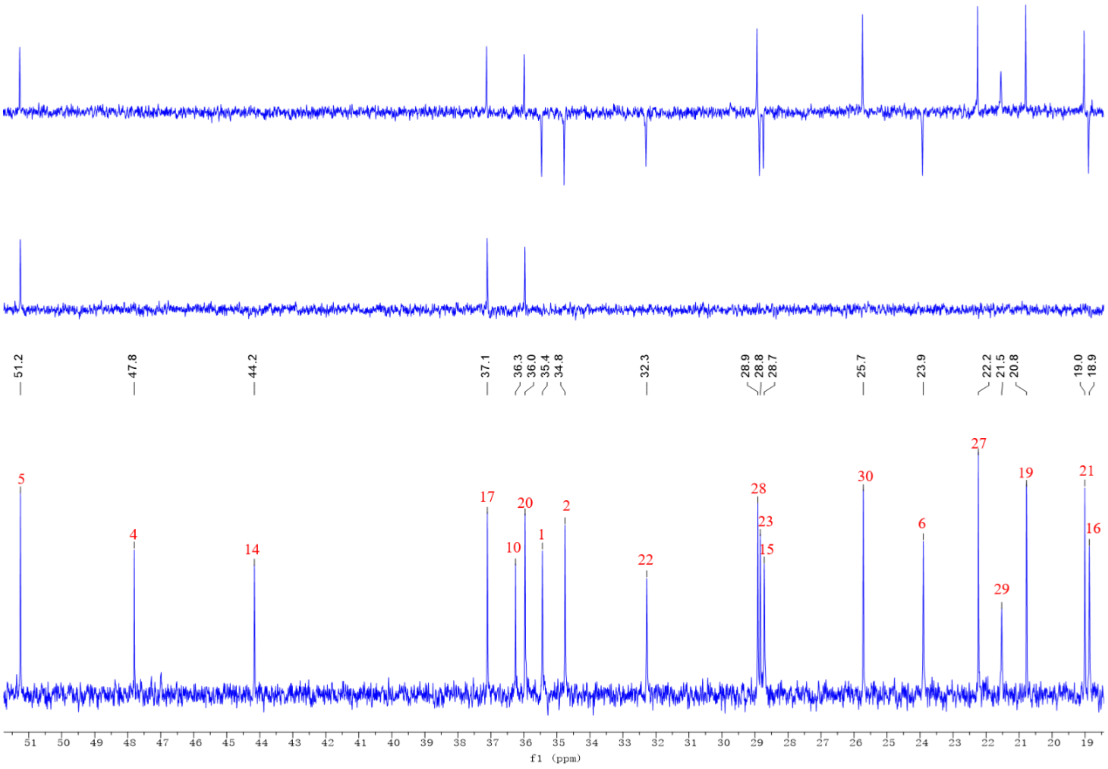

Figure S16 ^{13}C NMR spectrum of kadcoccitane F (**2**) (pyridine-d_5, 125 MHz).

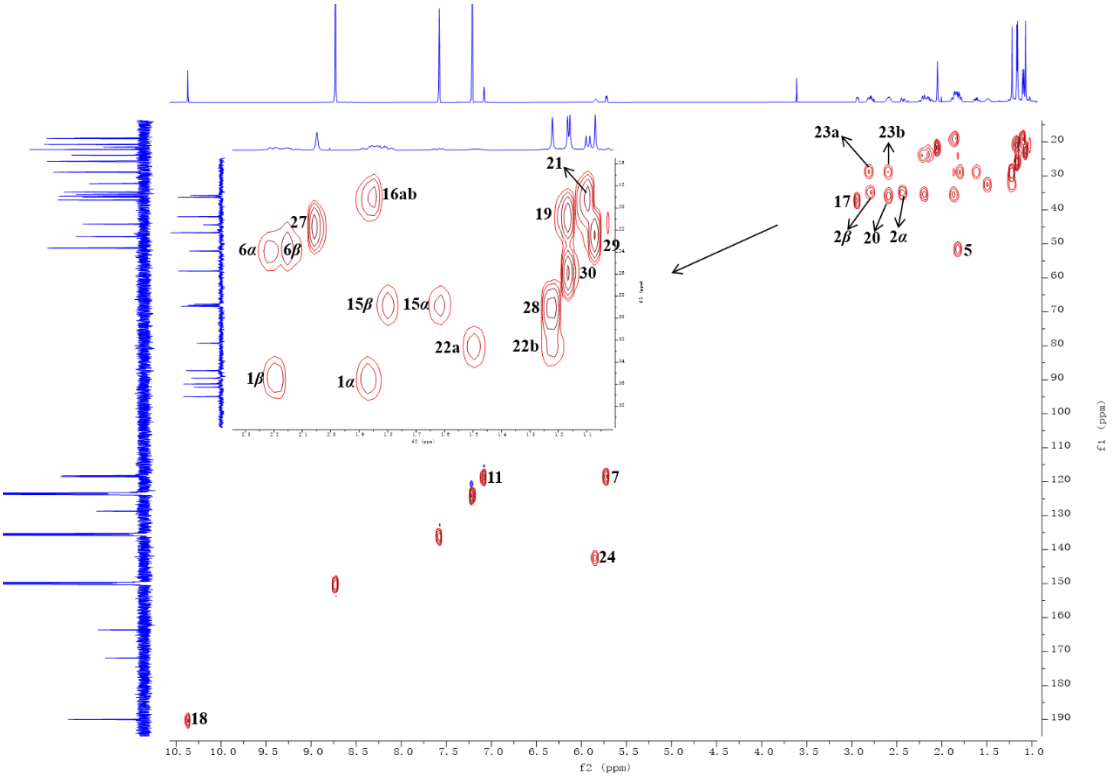

Figure S17 HSQC spectrum of kadcoccitane F (**2**) (pyridine-d_5, 500 MHz).

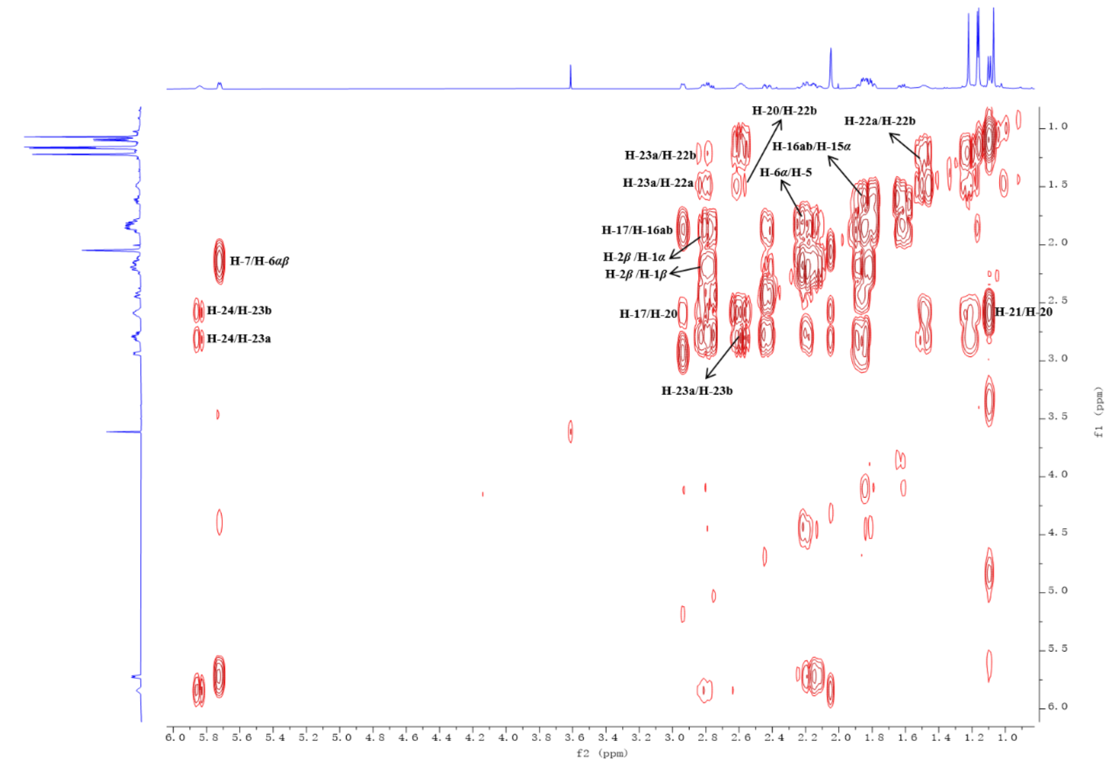

Figure S18 ^{1}H–^{1}H COSY spectrum of kadcoccitane F (**2**) (pyridine-d_5, 500 MHz).

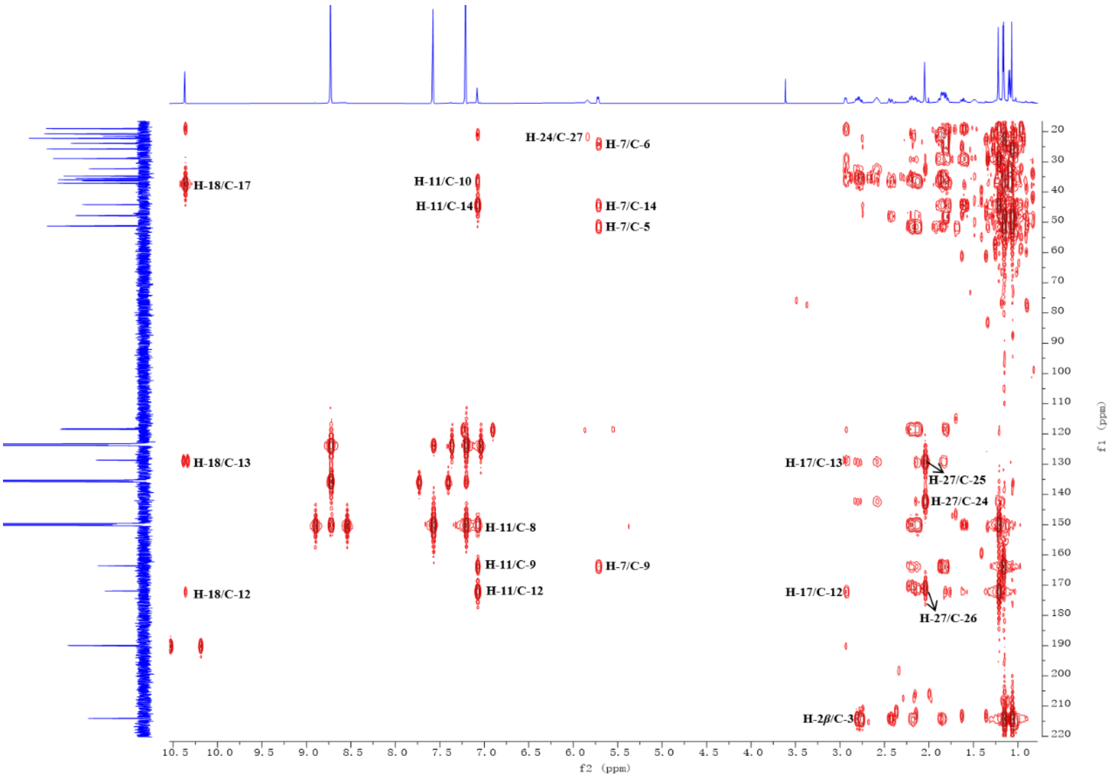

Figure S19 HMBC spectrum of kadcoccitane F (**2**) (pyridine-d_5, 500 MHz).

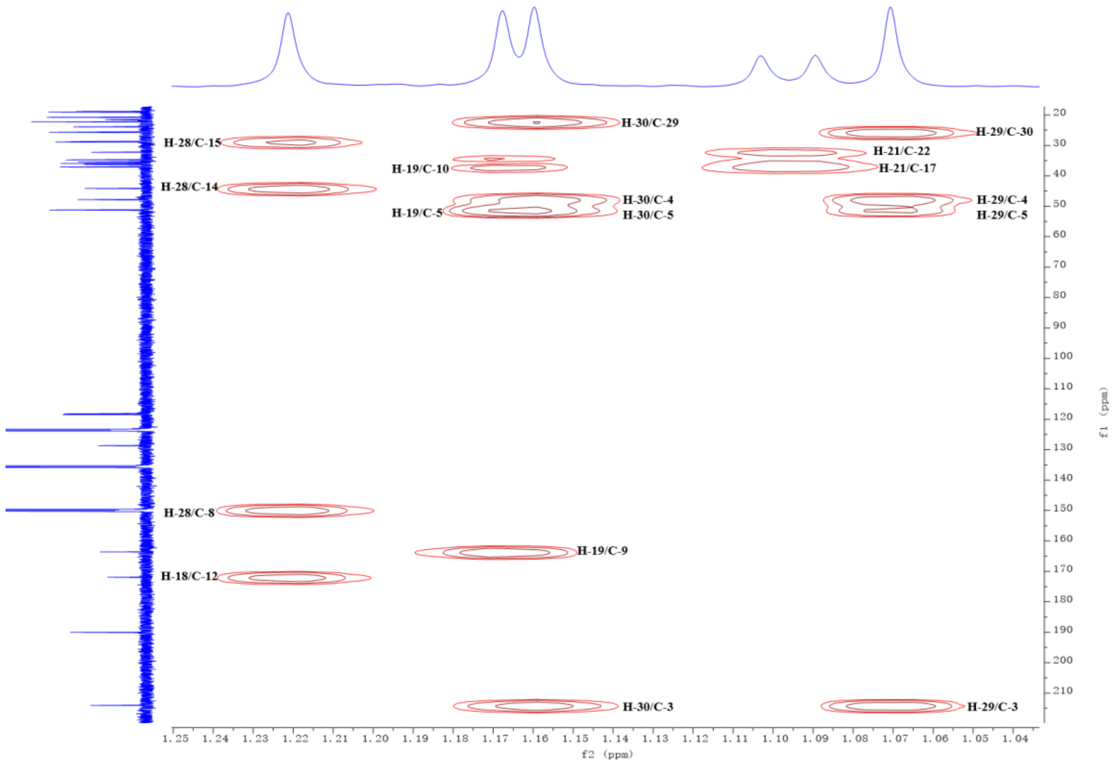

Figure S20 Locally magnified HMBC spectrum of kadcoccitane F (**2**) (pyridine-d_5, 500 MHz).

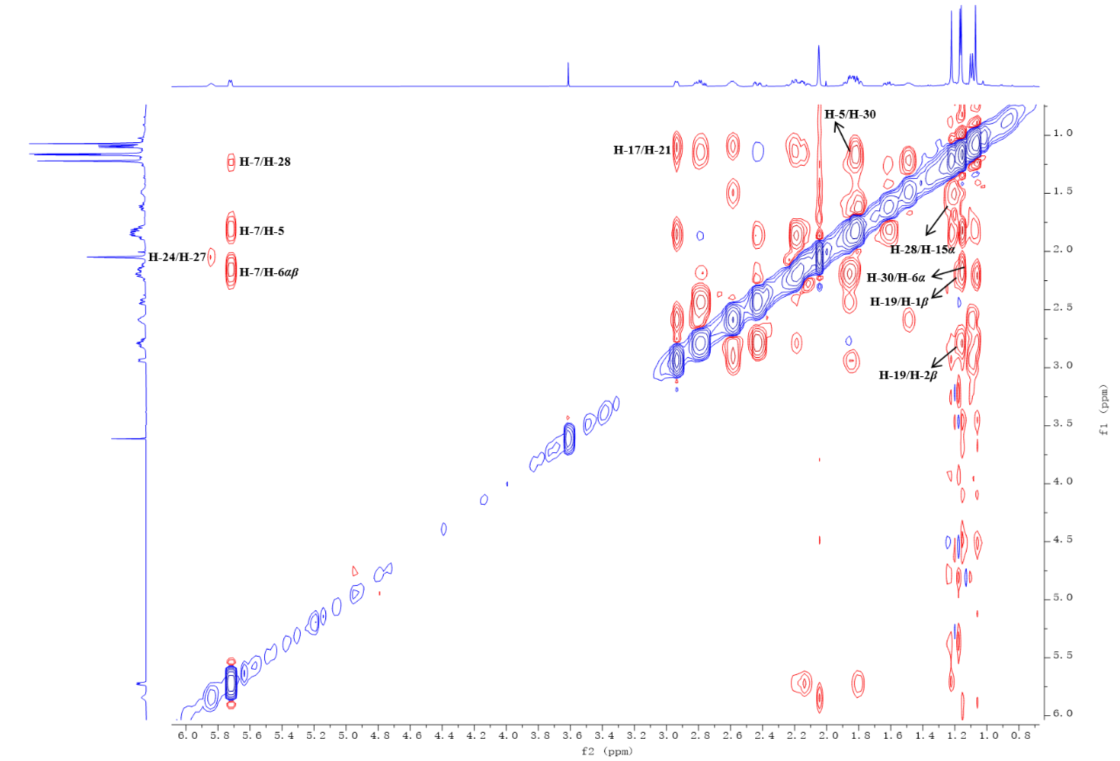

Figure S21 ROESY spectrum of kadcoccitane F (**2**) (pyridine-d_5, 500 MHz).

Qualitative Analysis Report

**Data Filename**	szqq-43M.d	**Sample Name**	szqq-43M
**Sample Type**	Sample	**Position**	P1-B4
**Instrument Name**	Instrument 1	**User Name**	
**Acq Method**	s-.m	**Acquired Time**	4/19/2022 4:32:14 PM
**IRM Calibration Status**	Success	**DA Method**	PCDL.m
**Comment**			

**Sample Group**		**Info.**
**Acquisition SW Version**	6200 series TOF/6500 series Q-TOF B.05.01 (B5125.2)	

User Spectra

Fragmentor Voltage	Collision Energy	Ionization Mode
135	0	ESI

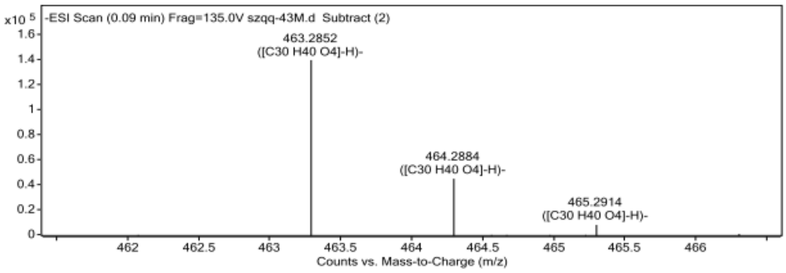

Peak List

m/z	z	Abund	Formula	Ion
463.2852	1	139701.55	C30 H40 O4	(M-H)-
464.2884	1	45564.18	C30 H40 O4	(M-H)-
465.2914	1	9072.67	C30 H40 O4	(M-H)-
479.2797	1	10974.15		
525.3212	1	7696.31		
531.2732	1	13079.04		
532.2747	1	5576.21		
577.2775	1	17325.41		
599.2611	1	7885.24		
1384.2873	1	8967.4		

Formula Calculator Element Limits

Element	Min	Max
C	3	60
H	0	120
O	0	30

Formula Calculator Results

Formula	CalculatedMass	CalculatedMz	Mz	Diff. (mDa)	Diff. (ppm)	DBE
C30 H40 O4	464.2927	463.2854	463.2852	0.20	0.43	11.0000

--- End Of Report ---

Figure S22 HRESIMS spectrum of kadcoccitane F (**2**).

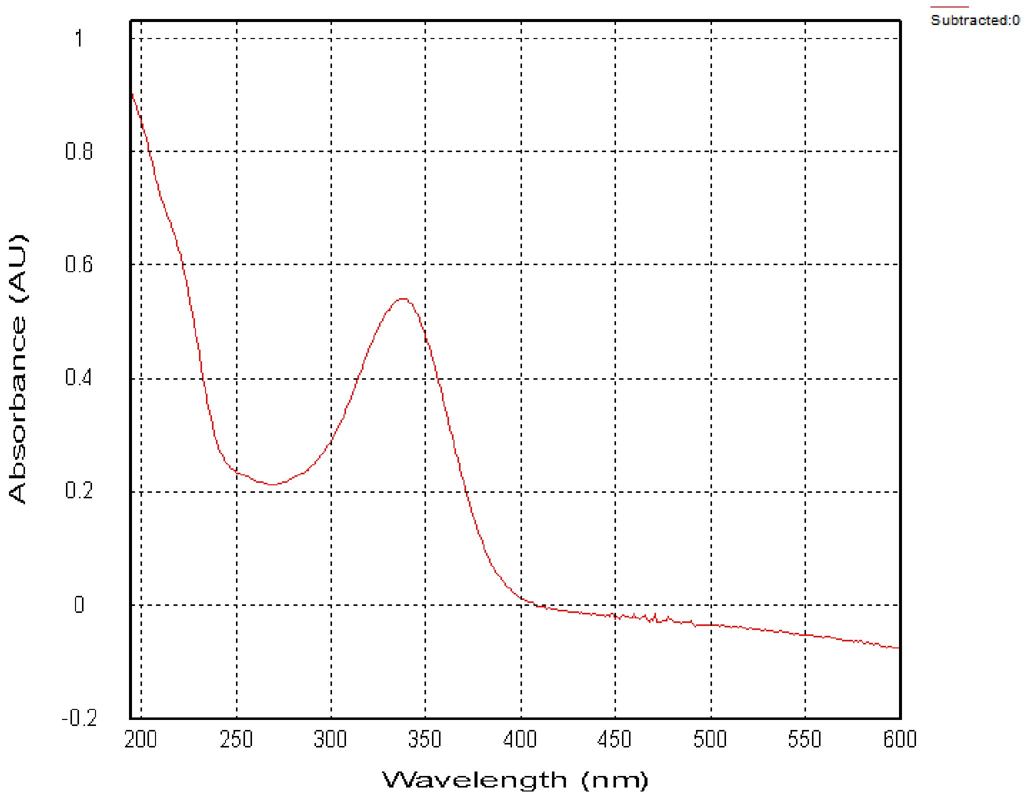

Figure S23 UV spectrum of kadcoccitane F (**2**).

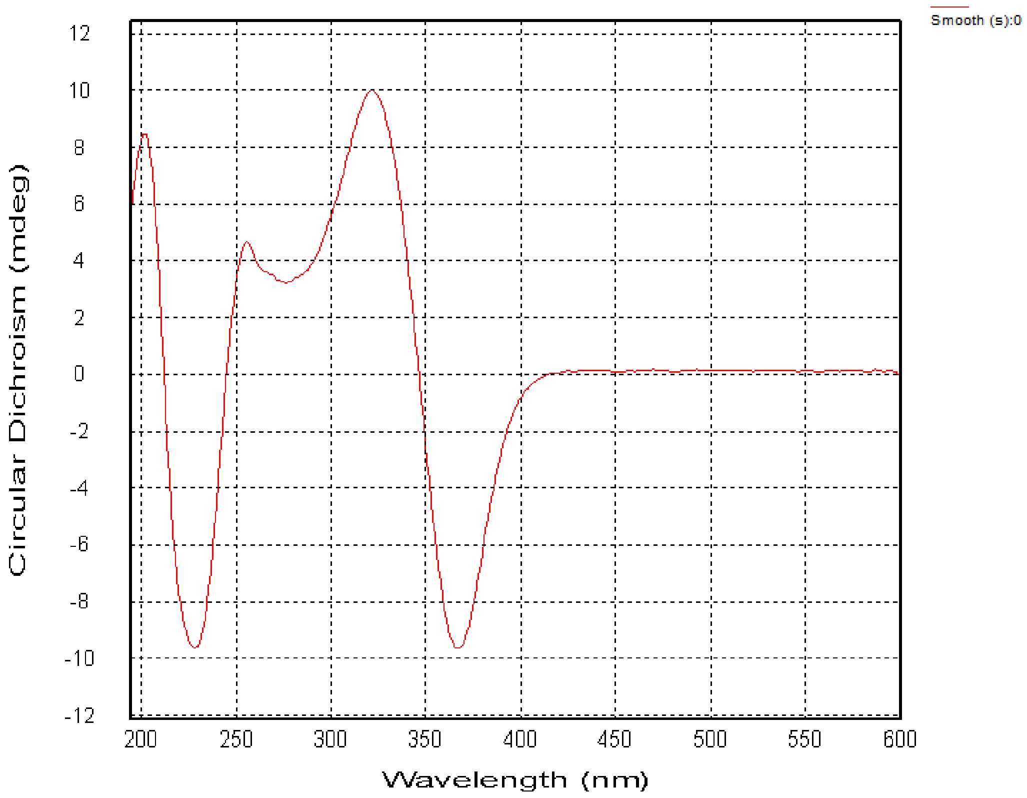

Figure S24 ECD spectrum of kadcoccitane F (**2**).

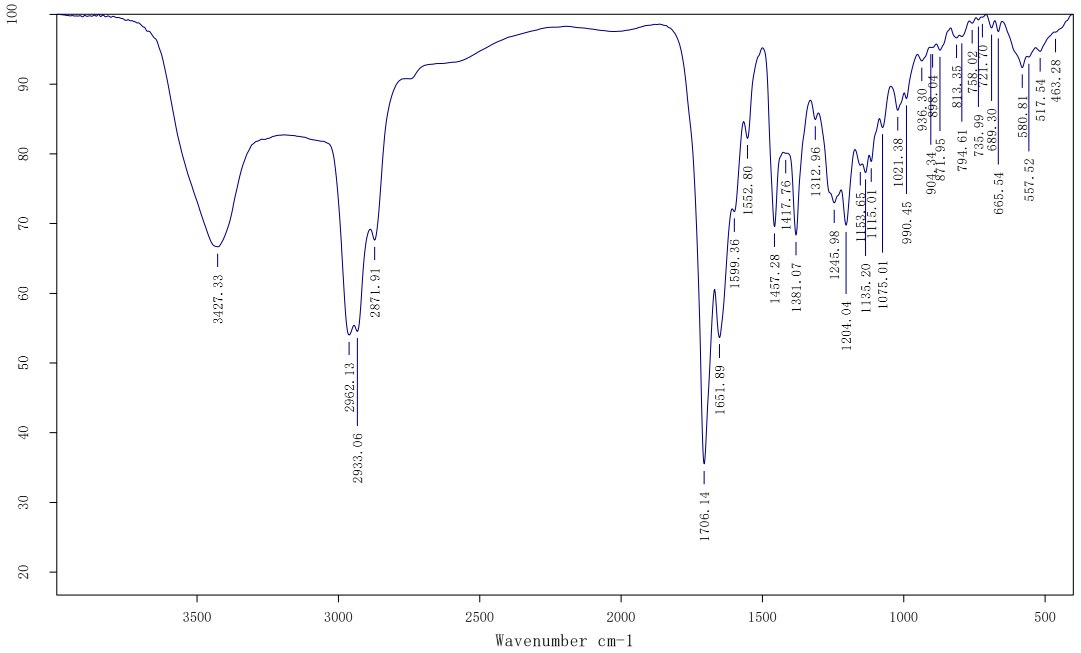

Sample Name: szqq-43M	Resolution: 4	Beamsplitter Setting: KBr
Sample Form: KBr	Aperture Setting: 6 mm	Source Setting: MIR
Path of File: E:\data	Number of Background Scans: 16	Instrument Type: BRUKER VERTEX 70
Date of Measurement: 2022/5/12	Number of Sample Scans: 16	Soft Version: OPUS8.1

Figure S25 IR spectrum of kadcoccitane F (**2**).

Rudolph Research Analytical

This sample was measured on an Autopol VI, Serial #91058
Manufactured by Rudolph Research Analytical, Hackettstown, NJ, USA.

Measurement Date : Wednesday, 11-MAY-2022

Set Temperature : OFF

Time Delay : Disabled

Delay between Measurement : Disabled

n	Average	Std.Dev.	% RSD	Maximum	Minimum
5	-51.09	0.56	-1.09	-50.22	-51.63

S.No	Sample ID	Time	Result	Scale	OR °Arc	WLG.nm	Lg.mm	Conc.g/100ml	Temp.
1	SZQQ-43M	11:53:11 AM	-50.22	SR	-0.0462	589	100.00	0.092	25.2
2	SZQQ-43M	11:53:19 AM	-50.87	SR	-0.0468	589	100.00	0.092	25.2
3	SZQQ-43M	11:53:27 AM	-51.41	SR	-0.0473	589	100.00	0.092	25.1
4	SZQQ-43M	11:53:35 AM	-51.63	SR	-0.0475	589	100.00	0.092	25.1
5	SZQQ-43M	11:53:44 AM	-51.30	SR	-0.0472	589	100.00	0.092	25.1

Figure S26 OR of kadcoccitane F (**2**).

6. NMR, MS, UV, ECD, IR spectra, and OR of kadcoccitane G (3)

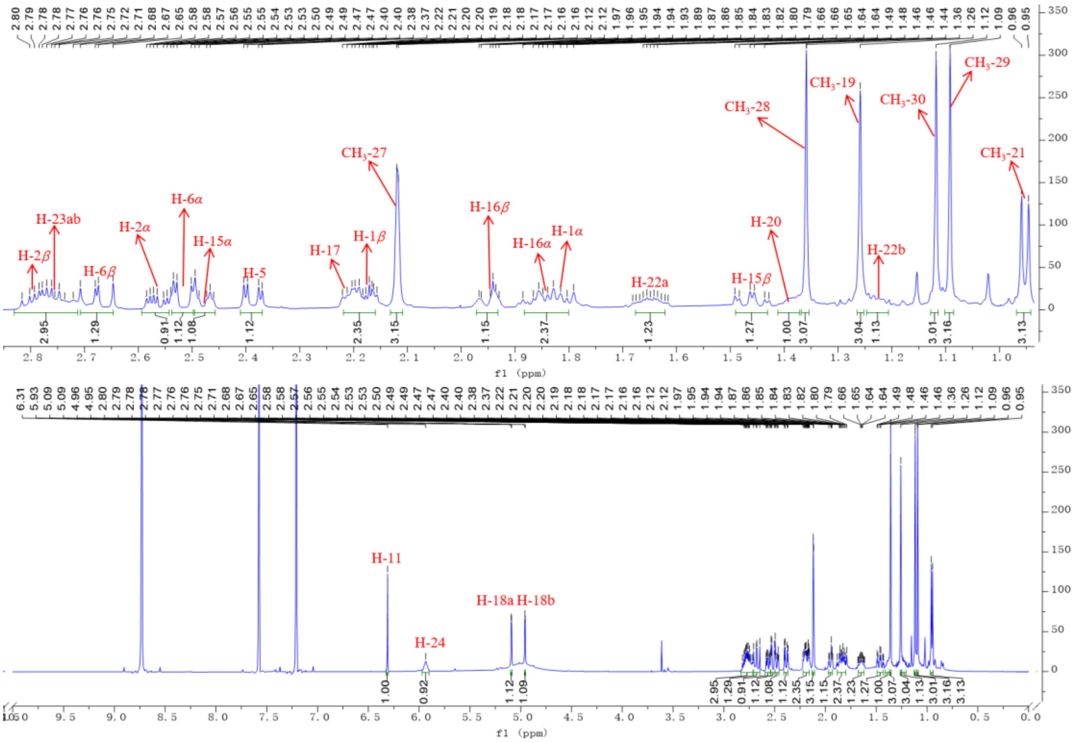

Figure S27 ^{1}H NMR spectrum of kadcoccitane G (**3**) (pyridine-d_5, 500 MHz).

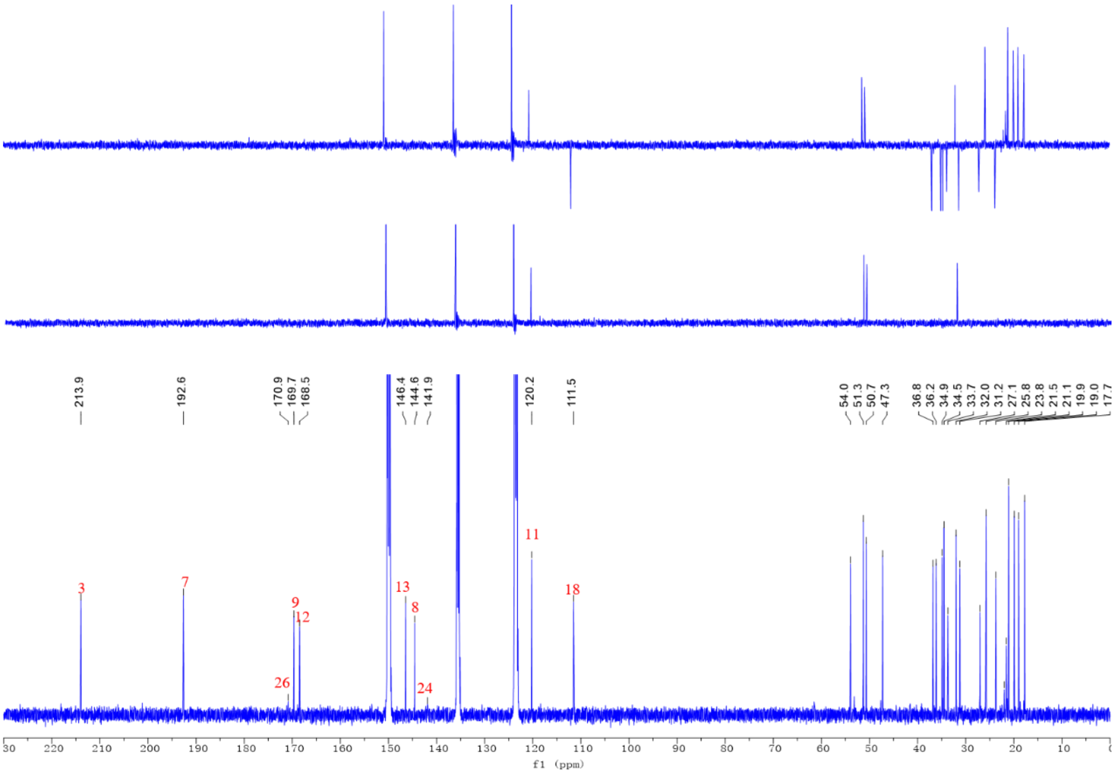

Figure S28 ^{13}C NMR spectrum of kadcoccitane G (**3**) (pyridine-d_5, 125 MHz).

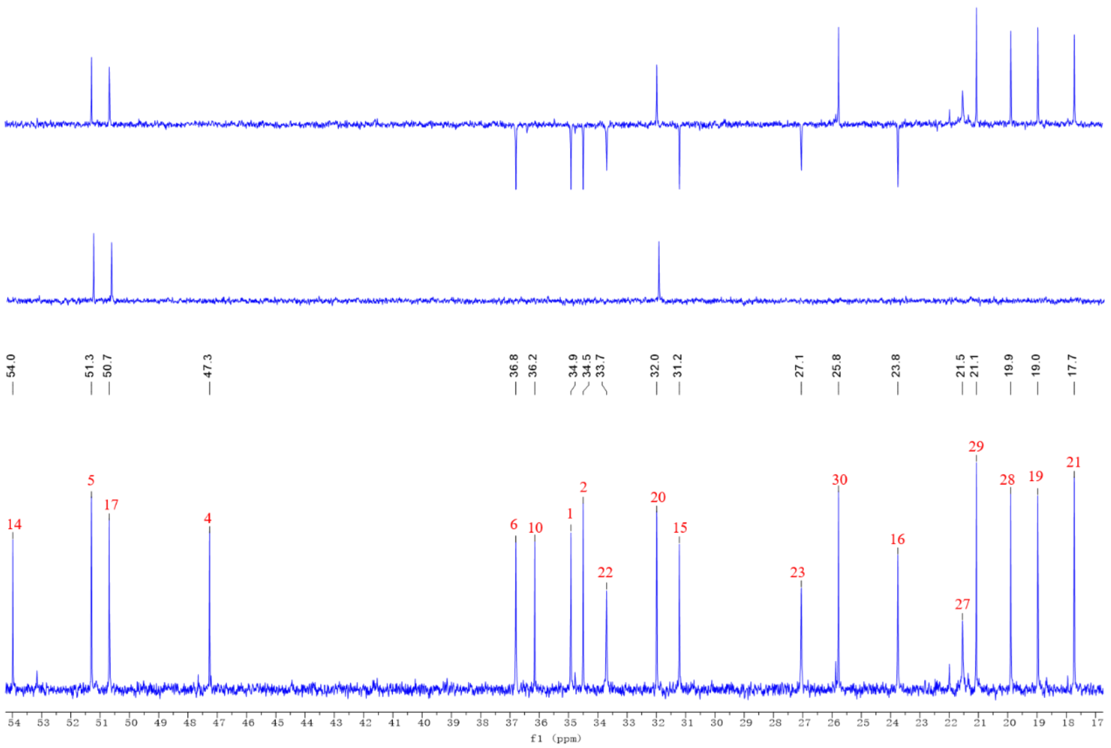

Figure S29 ^{13}C NMR spectrum of kadcoccitane G (**3**) (pyridine-d_5, 125 MHz).

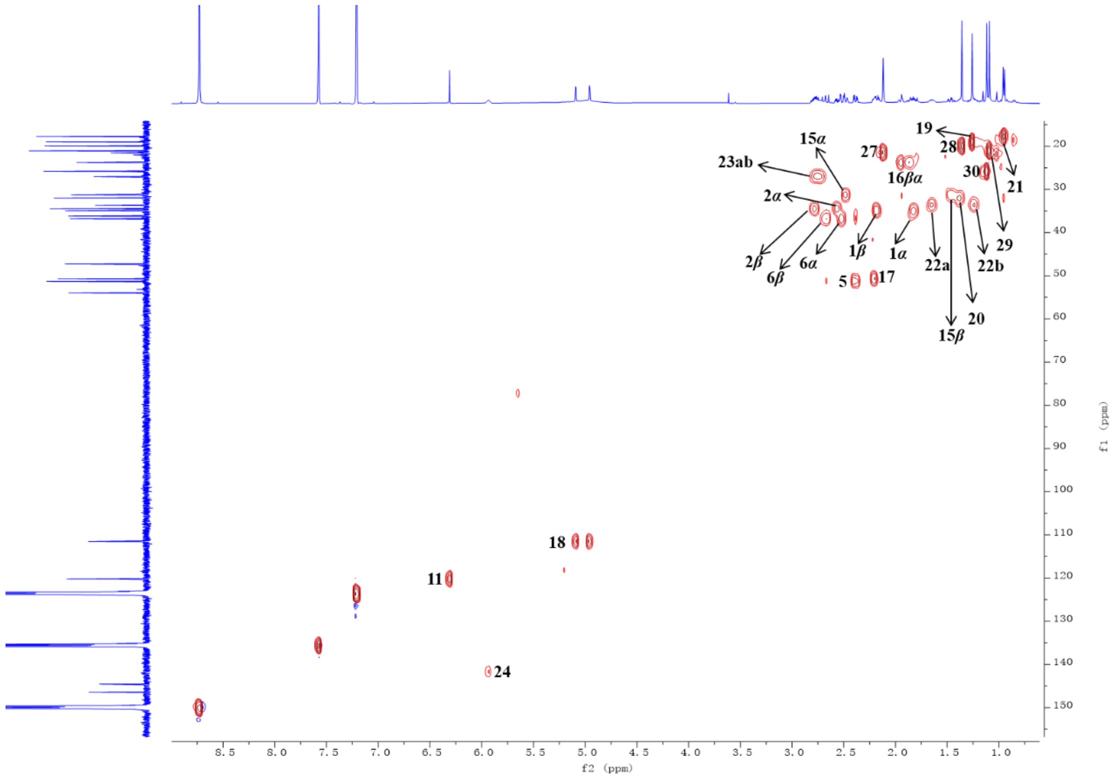

Figure S30 HSQC spectrum of kadcoccitane G (**3**) (pyridine-d_5, 500 MHz).

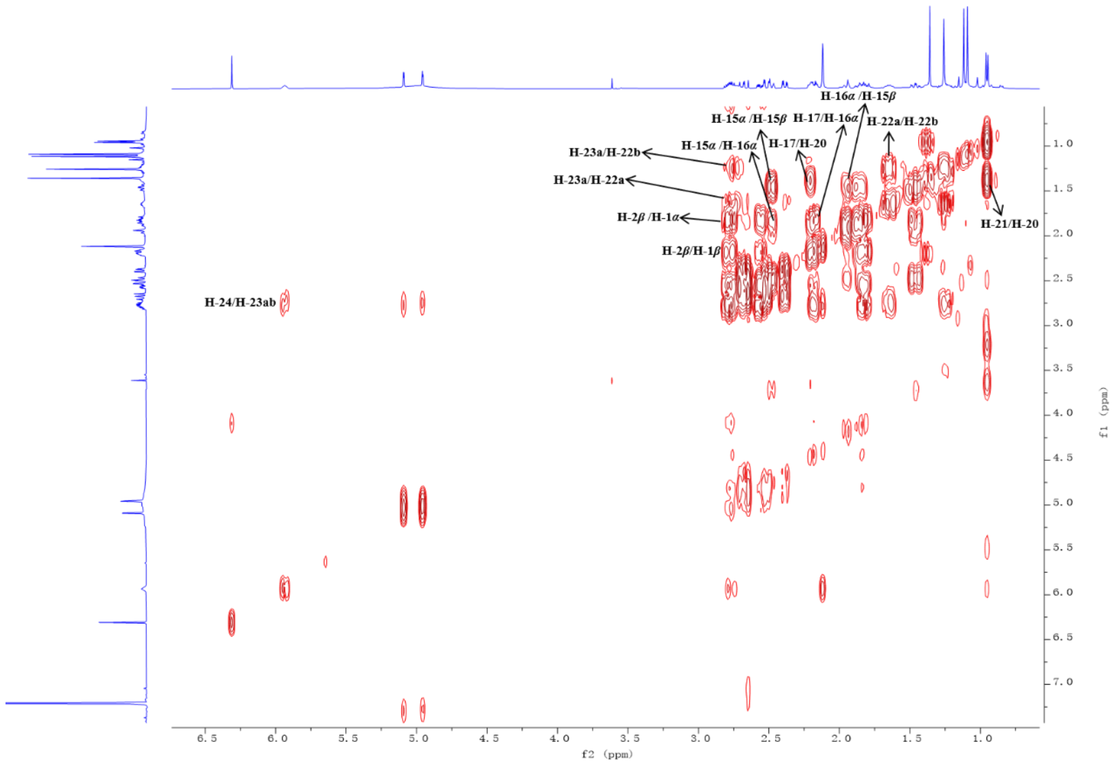

Figure S31 ^{1}H–^{1}H COSY spectrum of kadcoccitane G (**3**) (pyridine-d_5, 500 MHz).

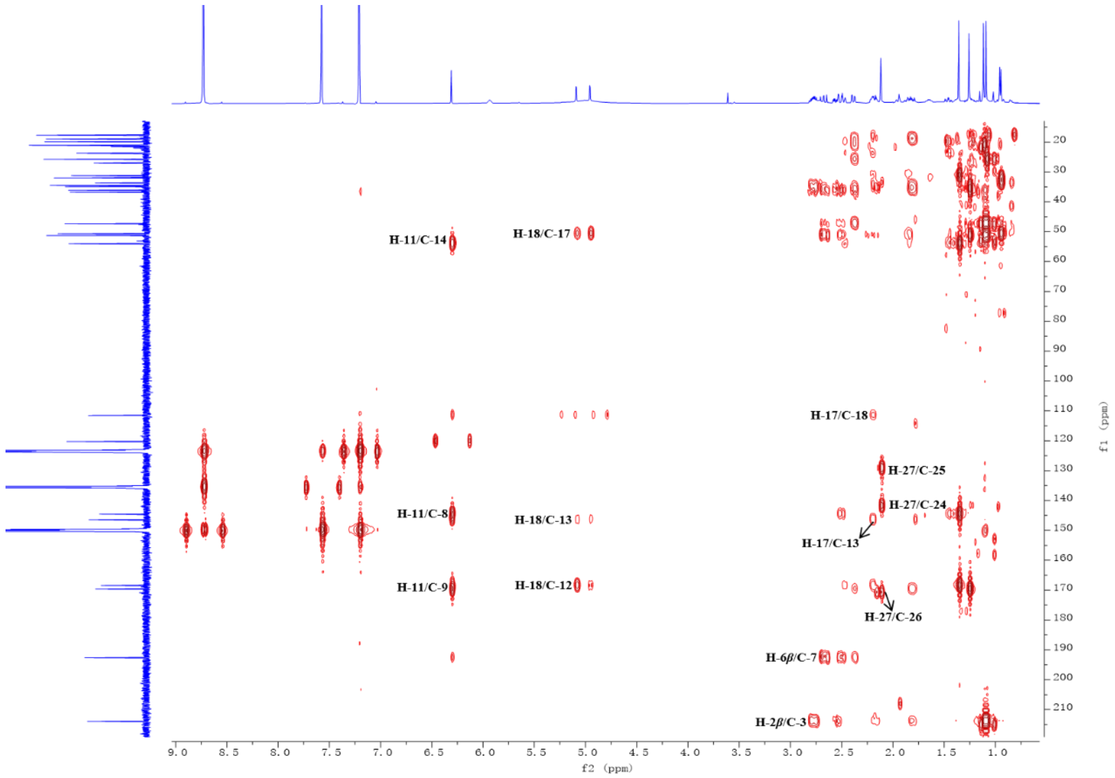

Figure S32 HMBC spectrum of kadcoccitane G (**3**) (pyridine-d_5, 500 MHz).

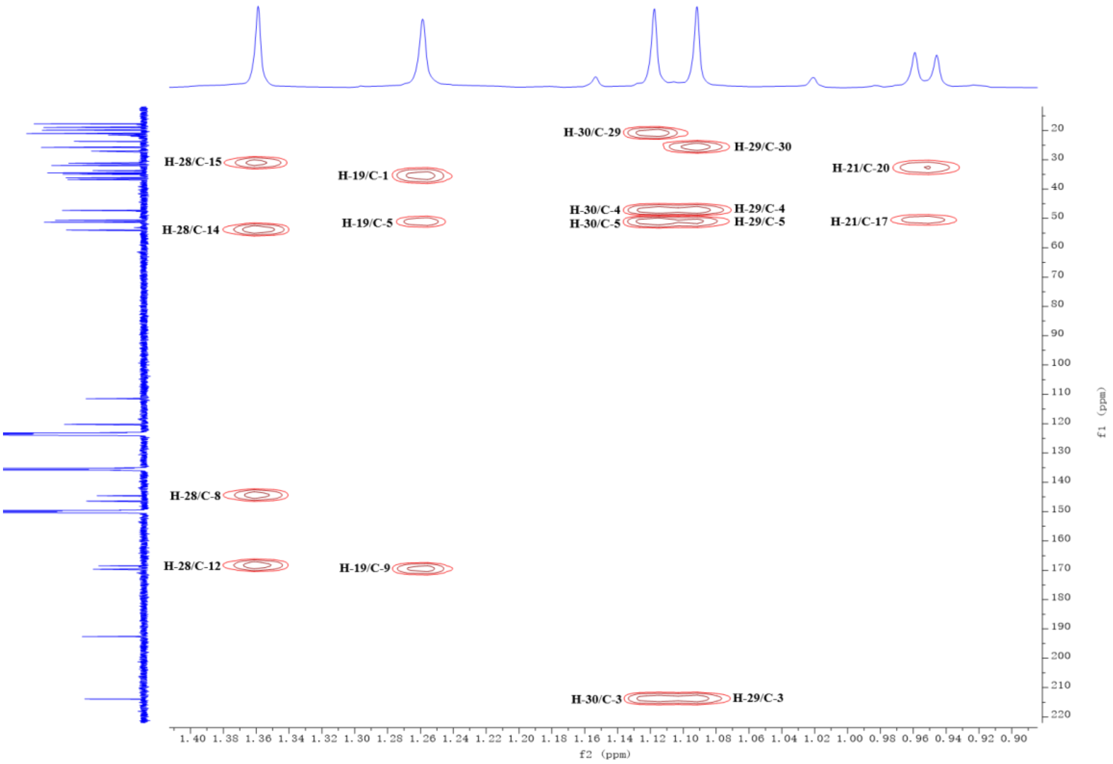

Figure S33 Locally magnified HMBC spectrum of kadcoccitane G (**3**) (pyridine-d_5, 500 MHz).

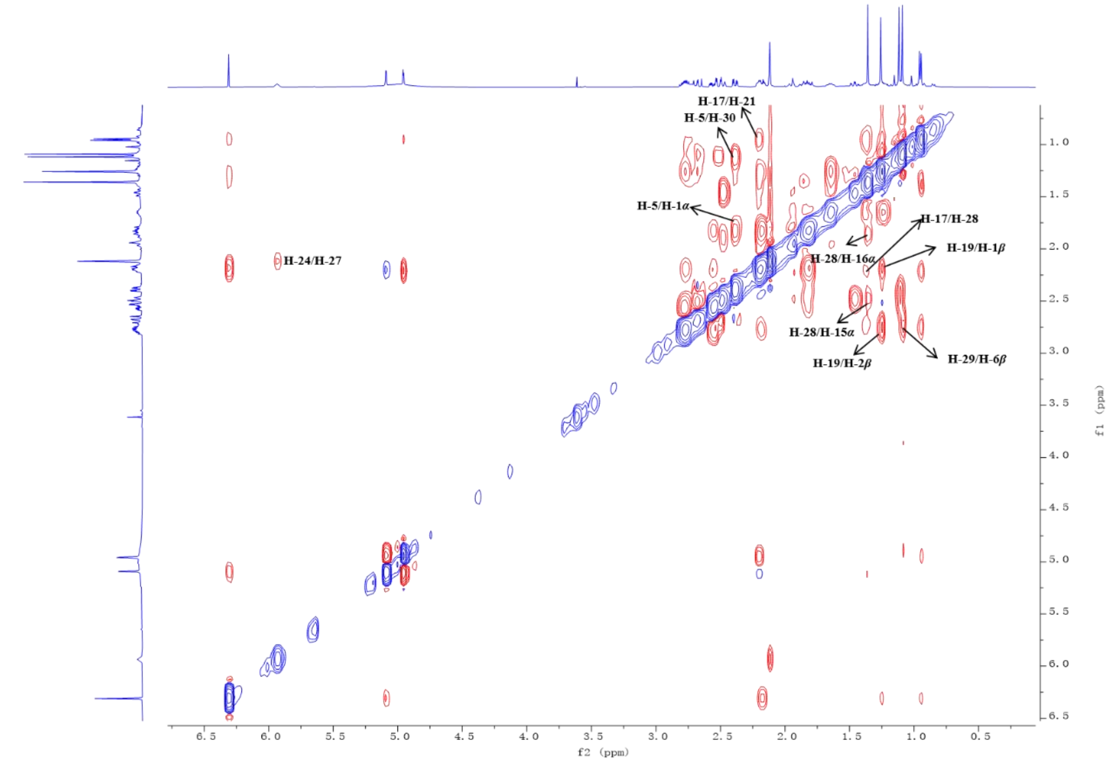

Figure S34 ROESY spectrum of kadcoccitane G (**3**) (pyridine-d_5, 500 MHz).

Qualitative Analysis Report

**Data Filename**	szqq-51Y.d	**Sample Name**	szqq-51Y
**Sample Type**	Sample	**Position**	P1-C4
**Instrument Name**	Instrument 1	**User Name**	
**Acq Method**	s-.m	**Acquired Time**	4/29/2022 3:15:52 PM
**IRM Calibration Status**	Success	**DA Method**	PCDL.m
**Comment**			

Sample Group **Info.**
Acquisition SW Version 6200 series TOF/6500 series Q-TOF B.05.01 (B5125.2)

User Spectra

Fragmentor Voltage	Collision Energy	Ionization Mode
135	0	ESI

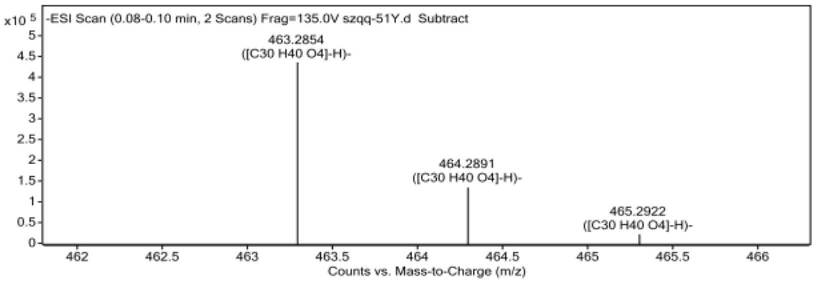

Peak List

m/z	z	Abund	Formula	Ion
463.2854	1	437028.34	C30 H40 O4	(M-H)-
464.2891	1	138471.88	C30 H40 O4	(M-H)-
465.2922	1	24075.64	C30 H40 O4	(M-H)-
499.2631	1	27613.39		
531.2732	1	36037.02		
557.3484	1	29850.09		
927.5794	1	100882.27		
928.5825	1	66400.07		
929.5858	1	23819.27		
949.5628	1	26394.46		

Formula Calculator Element Limits

Element	Min	Max
C	3	60
H	0	120
O	0	30

Formula Calculator Results

Formula	CalculatedMass	CalculatedMz	Mz	Diff. (mDa)	Diff. (ppm)	DBE
C30 H40 O4	464.2927	463.2854	463.2854	0.00	0.00	11.0000

--- End Of Report ---

Figure S35 HRESIMS spectrum of kadcoccitane G (**3**).

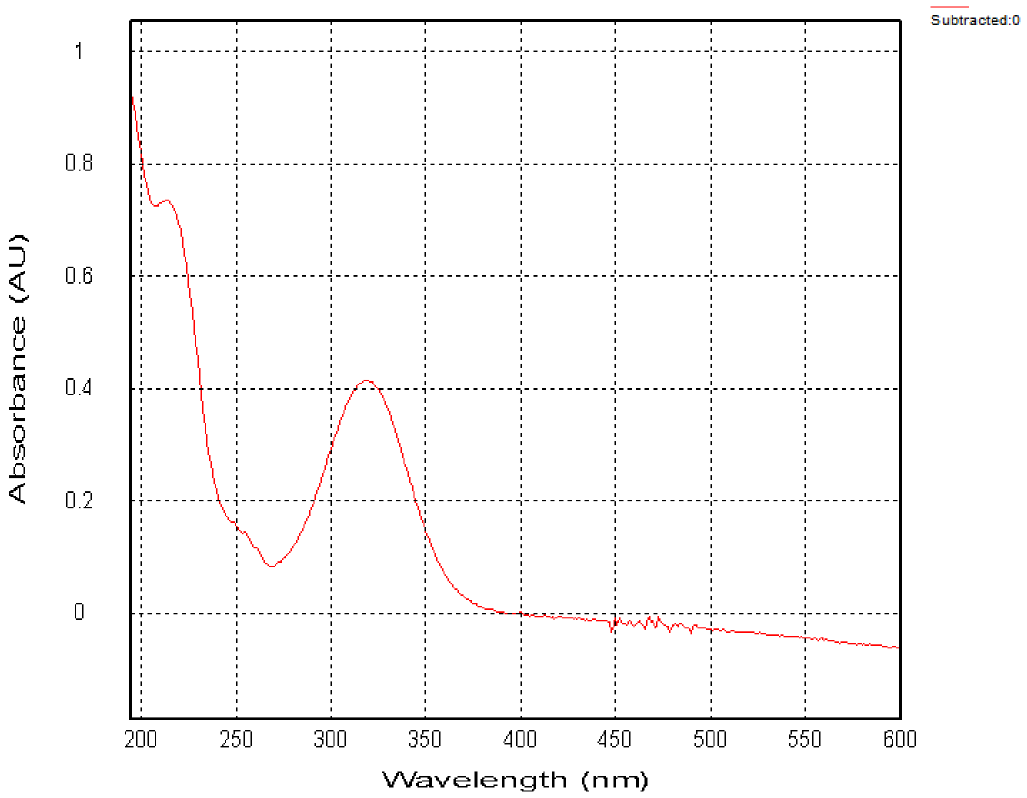

Figure S36 UV spectrum of kadcoccitane G (**3**).

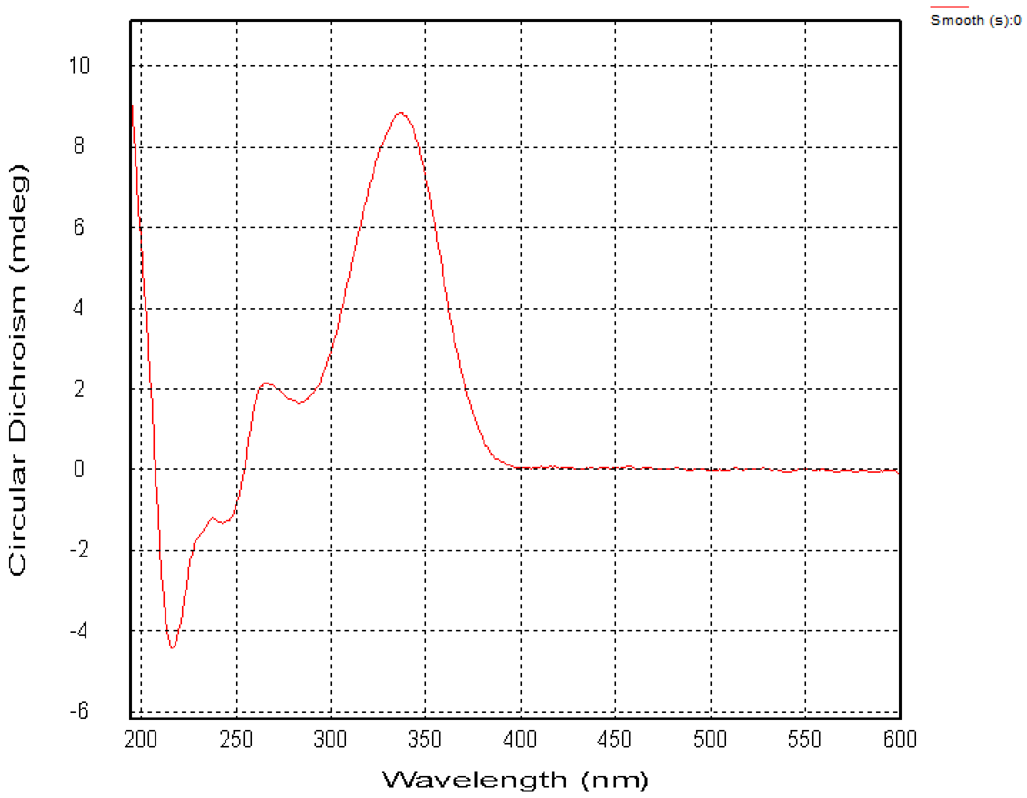

Figure S37 ECD spectrum of kadcoccitane G (**3**).

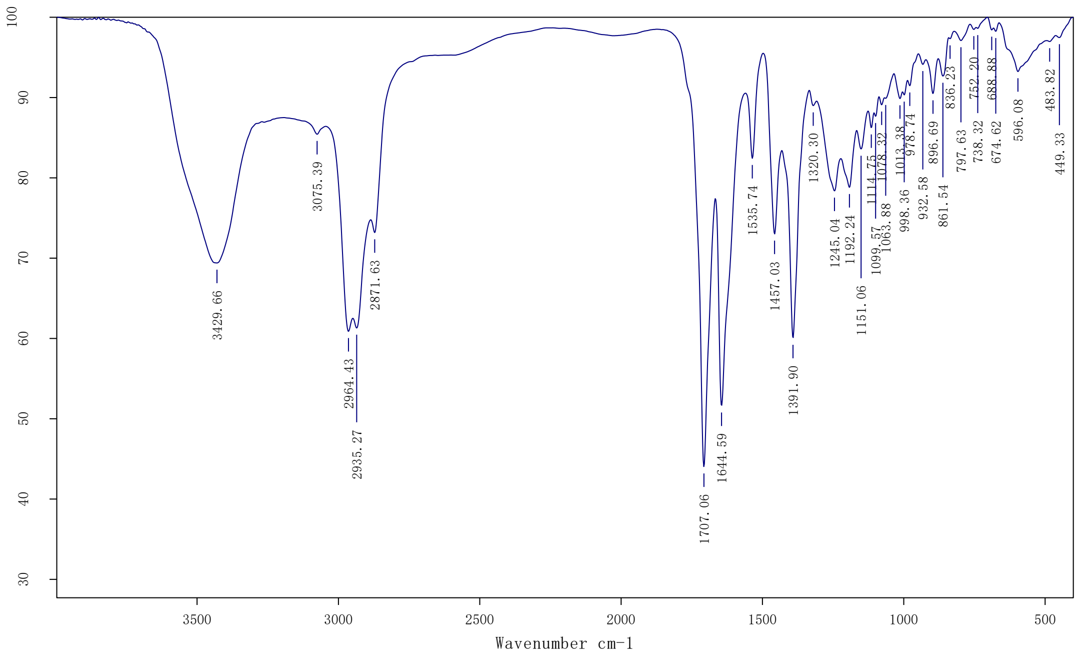

Sample Name: szqq-51Y	Resolution: 4	Beamsplitter Setting: KBr
Sample Form: KBr	Aperture Setting: 6 mm	Source Setting: MIR
Path of File: E:\data	Number of Background Scans: 16	Instrument Type: BRUKER VERTEX 70
Date of Measurement: 2022/5/12	Number of Sample Scans: 16	Soft Version: OPUS8.1

Figure S38 IR spectrum of kadcoccitane G (**3**).

Rudolph Research Analytical

This sample was measured on an Autopol VI, Serial #91058
Manufactured by Rudolph Research Analytical, Hackettstown, NJ, USA.

Measurement Date : Monday, 16-MAY-2022

Set Temperature : OFF

Time Delay : Disabled

Delay between Measurement : Disabled

n	Average	Std.Dev.	% RSD	Maximum	Minimum
5	103.68	0.24	0.23	103.84	103.26

S.No	Sample ID	Time	Result	Scale	OR °Arc	WLG.nm	Lg.mm	Conc.g/100ml	Temp.
1	SZQQ-51Y	09:13:28 AM	103.84	SR	0.0893	589	100.00	0.086	19.3
2	SZQQ-51Y	09:13:37 AM	103.26	SR	0.0888	589	100.00	0.086	19.3
3	SZQQ-51Y	09:13:45 AM	103.72	SR	0.0892	589	100.00	0.086	19.3
4	SZQQ-51Y	09:13:53 AM	103.72	SR	0.0892	589	100.00	0.086	19.3
5	SZQQ-51Y	09:14:01 AM	103.84	SR	0.0893	589	100.00	0.086	19.3

Figure S39 OR of kadcoccitane G (**3**).

7. NMR, MS, UV, ECD, IR spectra, and OR of kadcoccitane H (4)

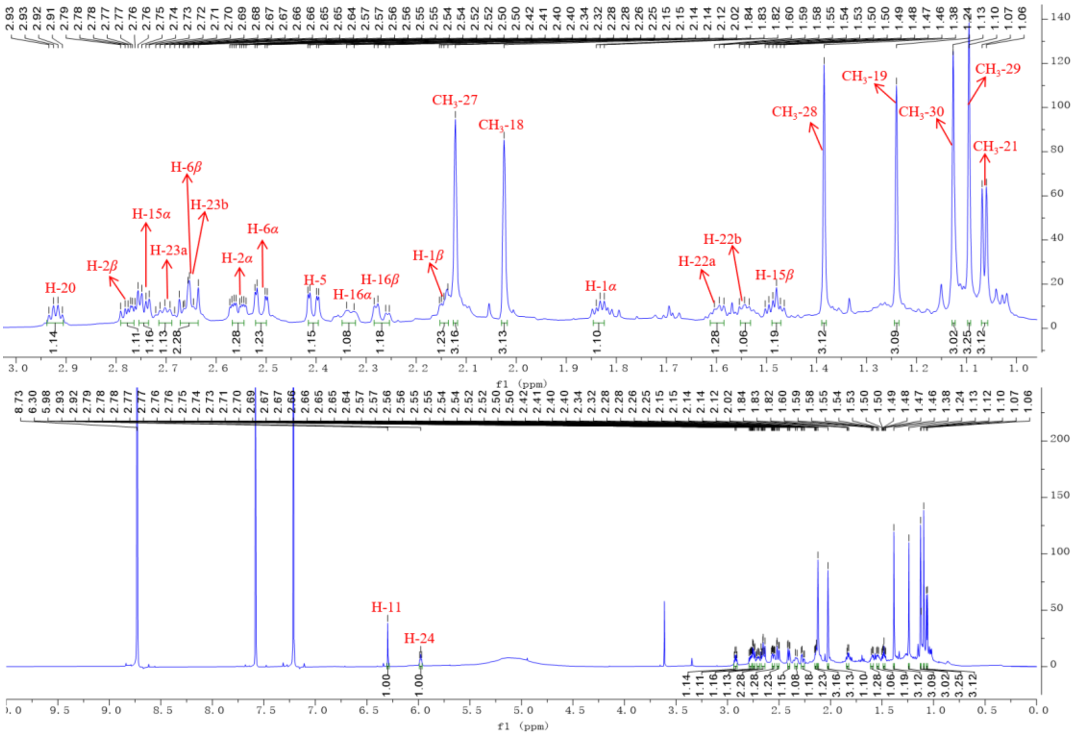

Figure S40 ^{1}H NMR spectrum of kadcoccitane H (**4**) (pyridine-d_5, 800 MHz).

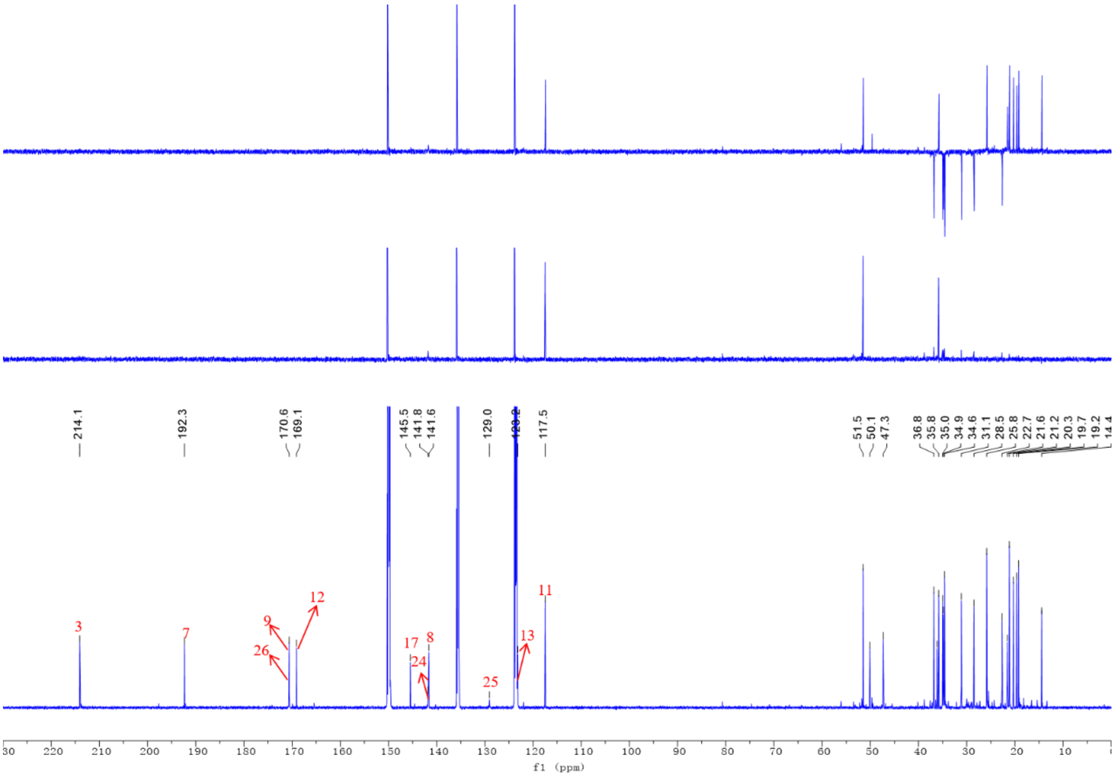

Figure S41 ^{13}C NMR spectrum of kadcoccitane H (**4**) (pyridine-d_5, 200 MHz).

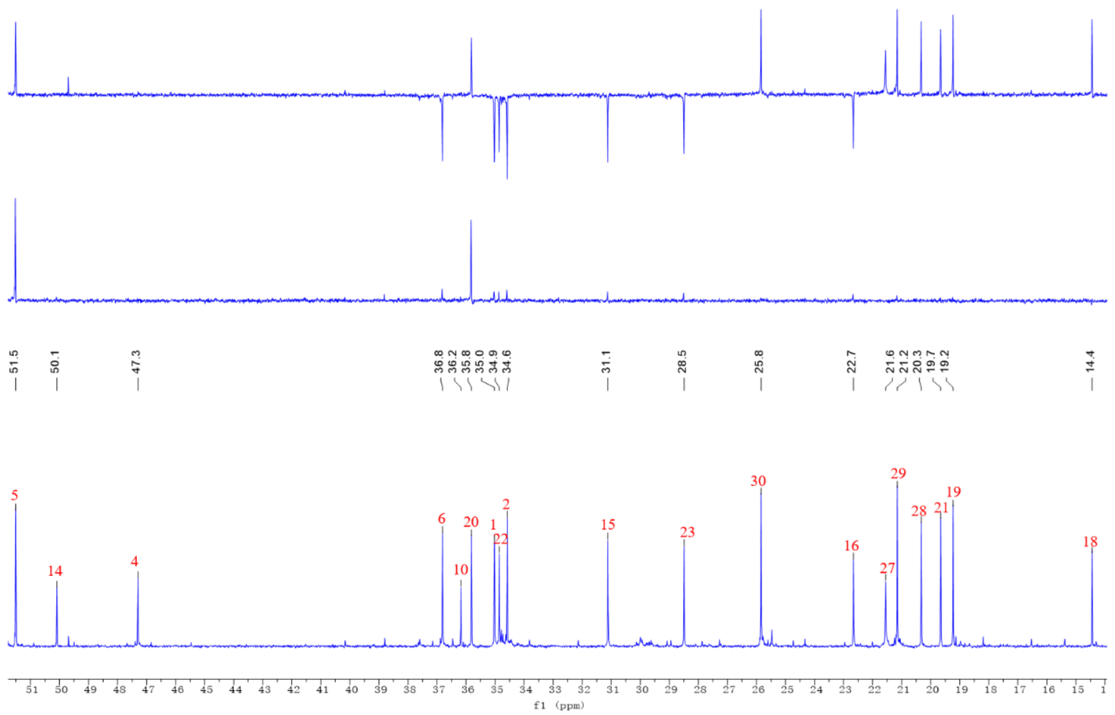

Figure S42 ^{13}C NMR spectrum of kadcoccitane H (**4**) (pyridine-d_5, 200 MHz).

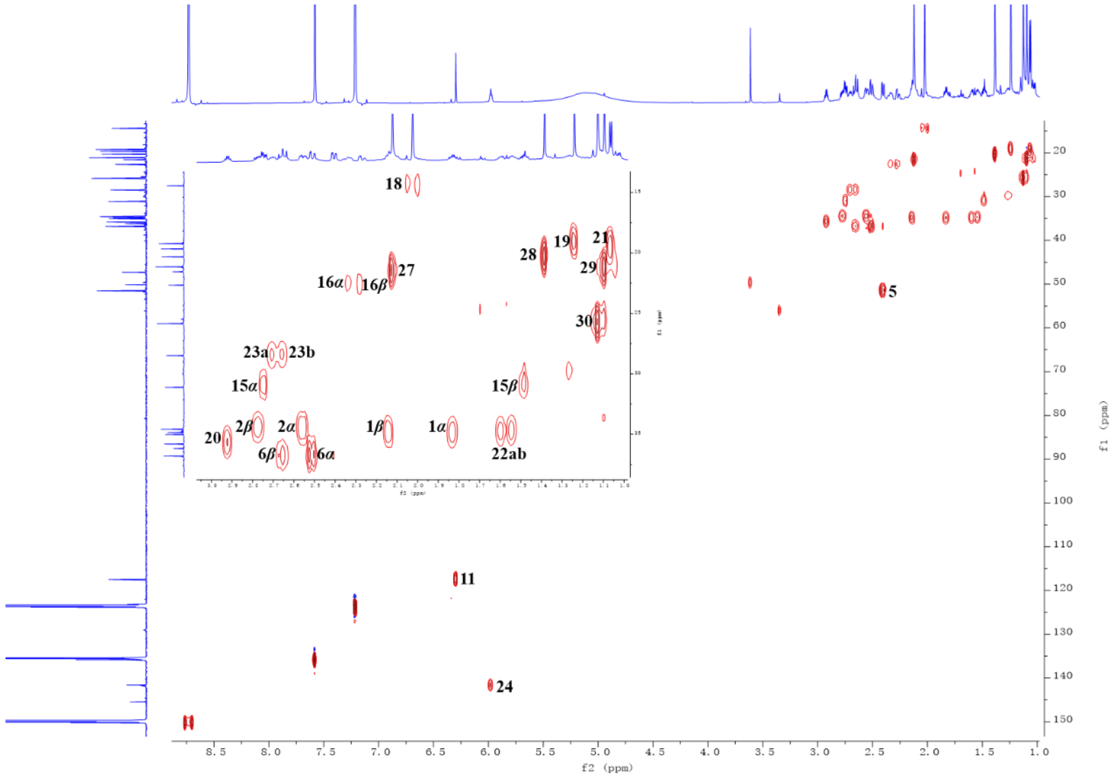

Figure S43 HSQC spectrum of kadcoccitane H (**4**) (pyridine-d_5, 800 MHz).

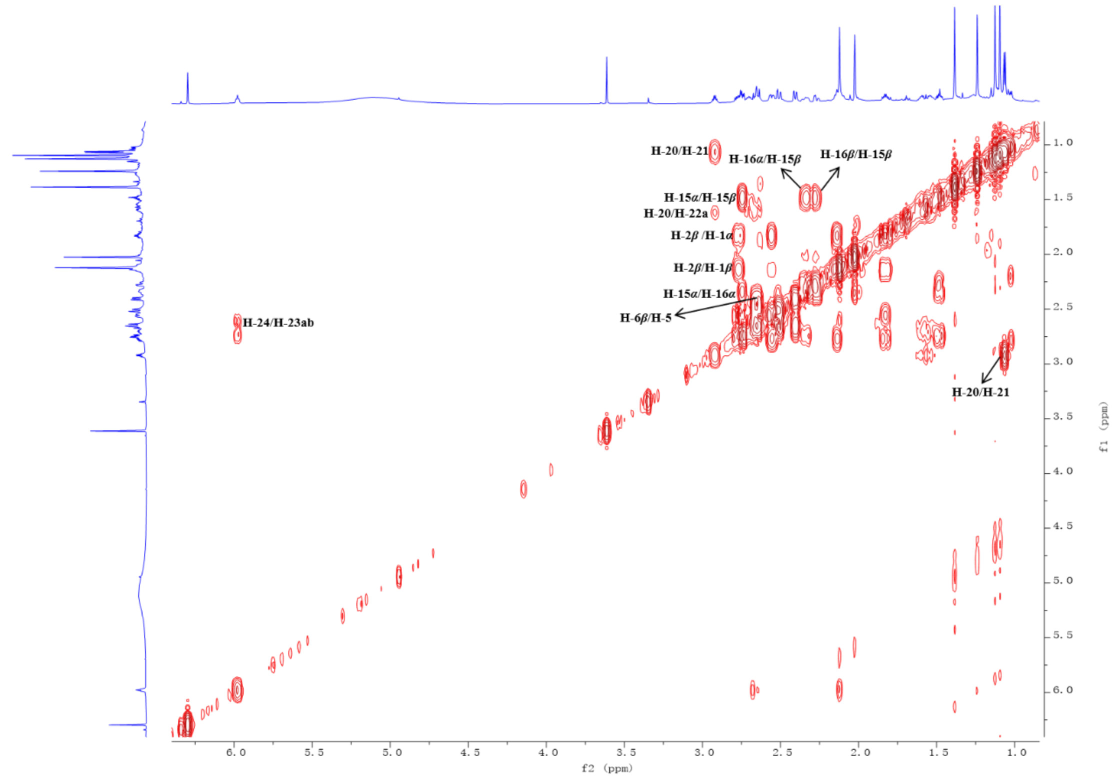

Figure S44 ^{1}H–^{1}H COSY spectrum of kadcoccitane H (**4**) (pyridine-d_5, 800 MHz).

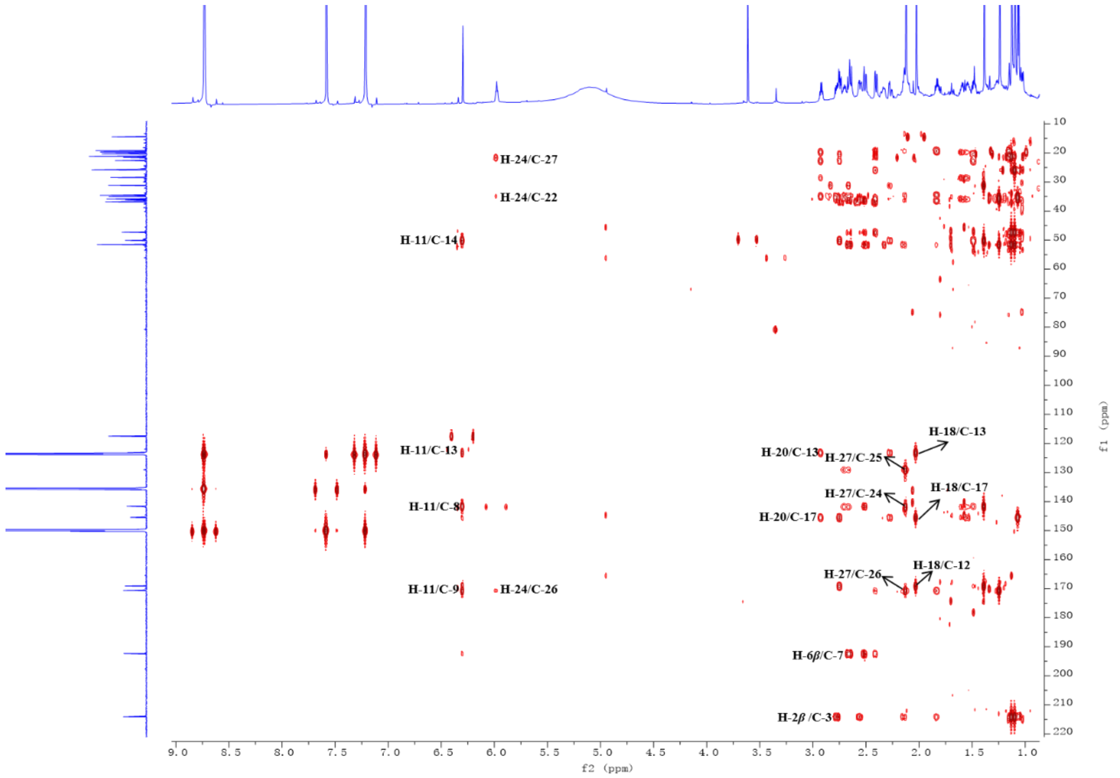

Figure S45 HMBC spectrum of kadcoccitane H (**4**) (pyridine-d_5, 800 MHz).

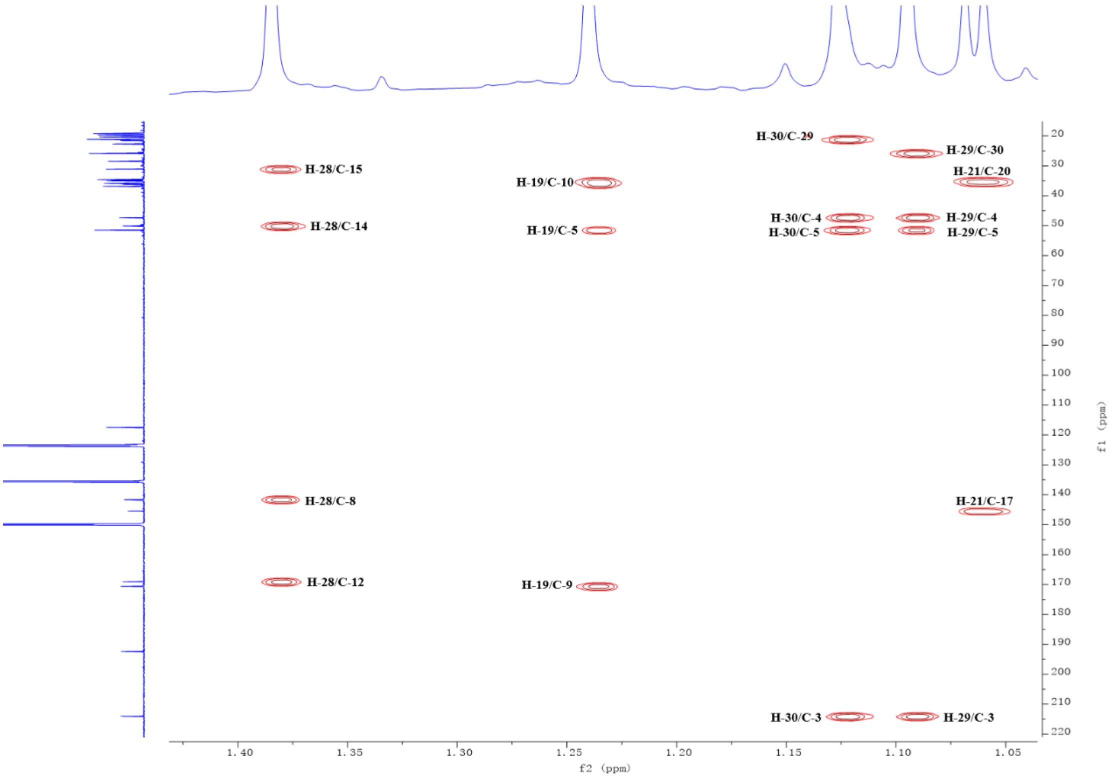

Figure S46 Locally magnified HMBC spectrum of kadcoccitane H (**4**) (pyridine-d_5, 800 MHz).

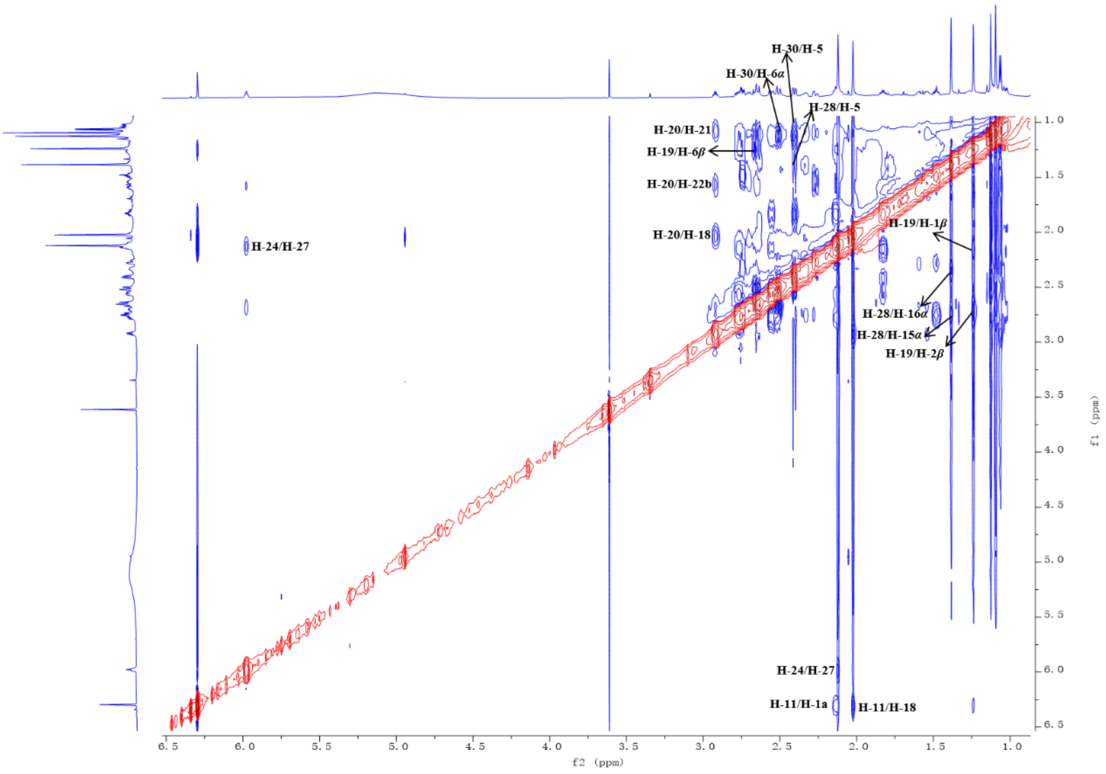

Figure S47 ROESY spectrum of kadcoccitane H (**4**) (pyridine-d_5, 800 MHz).

Qualitative Analysis Report

**Data Filename**	szqq-17.d	**Sample Name**	szqq-17
**Sample Type**	Sample	**Position**	P1-A4
**Instrument Name**	Instrument 1	**User Name**	
**Acq Method**	s.m	**Acquired Time**	2/17/2022 1:20:09 PM
**IRM Calibration Status**	Success	**DA Method**	PCDL.m
**Comment**			

**Sample Group**		**Info.**
**Acquisition SW Version**	6200 series TOF/6500 series Q-TOF B.05.01 (B5125.2)	

User Spectra

Fragmentor Voltage	Collision Energy	Ionization Mode
135	0	ESI

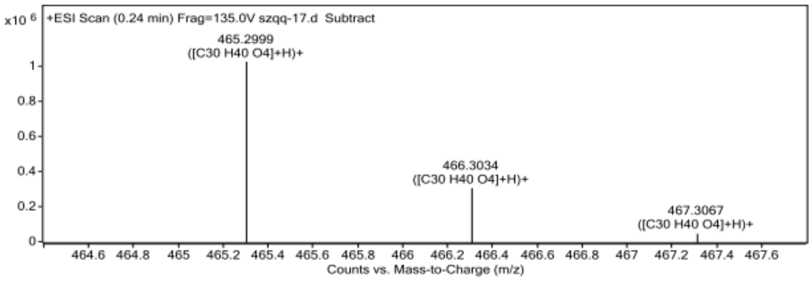

Peak List

m/z	z	Abund	Formula	Ion
293.1554	1	24616.71		
314.6661	1	13328.73		
465.2999	1	1031088.94	C30 H40 O4	(M+H)+
466.3034	1	310423.5	C30 H40 O4	(M+H)+
467.3067	1	53568.06	C30 H40 O4	(M+H)+
544.2728	1	18866.8		
587.2973	1	12159.09		
929.5922	1	11390.21		
967.539	1	14786.14		
968.5423	1	10497.49		

Formula Calculator Element Limits

Element	Min	Max
C	3	60
H	0	120
O	0	30

Formula Calculator Results

Formula	CalculatedMass	CalculatedMz	Mz	Diff. (mDa)	Diff. (ppm)	DBE
C30 H40 O4	464.2927	465.2999	465.2999	0.00	0.00	11.0000

--- End Of Report ---

Figure S48 HRESIMS spectrum of kadcoccitane H (**4**).

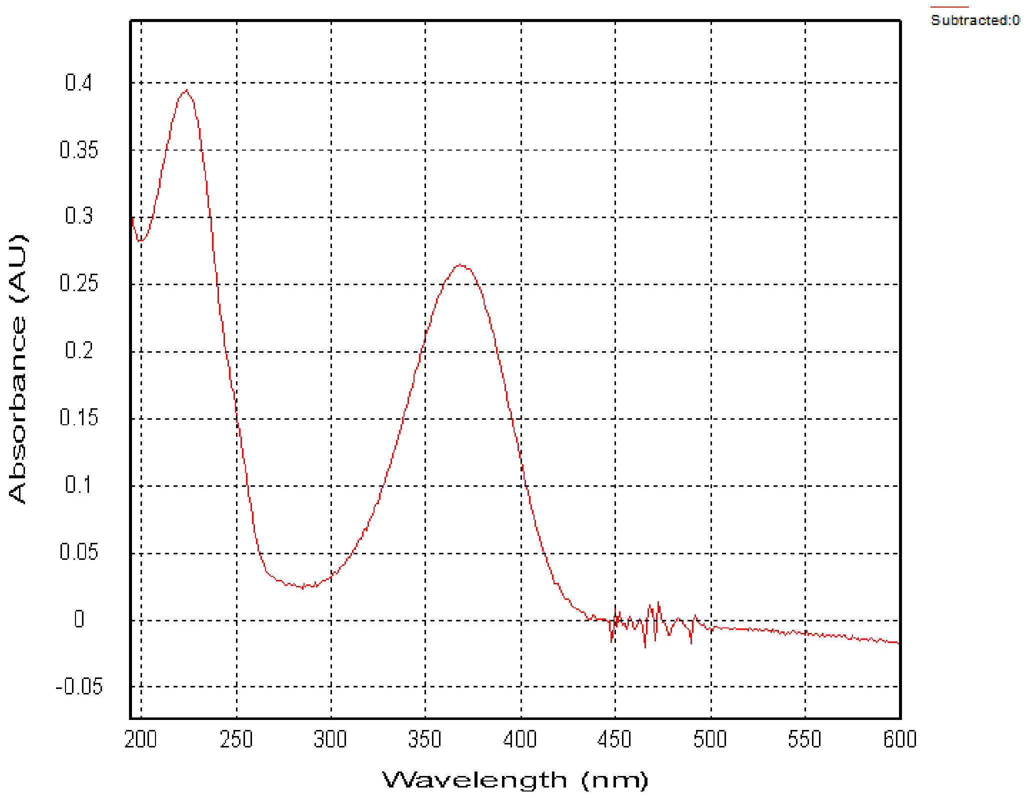

Figure S49 UV spectrum of kadcoccitane H (**4**).

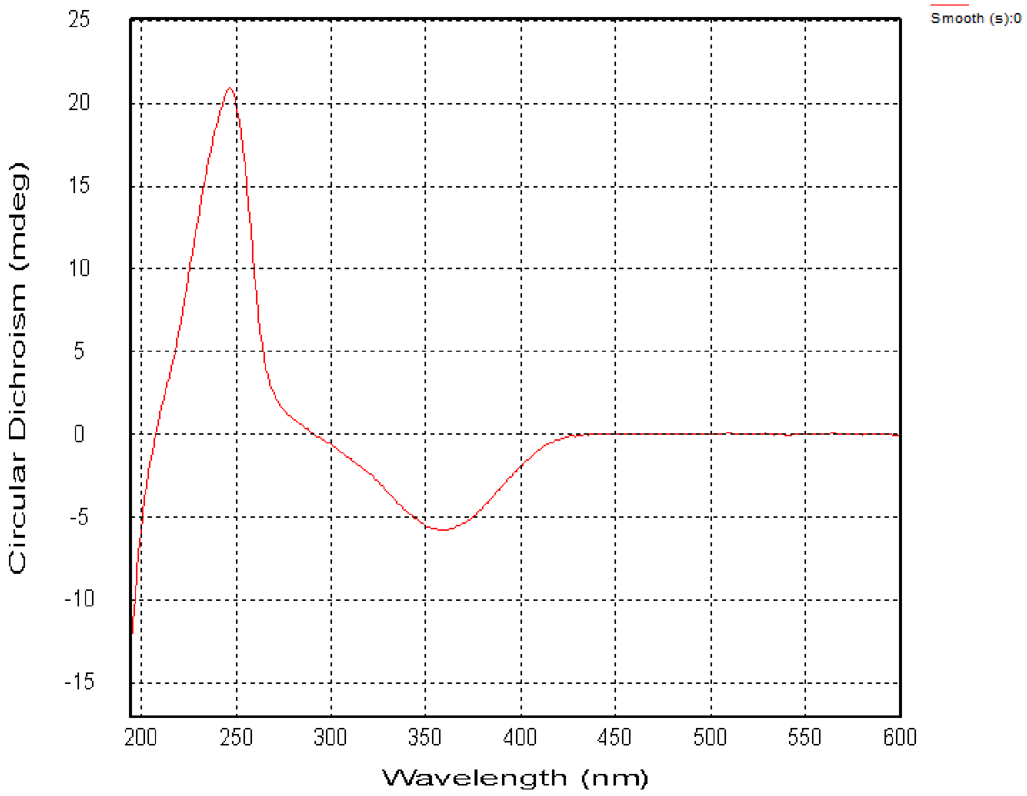

Figure S50 ECD spectrum of kadcoccitane H (**4**).

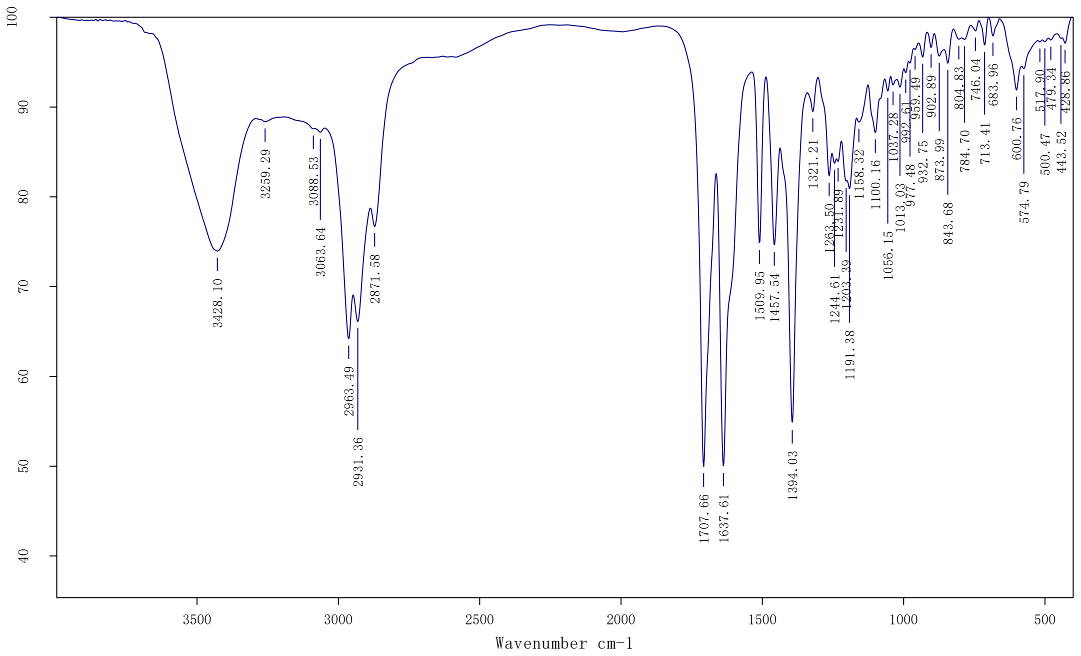

Sample Name: szqq-17
Sample Form: KBr
Path of File: E:\data
Date of Measurement: 2022/2/21

Resolution: 4
Aperture Setting: 6 mm
Number of Background Scans: 16
Number of Sample Scans: 16

Beamsplitter Setting: KBr
Source Setting: MIR
Instrument Type: BRUKER VERTEX 70
Soft Version: OPUS8.1

Figure S51 IR spectrum of kadcoccitane H (**4**).

Rudolph Research Analytical

This sample was measured on an Autopol VI, Serial #91058
Manufactured by Rudolph Research Analytical, Hackettstown, NJ, USA.

Measurement Date : Thursday, 17-FEB-2022

Set Temperature : 20.0

Time Delay : Disabled

Delay between Measurement : Disabled

n	Average	Std.Dev.	% RSD	Maximum	Minimum
5	-275.76	0.00	0.00	-275.76	-275.76

S.No	Sample ID	Time	Result	Scale	OR °Arc	WLG.nm	Lg.mm	Conc.g/100ml	Temp.
1	SZQQ-17	10:48:27 AM	-275.76	SR	-0.546	589	100.00	0.198	19.9
2	SZQQ-17	10:48:33 AM	-275.76	SR	-0.546	589	100.00	0.198	19.9
3	SZQQ-17	10:48:40 AM	-275.76	SR	-0.546	589	100.00	0.198	19.9
4	SZQQ-17	10:48:47 AM	-275.76	SR	-0.546	589	100.00	0.198	19.9
5	SZQQ-17	10:48:54 AM	-275.76	SR	-0.546	589	100.00	0.198	19.9

Figure S52 OR of kadcoccitane H (**4**).

8. Computational data of 4a and 4b.

Methods for quantum Chemical Calculations

Conformational searching of **4a** and **4b** were carried out using the Crest code (version 2.11) using the default iMTD-GC procedure [1]. These conformers were clustered according to their geometry difference represented by maximum difference in distance matrix, and the threshold was set as 0.5 Å. For each isomer, 40 conformers were subjected to DFT geometry optimizations at B3LYP-D3BJ/6-31G(d) level of theory in the gas phase. Frequency analyses of all optimized conformers were undertaken at the same level of theory to ensure that they were true local minima. More accurate energies of optimized conformers were evaluated at M06-2X-D3/6-311+G(2d,p) level of theory in the gas phase, and were then added to thermal correction to Gibbs free energies obtained by frequency analyses to get the Gibbs free energies of each conformer. Those two B3LYP geometries with RMSD below 0.15 Å and difference in Gibbs free energy below 0.15 kcal/mol were regarded as duplicate conformers, and the one with higher energy was removed. Subsequently, Room-temperature (298.15 K) equilibrium populations were calculated according to Boltzmann distribution law:

$$p_i = \frac{n_i}{\sum_j n_j} = \frac{e^{-\Delta G_i/RT}}{\sum_j e^{-\Delta G_j/RT}}$$

Where P_i is the population of the ith conformer; n_i the number of molecules in i^{th} conformer; ΔG is the relative Gibbs free energy (kcal/mol); T is room temperature (298.15 K); R is the ideal gas constant (0.0019858995).

For NMR calculations of each isomer, all dereplicated conformers were subjected to GIAO calculation at mPW1PW91-SCRF/6-31+G(d,p) level of theory (pyridine, IEFPCM solvent model). Then, DP4+ probability analysis based on random conformational amplitudes [2] were undertaken using the calculated NMR shielding tensors and scripts provided by Sarotti, *et al*, and DP4+ probabilities of each structural candidates were obtained.

Those conformers with a population over 2% were subjected to subsequent TDDFT ECD calculations at CAM-B3LYP-SCRF/6-311+G(2d,p) level of theory (MeOH, IEFPCM solvent model), and 36 excited states were calculated for each conformer. The coordinates of calculated ECD spectra and cube files of **4a-1** were generated using the Multiwfn software (version 3.8) [3].

The molecular orbitals were plotted by using the Vcube tcl script (version 2.0) and VMD (version 1.9.3).

The geometry optimization, single-point energy calculations, ECD calculations were all completed in Gaussian 09 program [4].

[1] P. Pracht, F. Bohle, S. Grimme, Automated exploration of the low-energy chemical space with fast quantum chemical methods. *Phys. Chem. Chem. Phys.* 22 (**2020**), 7169–7192.

[2] María M. Zanardi, Maribel O. Marcarino, and Ariel M. Sarotti, Redefining the Impact of Boltzmann Analysis in the Stereochemical Assignment of Polar and Flexible Molecules by NMR Calculations. *Org. Lett.* 22 (**2020**), 52–56.

[3] T. Lu, F. Chen, Multiwfn: A multifunctional wavefunction analyzer. *J. Comput. Chem.* 33 (**2012**), 580–592.

[4] M.J. Frisch, G.W. Trucks, H.B. Schlegel, G.E. Scuseria, M.A. Robb, J.R. Cheeseman, G. Scalmani, V. Barone, B. Mennucci, G.A. Petersson, H. Nakatsuji, M. Caricato, X. Li, H.P. Hratchian, A.F. Izmaylov, J. Bloino, G. Zheng, J.L. Sonnenberg, M. Hada, M. Ehara, K. Toyota, R. Fukuda, J. Hasegawa, M. Ishida, T. Nakajima, Y. Honda, O. Kitao, H. Nakai, T. Vreven, J.A. Montgomery, J.E.P. Jr., F. Ogliaro, M. Bearpark, J.J. Heyd, E. Brothers, K.N. Kudin, V.N. Staroverov, T. Keith, R. Kobayashi, J. Normand, K. Raghavachari, A. Rendell, J.C. Burant, S.S. Iyengar, J. Tomasi, M. Cossi, N. Rega, J.M. Millam, M. Klene, J.E. Knox, J.B. Cross, V. Bakken, C. Adamo, J. Jaramillo, R. Gomperts, R.E. Stratmann, O. Yazyev, A.J. Austin, R. Cammi, C. Pomelli, J.W. Ochterski, R.L. Martin, K. Morokuma, V.G. Zakrzewski, G.A. Voth, P. Salvador, J.J. Dannenberg, S. Dapprich, A.D. Daniels, O. Farkas, J.B. Foresman, J.V. Ortiz, J. Cioslowski, D.J. Fox, Gaussian 09, Revision E.01; Gaussian, Inc., Wallingford CT: **2010**.

Data for NMR calculations

```
*****************************************************************************************
  DP4/DP4+ RANDOM CONFORMATIONAL AMPLITUDE CALCULATOR by Zanardi, Marcarino & Sarotti
                    Org. Lett. 2020, 22, 52-56
*****************************************************************************************

Select DP4 or DP4+ analysis (1 for DP4, 2 for DP4+): 2

- - - - - - - - - - - - - - - - - - - - - - - - - - - - - - - - - - - - - - - -
You have chosen DP4+ analysis (see Grimblat, Zanardi & Sarotti, JOC 2015, 80, 12526)
Warning: the DP4+ parameters were taken at the PCM/mPW1PW91/6-31+G**//B3LYP/6-31G* level
- - - - - - - - - - - - - - - - - - - - - - - - - - - - - - - - - - - - - - - -
Ensemble   Subset of conformers generated by random selection of...   E window (kcal/mol)
  R1          Up to 100 conformations of the full set                  3
  R2          Up to 100 conformations of the full set                  6
  R3          Up to 100 conformations of the full set                  9
  R4            25% of the full set conformations                      3
  R5            25% of the full set conformations                      6
  R6            25% of the full set conformations                      9
  R7            50% of the full set conformations                      3
  R8            50% of the full set conformations                      6
  R9            50% of the full set conformations                      9
  R10           75% of the full set conformations                      3
  R11           75% of the full set conformations                      6
  R12           75% of the full set conformations                      9
  R13              Full set of conformations                           3
  R14              Full set of conformations                           6
  R15              Full set of conformations                           9
- - - - - - - - - - - - - - - - - - - - - - - - - - - - - - - - - - - - - - - -
Choose the strategy to generate the random amplitudes (type 0 for the full exploration): R0
Set the number of iterations (per R strategy): 10000
- - - - - - - - - - - - - - - - - - - - - - - - - - - - - - - - - - - - - - - -
    Averaged Probabilities with the R0 strategy after 10000 iterations
- - - - - - - - - - - - - - - - - - - - - - - - - - - - - - - - - - - - - - - -
Isomer N°     Averaged Probability     N° times ranked #1     N° times ranked #2
---------     --------------------     ------------------     ------------------
1             0.671                    0.672                  0.328
2             0.329                    0.328                  0.672
---------     --------------------     ------------------     ------------------
The most likely isomer is N° 1, with an averaged probability of 67.09%

*****************************************************************************************
              Normal termination. Thanks for using our method
  For further inquiries, please contact Dr. A. Sarotti at sarotti@iquir-conicet.gov.ar
                 Cite this: Org. Lett. 2020, 22, 1, 52-56
*****************************************************************************************
```

Figure S53 DP4+ analysis of **4a** and **4b** based on random conformational amplitudes using integral NMR data.

```
******************************************************************************************
  DP4/DP4+ RANDOM CONFORMATIONAL AMPLITUDE CALCULATOR by Zanardi, Marcarino & Sarotti
                    Org. Lett. 2020, 22, 52-56
******************************************************************************************

Select DP4 or DP4+ analysis (1 for DP4, 2 for DP4+): 2

- - - - - - - - - - - - - - - - - - - - - - - - - - - - - - - - - - - - - - - - - -
You have chosen DP4+ analysis (see Grimblat, Zanardi & Sarotti, JOC 2015, 80, 12526)
Warning: the DP4+ parameters were taken at the PCM/mPW1PW91/6-31+G**//B3LYP/6-31G* level
- - - - - - - - - - - - - - - - - - - - - - - - - - - - - - - - - - - - - - - - - -
Ensemble   Subset of conformers generated by random selection of...   E window (kcal/mol)
  R1         Up to 100 conformations of the full set              3
  R2         Up to 100 conformations of the full set              6
  R3         Up to 100 conformations of the full set              9
  R4           25% of the full set conformations                  3
  R5           25% of the full set conformations                  6
  R6           25% of the full set conformations                  9
  R7           50% of the full set conformations                  3
  R8           50% of the full set conformations                  6
  R9           50% of the full set conformations                  9
  R10          75% of the full set conformations                  3
  R11          75% of the full set conformations                  6
  R12          75% of the full set conformations                  9
  R13             Full set of conformations                       3
  R14             Full set of conformations                       6
  R15             Full set of conformations                       9
- - - - - - - - - - - - - - - - - - - - - - - - - - - - - - - - - - - - - - - - - -
Choose the strategy to generate the random amplitudes (type 0 for the full exploration): R0
Set the number of iterations (per R strategy): 10000
- - - - - - - - - - - - - - - - - - - - - - - - - - - - - - - - - - - - - - - - - -
    Averaged Probabilities with the R0 strategy after 10000 iterations
- - - - - - - - - - - - - - - - - - - - - - - - - - - - - - - - - - - - - - - - - -
Isomer N°     Averaged Probability     N° times ranked #1     N° times ranked #2
---------     --------------------     ------------------     ------------------
1             0.661                    0.662                  0.338
2             0.339                    0.338                  0.662
---------     --------------------     ------------------     ------------------
The most likely isomer is N° 1, with an averaged probability of 66.09%

******************************************************************************************
             Normal termination. Thanks for using our method
  For further inquiries, please contact Dr. A. Sarotti at sarotti@iquir-conicet.gov.ar
                 Cite this: Org. Lett. 2020, 22, 1, 52-56
******************************************************************************************
```

Figure S54 DP4+ analysis of **4a** and **4b** based on random conformational amplitudes using partial NMR data (^{1}H and ^{13}C chemical shifts/shielding tensors in C-24–C-27 moiety were excluded from experimental/calculated data).

Computational data of (5*R**,10*S**,14*R**,20*R**)-**4** (**4a**)

Table S1 Conformational analysis of the B3LYP-D3BJ/6-31G(d) optimized conformers of **4a** in the gas phase (T=298.15 K)

Conformer	E (Hartree)[a]	C (Hartree)[b]	G (kcal/mol)[c]	ΔG (kcal/mol)[d]	Population[e]
**4a-1**	1468.010036	0.571941	920817.404619	0.0	20.79%
**4a-2**	1468.010407	0.572322	920817.398275	0.006344	20.56%
**4a-3**	1468.008004	0.570578	920816.984501	0.420118	10.22%
**4a-4**	1468.009042	0.571691	920816.937978	0.46664	9.45%
**4a-5**	1468.008974	0.571814	920816.818201	0.586418	7.72%
**4a-6**	1468.008866	0.571787	920816.766803	0.637816	7.08%
**4a-7**	1468.009813	0.572793	920816.730169	0.67445	6.65%
**4a-8**	1468.007825	0.571032	-920816.58762	0.816999	5.23%
**4a-9**	1468.009079	0.572364	920816.538669	0.86595	4.82%
**4a-10**	1468.009648	0.573031	920816.477274	0.927345	4.34%
**4a-11**	-1468.00875	0.57244	920816.284475	1.120144	3.13%

[a]Electronic energy obtained at the M06-2X-D3/6-311+G(2d,p) level of theory; [b]Thermal correction to Gibbs free energy obtained at the B3LYP-D3BJ/6-31G(d) level of theory; [c]Gibbs free energy (E + C); [d]The relative Gibbs free energy; [e]The Boltzmann distribution of each conformer.

Table S2 Atomic coordinates (Å) of **4a-1** obtained at the B3LYP-D3BJ/6-31G(d) level of theory in the gas phase.

C	2.867291	1.475565	1.271731	H	2.857710	0.870448	2.187562
C	4.256184	2.105549	1.097520	H	2.121940	2.267048	1.407949
C	5.335815	1.063212	0.860180	H	4.544855	2.689400	1.975514
C	5.068850	-0.001755	-0.224640	H	4.247696	2.793276	0.241537
C	3.592927	-0.504515	-0.110230	H	-0.040396	1.340184	1.476852
C	3.199113	-1.524652	-1.193767	H	-1.116095	-3.077565	-1.356034
C	1.857974	-2.203283	-0.906193	H	-1.380611	-1.349515	-1.603034
C	0.919217	-1.413557	-0.136441	H	-3.556418	-2.337303	-1.182010
C	1.196830	-0.163820	0.352652	H	-2.980979	-3.218853	0.203813
C	2.484082	0.581327	0.075697	H	-4.981938	-0.038215	1.306807
C	0.032523	0.347040	1.054916	H	-5.393731	-3.018157	0.731453
C	-0.978479	-0.568981	0.970129	H	-6.596829	-1.908469	1.403707
C	-2.390400	-0.382655	1.262876	H	-5.134109	-2.299398	2.330342
C	-0.469258	-1.824136	0.292051	H	-5.520693	-1.516301	-1.292177
C	-1.430058	-2.154363	-0.860529	H	-6.448277	-0.236927	-0.537006
C	-2.867404	-2.266668	-0.333613	H	-3.647424	0.060680	-1.779719
C	-3.301028	-1.139230	0.589176	H	-5.169594	0.554241	-2.505299
C	-2.709469	0.734932	2.230303	H	-2.226439	0.538105	3.195087
C	2.188633	1.465511	-1.163901	H	-2.313426	1.689059	1.867039
C	-4.798254	-0.911130	0.676029	H	-3.775754	0.865694	2.411863
C	-5.521362	-2.105682	1.324456	H	-1.353711	-3.227994	1.733534
C	-5.423571	-0.594990	-0.705320	H	0.024047	-3.874805	0.799601
C	-4.642601	0.433486	-1.546541	H	0.296507	-2.722636	2.126220
C	-4.578781	1.779232	-0.889932	H	7.056333	-0.808021	0.100267
C	-3.538938	2.585007	-0.600321	H	5.996679	-1.889046	-0.824124
C	-2.105804	2.289671	-0.862110	H	5.790877	-1.714588	0.929923
C	-3.744636	3.898629	0.116764	H	5.225314	-0.068299	-2.402076
C	-0.375388	-2.991063	1.305061	H	4.861753	1.551676	-1.798516
C	5.420372	0.637834	-1.588862	H	6.486517	0.886183	-1.610141
C	6.033688	-1.178312	0.006284	H	-5.556519	2.156601	-0.581769
O	6.377740	1.089405	1.488697	H	-4.808754	4.089302	0.280317
O	1.608004	-3.331872	-1.330354	H	-3.234245	3.899937	1.085768
O	-1.200315	2.916345	-0.335201	H	-3.323460	4.732545	-0.455690
O	-1.845189	1.286510	-1.733622	H	3.003648	2.151601	-1.401192
H	3.570516	-1.058144	0.840737	H	1.996398	0.854319	-2.051171
H	3.123632	-1.041574	-2.177250	H	1.298336	2.073891	-0.967477
H	3.942917	-2.317971	-1.295948	H	-0.876618	1.167266	-1.711953

Table S3 Atomic coordinates (Å) of **4a-2** obtained at the B3LYP-D3BJ/6-31G(d) level of theory in the gas phase.

C	2.908876	1.126513	1.656098	H	2.510125	0.539628	2.490040
C	4.438531	1.286619	1.842991	H	2.428377	2.108814	1.707231
C	5.188346	1.285305	0.524154	H	4.816534	0.438992	2.429184
C	5.016142	0.014969	-0.326911	H	4.688301	2.200355	2.386572
C	3.611278	-0.612976	-0.044472	H	-0.034251	1.253369	1.580259
C	3.158291	-1.583938	-1.146850	H	-1.128155	-3.072061	-1.390540
C	1.841628	-2.286346	-0.824679	H	-1.335195	-1.332189	-1.612310
C	0.902485	-1.489815	-0.063148	H	-3.551065	-2.268754	-1.294204
C	1.198856	-0.261952	0.466006	H	-3.054255	-3.189808	0.095488
C	2.521952	0.445907	0.312461	H	-5.008375	0.035711	1.166442
C	0.022661	0.267664	1.137681	H	-5.484262	-2.922659	0.529642
C	-1.011228	-0.614108	0.992215	H	-6.682926	-1.786014	1.163876
C	-2.428356	-0.393842	1.233825	H	-5.275730	-2.233776	2.148998
C	-0.511672	-1.868908	0.306042	H	-5.468163	-1.390028	-1.477314
C	-1.435224	-2.149790	-0.888561	H	-6.395177	-0.096073	-0.747078
C	-2.893867	-2.231880	-0.419173	H	-3.521769	0.148803	-1.827164
C	-3.331732	-1.108909	0.507126	H	-4.986406	0.673090	-2.645783
C	-2.755795	0.710685	2.213470	H	-3.824769	0.858088	2.364527
C	2.339299	1.533960	-0.774588	H	-2.308029	0.483227	3.188514
C	-4.823716	-0.834645	0.532238	H	-2.329313	1.664060	1.884485
C	-5.611297	-2.014741	1.129789	H	-0.089442	-3.941201	0.789984
C	-5.373863	-0.480696	-0.871687	H	0.160578	-2.822439	2.149031
C	-4.523590	0.537857	-1.656434	H	-1.485713	-3.277268	1.684482
C	-4.478521	1.877881	-0.986908	H	7.108277	-0.559328	-0.047092
C	-3.445275	2.658906	-0.617942	H	6.021827	-1.921761	-0.379363
C	-2.003505	2.335349	-0.782347	H	6.035445	-1.176486	1.225608
C	-3.673092	3.967574	0.101443	H	4.501910	0.960327	-2.245769
C	-0.486058	-3.057727	1.297846	H	6.224499	0.897570	-1.881597
C	5.287176	0.342349	-1.804504	H	5.374585	-0.573536	-2.396040
C	6.113066	-0.968670	0.152692	H	-5.467512	2.272020	-0.742082
O	5.905792	2.207065	0.182222	H	-4.741783	4.181065	0.189858
O	1.597540	-3.421314	-1.234688	H	-3.234902	3.943408	1.104883
O	-1.126751	2.923700	-0.170012	H	-3.192191	4.798784	-0.426112
O	-1.701382	1.352291	-1.663418	H	1.477890	2.164107	-0.527629
H	3.714446	-1.220062	0.867051	H	3.215334	2.184953	-0.848966
H	3.016338	-1.049179	-2.095208	H	2.162133	1.087230	-1.758856
H	3.911338	-2.354972	-1.334521	H	-0.740915	1.204512	-1.568598

Table S4 Atomic coordinates (Å) of **4a-3** obtained at the B3LYP-D3BJ/6-31G(d) level of theory in the gas phase.

C	3.021190	1.506305	1.303171	H	2.988798	0.868897	2.196115
C	4.424898	2.110930	1.164199	H	2.292756	2.309561	1.464445
C	5.483594	1.052271	0.903528	H	4.717279	2.658162	2.064291
C	5.203168	0.026302	-0.214601	H	4.441907	2.827073	0.331792
C	3.712989	-0.441816	-0.137682	H	0.103176	1.420556	1.502080
C	3.307206	-1.409830	-1.263001	H	-1.032249	-2.853980	-1.515496
C	1.950408	-2.069457	-1.007383	H	-1.301931	-1.110384	-1.668187
C	1.020754	-1.285310	-0.219969	H	-3.474267	-2.075027	-1.290079
C	1.318171	-0.057696	0.309778	H	-2.908429	-3.099554	-0.001181
C	2.628217	0.664457	0.073143	H	-4.821342	-0.001618	1.434705
C	0.156332	0.451734	1.019047	H	-6.191157	-1.980329	2.056724
C	-0.868268	-0.450944	0.911875	H	-4.551541	-2.108780	2.727579
C	-2.270022	-0.314252	1.257499	H	-5.005335	-3.058515	1.303294
C	-0.374962	-1.686454	0.188622	H	-5.621351	-1.831560	-0.861576
C	-1.343885	-1.956029	-0.974097	H	-6.594746	-0.642510	-0.018547
C	-2.779164	-2.101202	-0.444973	H	-4.200160	-0.068435	-1.866580
C	-3.187189	-1.065654	0.585662	H	-5.902297	0.211216	-2.206133
C	-2.648972	0.723365	2.296578	H	-3.311254	0.304795	3.061533
C	2.379627	1.591769	-1.143153	H	-1.759528	1.096301	2.809575
C	-4.670826	-0.935278	0.884774	H	-3.159950	1.582431	1.850120
C	-5.131239	-2.089097	1.798601	H	0.376017	-2.660569	1.993020
C	-5.573907	-0.854793	-0.364480	H	-1.283238	-3.121656	1.581350
C	-5.148566	0.201356	-1.405608	H	0.084659	-3.759975	0.626545
C	-5.063167	1.581352	-0.826370	H	5.859522	-1.739203	0.898972
C	-3.984652	2.374370	-0.688111	H	7.162870	-0.843242	0.117461
C	-2.635823	1.927801	-1.135575	H	6.090438	-1.867106	-0.855713
C	-4.071147	3.729121	-0.025833	H	5.071637	1.634655	-1.742442
C	-0.299985	-2.887916	1.162532	H	6.668709	0.906916	-1.554225
C	5.594553	0.695128	-1.554059	H	5.385767	0.021919	-2.391249
C	6.132411	-1.182482	-0.003928	H	-6.001554	1.971367	-0.426372
O	6.520591	1.038781	1.540220	H	-3.466661	3.768314	0.886694
O	1.684172	-3.180889	-1.464039	H	-3.694531	4.520143	-0.684983
O	-2.373508	1.107305	-1.995025	H	-5.107799	3.963815	0.232822
O	-1.650984	2.557825	-0.442167	H	1.519560	2.239456	-0.933954
H	3.662789	-1.028457	0.792017	H	3.224622	2.246243	-1.362581
H	3.248721	-0.887226	-2.227042	H	2.146795	1.019241	-2.044435
H	4.034648	-2.214564	-1.389998	H	-0.823978	2.109141	-0.703427

Computational data of (5*R**,10*S**,14*R**,20*S**)-**4** (**4b**)

Table S5 Conformational analysis of the B3LYP-D3BJ/6-31G(d) optimized conformers of **4b** in the gas phase (T=298.15 K)

Conformer	E (Hartree)[a]	C (Hartree)[b]	G (kcal/mol)[c]	ΔG (kcal/mol)[d]	Population[e]
**4b-1**	1468.002816	0.565912	920816.656965	0.0	24.74%
**4b-2**	1468.006168	0.569671	-920816.40215	0.254815	16.09%
**4b-3**	1468.006784	0.570398	920816.332485	0.32448	14.30%
**4b-4**	1468.007016	0.570664	920816.311137	0.345828	13.80%
**4b-5**	-1468.00606	0.569756	920816.281017	0.375948	13.11%
**4b-6**	1468.004687	0.568767	920816.040076	0.616889	8.73%
**4b-7**	1468.003935	0.568442	920815.772159	0.884806	5.55%
**4b-8**	1468.003023	0.567921	920815.526323	1.130642	3.67%

[a]Electronic energy obtained at the M06-2X-D3/6-311+G(2d,p) level of theory; [b]Thermal correction to Gibbs free energy obtained at the B3LYP-D3BJ/6-31G(d) level of theory; [c]Gibbs free energy (E + C); [d]The relative Gibbs free energy; [e]The Boltzmann distribution of each conformer.

Table S6 Atomic coordinates (Å) of **4b-1** obtained at the B3LYP-D3BJ/6-31G(d) level of theory in the gas phase.

C	-3.922444	1.767471	-1.172474	H	-3.121499	1.625833	-1.905428
C	-5.287532	1.679412	-1.900795	H	-3.793165	2.773411	-0.759509
C	-6.361661	1.041980	-1.039973	H	-5.173693	1.052451	-2.794459
C	-6.061267	-0.393103	-0.576023	H	-5.637727	2.660642	-2.228452
C	-4.514198	-0.580460	-0.435789	H	-1.328363	2.427236	0.061293
C	-4.132927	-1.780082	0.446183	H	0.170999	-2.401639	1.806267
C	-2.629618	-2.044732	0.463046	H	-0.144795	-0.838573	2.567064
C	-1.806132	-0.857469	0.369128	H	2.250622	-1.169097	2.726645
C	-2.303343	0.400191	0.156465	H	2.371630	-1.720600	1.080454
C	-3.766218	0.726320	-0.026768	H	3.769337	1.844251	1.125239
C	-1.205030	1.353852	0.151223	H	3.638823	0.200385	3.707448
C	-0.028479	0.689374	0.356042	H	2.981155	1.831153	3.493696
C	1.305535	1.198870	0.613288	H	4.728246	1.548035	3.354444
C	-0.298996	-0.800250	0.419894	H	5.663854	0.338282	1.384006
C	0.342864	-1.325172	1.713191	H	4.600783	-1.049706	1.607520
C	1.844199	-1.000227	1.721982	H	3.682813	-0.678230	-0.712564
C	2.197107	0.405039	1.268643	H	4.732812	0.736481	-0.878818
C	1.610017	2.627608	0.203729	H	2.544913	2.696622	-0.361247
C	-4.286847	1.339259	1.294852	H	0.817938	3.022582	-0.437116
C	3.592466	0.884348	1.619591	H	1.702758	3.295117	1.069068
C	3.740436	1.128410	3.134077	H	0.039888	-2.578828	-0.777488
C	4.687596	-0.074559	1.111477	H	-0.184025	-1.099460	-1.737306
C	4.649053	-0.250928	-0.419466	H	1.354555	-1.376408	-0.908371
C	5.745785	-1.151038	-0.906197	H	-7.663846	-1.225582	-1.810962
C	7.012441	-0.829579	-1.235484	H	-6.349305	-2.365479	-1.464264
C	7.483943	0.576006	-1.151144	H	-6.115519	-1.091081	-2.669081
C	7.999079	-1.866773	-1.714409	H	-6.826622	-1.773303	0.928001
C	0.271685	-1.511628	-0.831544	H	-6.495939	-0.155867	1.567495
C	-6.863615	-0.704712	0.697762	H	-7.905969	-0.417215	0.544016
C	-6.578898	-1.321521	-1.703477	H	5.486836	-2.207753	-0.981036
O	-7.401217	1.613254	-0.767105	H	7.543046	-2.861032	-1.709426
O	-2.174483	-3.181665	0.590836	H	8.343714	-1.648511	-2.731250
O	6.899818	1.521691	-0.650548	H	8.892538	-1.892738	-1.080288
O	8.715354	0.731490	-1.709403	H	-5.304767	1.726402	1.190820
H	-4.132507	-0.813548	-1.440626	H	-4.274871	0.608795	2.109111
H	-4.449123	-1.611050	1.483935	H	-3.644725	2.175355	1.591656
H	-4.629675	-2.696174	0.113034	H	8.933089	1.675221	-1.595942

Table S7 Atomic coordinates (Å) of **4b-2** obtained at the B3LYP-D3BJ/6-31G(d) level of theory in the gas phase.

C	-3.171204	1.573859	1.279982	H	-2.728803	2.192694	0.488898
C	-4.647174	1.957552	1.456105	H	-2.623477	1.803603	2.201228
C	-5.479096	1.626311	0.228061	H	-4.762488	3.025811	1.657471
C	-5.340722	0.206844	-0.360802	H	-5.072154	1.419223	2.313758
C	-3.831660	-0.202436	-0.382071	H	-0.380303	1.083458	1.984972
C	-3.584663	-1.632465	-0.894507	H	0.746825	-3.237959	-0.973650
C	-2.105471	-1.907245	-1.170412	H	0.625540	-2.912349	0.758356
C	-1.174622	-1.144719	-0.365646	H	2.983530	-3.245296	0.304067
C	-1.554514	-0.211766	0.562363	H	2.944523	-2.234930	-1.111511
C	-2.997736	0.087069	0.909619	H	4.680095	-0.225523	1.646637
C	-0.368575	0.355396	1.182675	H	3.961974	-2.080662	3.136079
C	0.744076	-0.225091	0.641954	H	5.665601	-2.230718	2.659792
C	2.128417	-0.139210	1.075599	H	4.443944	-3.267309	1.912976
C	0.330887	-1.167834	-0.468409	H	6.441077	-1.393378	0.458543
C	1.007632	-2.518642	-0.191801	H	5.285753	-2.385139	-0.415850
C	2.530407	-2.330938	-0.097890	H	6.105480	-0.512054	-1.812163
C	2.985206	-1.142162	0.733808	H	4.368864	-0.400518	-1.582405
C	2.455362	1.036301	1.968379	H	3.516014	1.124065	2.202248
C	-3.333542	-0.803732	2.131760	H	2.139371	1.966363	1.485687
C	4.430254	-1.189322	1.197502	H	1.911878	0.954513	2.918689
C	4.634105	-2.256334	2.289743	H	0.354037	-1.263335	-2.635117
C	5.426639	-1.396301	0.036969	H	0.304707	0.394389	-1.984876
C	5.323207	-0.321787	-1.063181	H	1.822107	-0.524634	-1.942769
C	5.513906	1.056494	-0.499930	H	-5.246292	0.808074	-2.462220
C	4.706892	2.130454	-0.550227	H	-6.880812	0.684488	-1.810290
C	3.400743	2.161440	-1.263601	H	-5.974536	-0.789357	-2.200590
C	5.009951	3.400910	0.205254	H	-6.167003	-1.756946	0.118600
C	0.736856	-0.603591	-1.851651	H	-5.994833	-0.728171	1.544329
C	-6.242484	-0.727100	0.481537	H	-7.286639	-0.412894	0.381761
C	-5.891349	0.223579	-1.797505	H	6.421317	1.171034	0.097288
O	-6.240616	2.447143	-0.248899	H	4.216639	3.614356	0.928998
O	-1.754635	-2.733042	-2.014184	H	5.962232	3.320640	0.736766
O	2.439281	2.808231	-0.892614	H	5.060698	4.264329	-0.468972
O	3.368107	1.440725	-2.413707	H	-2.573538	-0.656356	2.906715
H	-3.382162	0.464736	-1.133034	H	-4.302699	-0.565183	2.573652
H	-3.936088	-2.376361	-0.167184	H	-3.330615	-1.864833	1.869421
H	-4.122434	-1.831247	-1.824301	H	2.455206	1.525702	-2.748829

Table S8 Atomic coordinates (Å) of **4b-3** obtained at the B3LYP-D3BJ/6-31G(d) level of theory in the gas phase.

C	-3.081666	1.920605	0.622445	H	-2.349847	2.309774	-0.093118
C	-4.489289	2.441999	0.235593	H	-2.800607	2.318454	1.603226
C	-5.581247	1.417536	0.483505	H	-4.501797	2.670204	-0.838129
C	-5.413595	0.084584	-0.265173	H	-4.745546	3.359770	0.769314
C	-3.890715	-0.206138	-0.475644	H	-0.383979	1.359897	1.698408
C	-3.597383	-1.684600	-0.774440	H	0.713702	-3.257254	-0.825048
C	-2.130827	-1.937894	-1.116458	H	0.577469	-2.793680	0.874991
C	-1.190599	-1.095253	-0.406158	H	2.938931	-3.201131	0.440126
C	-1.563105	-0.053741	0.399803	H	2.918322	-2.242155	-1.014087
C	-2.989171	0.368991	0.659518	H	4.606863	-0.180131	1.775039
C	-0.372180	0.554450	0.972908	H	5.384765	-2.152106	3.011336
C	0.735092	-0.111098	0.525641	H	4.164649	-3.173280	2.240491
C	2.101898	-0.033695	1.013830	H	3.660686	-1.868437	3.326426
C	0.316390	-1.149814	-0.494892	H	6.392431	-1.524218	0.842756
C	0.972576	-2.480404	-0.099669	H	5.265072	-2.523835	-0.059392
C	2.495696	-2.300763	-0.001473	H	6.323625	-0.802100	-1.500436
C	2.942061	-1.083039	0.789693	H	4.579336	-0.608874	-1.469627
C	2.423565	1.177139	1.861745	H	2.079421	2.086919	1.360482
C	-3.381770	-0.132689	2.069422	H	1.907551	1.119315	2.829157
C	4.341218	-1.161134	1.374171	H	3.488537	1.294405	2.060509
C	4.387310	-2.148317	2.556647	H	0.368842	-1.468831	-2.637657
C	5.421415	-1.510115	0.328826	H	0.260751	0.240819	-2.173175
C	5.475733	-0.526596	-0.856883	H	1.805572	-0.597287	-2.029730
C	5.661760	0.886629	-0.390758	H	-5.643259	1.127969	-2.196140
C	4.871980	1.956720	-0.594165	H	-7.154379	0.472968	-1.532882
C	3.656625	1.854366	-1.444738	H	-5.954561	-0.612740	-2.260633
C	5.146689	3.292423	0.052918	H	-7.192465	-0.676143	0.692059
C	0.726961	-0.719343	-1.926443	H	-6.245711	-1.926825	-0.139557
C	-6.180409	-1.023934	0.474261	H	-5.712190	-1.291012	1.424231
C	-6.082104	0.286585	-1.648507	H	6.525839	1.042709	0.258931
O	-6.541960	1.651611	1.193270	H	6.090348	3.263876	0.605519
O	-1.795051	-2.823295	-1.902749	H	5.207262	4.095544	-0.690729
O	3.469655	1.080235	-2.364124	H	4.345286	3.565648	0.747782
O	2.713016	2.772445	-1.097464	H	-4.356699	0.251580	2.383484
H	-3.585810	0.347210	-1.376166	H	-3.408426	-1.225299	2.113849
H	-3.846058	-2.310673	0.092507	H	-2.641562	0.209538	2.800434
H	-4.202229	-2.051431	-1.609021	H	1.957129	2.602117	-1.690151

Data for TDDFT calculation of (5*R*,10*S*,14*R*,20*R*)-**4** (**4aA**)

Table S9 Key transitions, oscillator strengths, and rotatory strengths in the ECD spectrum of conformer **4a-1** at the CAM-B3LYP-SCRF/6-311+G(2d,p) (methanol, IEFPCM solvent model) level of theory.

Num[a]	*Transition*[b]	*CI-coeff*[c]	*ΔE (eV)*[d]	*λ (nm)*[e]	*f*[f]	R_{vel}[g]	R_{len}[h]
1	126->127	0.6966	3.5138	352.85	0.3907	-86.3314	-86.1017
2	123->127	-0.34572	3.9494	313.93	0.0025	-9.5246	-10.111
	125->127	0.53524					
3	123->129	0.47579	4.3484	285.12	0.0003	-9.1433	-9.415
	125->129	0.40829					
4	126->128	0.69066	4.6389	267.27	0.0076	22.8521	23.0719
5	122->127	-0.4109	5.1411	241.16	0.0935	103.0844	103.3192
	124->127	0.49735					
6	119->128	0.28731	5.2721	235.17	0.0136	43.6378	43.7517
	120->128	-0.38936					
	121->128	0.42822					
7	126->134	0.50496	5.4890	225.88	0.0420	165.6532	166.7511
8	121->127	-0.22824	5.6316	220.16	0.2135	-19.2123	-19.9025
	122->128	0.363					
	124->128	0.37866					
9	126->130	0.48733	5.6595	219.07	0.0040	-16.2979	-16.0236
	126->135	0.24366					
10	120->127	0.31805	5.8114	213.35	0.4078	-148.0526	-148.4582
	121->127	0.35184					
11	126->129	0.45703	5.9058	209.93	0.0015	2.5512	2.6533
	126->131	-0.33091					
12	118->127	-0.23054	5.9237	209.30	0.0260	-9.9135	-9.9498
	123->127	0.47341					
	125->127	0.26478					
13	118->127	0.51449	5.9459	208.52	0.0064	9.3253	9.3588
14	126->129	0.41936	5.9807	207.31	0.0374	-11.1015	-11.1463
	126->131	0.3867					
15	122->127	0.50287	6.0344	205.46	0.0159	5.1864	5.1631
	124->127	0.39299					
16	119->127	-0.33827	6.1513	201.56	0.0206	19.6765	19.6784
	121->127	0.24939					
	126->132	0.35518					
17	119->127	0.23959	6.1946	200.15	0.0775	-36.0726	-36.3129
	126->132	0.43178					
18	126->131	0.2779	6.2559	198.19	0.0026	-5.3008	-5.2943
	126->133	0.48471					
19	122->128	0.48128	6.3688	194.67	0.0020	-3.8926	-3.9866

Num[a]	*Transition*[b]	*CI-coeff*[c]	*ΔE (eV)*[d]	*λ (nm)*[e]	*f*[f]	R_{vel}[g]	R_{len}[h]
	124->128	-0.41582					
20	117->127	0.43878	6.4620	191.87	0.0128	32.1019	32.0316
21	126->135	0.37807	6.4851	191.18	0.0251	-19.959	-19.7901
	126->136	0.3017					
22	126->137	0.23496	6.5613	188.96	0.0173	24.813	24.995
	126->138	0.40303					
23	126->137	0.48304	6.5984	187.90	0.0058	-2.5076	-2.5326
	126->138	-0.25671					
24	126->136	0.36726	6.6295	187.02	0.0647	-21.8005	-22.1194
25	125->130	0.39569	6.6957	185.17	0.0202	13.8246	14.0971
26	114->127	0.28199	6.7151	184.63	0.0059	9.5018	9.5001
	125->134	-0.22511					
27	126->136	-0.27969	6.7259	184.34	0.0161	-46.335	-45.9656
	126->138	0.30477					
28	116->127	0.29011	6.7942	182.48	0.0231	-15.7718	-15.7583
	117->127	0.23997					
	119->127	0.2567					
29	123->130	0.28706	6.8071	182.14	0.0357	14.16	13.4405
	125->136	0.24172					
30	117->127	0.30557	6.8579	180.79	0.0025	-0.152	-0.443
	119->127	-0.3336					
	120->127	0.33968					
	121->127	-0.2277					
31	126->139	0.33379	6.8969	179.77	0.0094	1.8459	1.9061
	126->140	0.23549					
32	125->128	0.42965	6.9003	179.68	0.0026	11.2743	11.3507
33	126->140	0.36054	6.9321	178.86	0.0046	-3.4143	-3.6549
	126->142	0.2609					
34	113->128	0.29461	6.9888	177.40	0.0015	-17.6581	-17.9056
	125->128	0.33817					
35	115->127	0.36615	7.0193	176.63	0.0200	48.3529	48.2369
	116->127	0.30241					
36	123->129	-0.33906	7.0533	175.78	0.0152	-24.9525	-24.9644
	125->129	0.35783					

[a]Number of the excited states; [b]Only transitions with contribution over 10.0% were listed; [c]Configuration-interaction coefficient; [d]Excitation energy; [e]Wavelength; [f]Oscillator strength; [g]Rotatory strength in velocity form (10^{-40} cgs); [h]Rotatory strength in length form (10^{-40} cgs).

Table S10 Key transitions, oscillator strengths, and rotatory strengths in the ECD spectrum of conformer **4a-2** at the CAM-B3LYP-SCRF/6-311+G(2d,p) (methanol, IEFPCM solvent model) level of theory.

Num [a]	***Transition*** [b]	***CI-coeff*** [c]	***ΔE (eV)*** [d]	***λ (nm)*** [e]	***f*** [f]	R_{vel} [g]	R_{len} [h]
1	126->127	0.69672	3.5560	348.66	0.3941	-89.8359	-89.7014
2	123->127	-0.32533	3.9678	312.48	0.0022	-11.1448	-11.7397
	125->127	0.52441					
3	123->129	0.3789	4.3809	283.01	0.0005	14.2445	14.4443
	124->129	0.23874					
	125->129	0.40109					
4	126->128	0.69087	4.6633	265.87	0.0073	21.8241	22.0481
5	122->127	0.43043	5.1768	239.50	0.0934	109.2169	109.48
	124->127	0.47234					
6	119->128	0.30133	5.2745	235.06	0.0174	48.4786	48.5622
	120->128	-0.38703					
	121->128	0.41971					
7	126->129	0.23427	5.4812	226.20	0.0391	141.9417	142.7167
	126->134	0.48669					
8	126->129	0.4128	5.6140	220.85	0.0710	48.1828	47.827
	126->130	0.31707					
9	121->127	0.2822	5.6557	219.22	0.1607	-85.9555	-85.8742
	122->128	-0.28132					
	123->128	-0.23464					
	124->128	0.30877					
10	120->127	0.30622	5.8204	213.02	0.3423	-113.1504	-113.385
	121->127	0.35005					
11	123->127	0.44001	5.9077	209.87	0.0695	-16.213	-16.0827
	125->127	0.31321					
12	126->131	0.52145	5.9380	208.80	0.0388	8.1778	8.1631
	126->133	-0.27139					
13	118->127	0.54526	5.9801	207.33	0.0062	9.3748	9.3387
	119->127	-0.23547					
	120->127	-0.24648					
14	126->129	-0.39652	6.0214	205.91	0.0309	-16.735	-16.9081
	126->130	0.44506					
	126->132	-0.22958					
15	122->127	0.46084	6.0632	204.49	0.0144	5.1936	5.1536
	123->127	0.28637					
	124->127	-0.36108					
16	119->127	0.36444	6.1832	200.52	0.0475	25.3769	25.4821
	121->127	-0.29515					
17	126->132	0.352	6.2371	198.78	0.0371	-30.2698	-30.5379

Num[a]	*Transition*[b]	*CI-coeff*[c]	*ΔE (eV)*[d]	*λ (nm)*[e]	*f*[f]	R_{vel}[g]	R_{len}[h]
	126->133	-0.34846					
18	126->131	0.26587	6.2963	196.92	0.0032	-0.0959	-0.0719
	126->132	0.3296					
	126->133	0.32322					
	126->137	-0.24275					
19	122->128	0.48047	6.3898	194.04	0.0015	-3.5098	-3.6058
	124->128	0.42146					
20	126->135	0.36295	6.5084	190.50	0.0187	4.2659	4.451
	126->136	-0.27946					
21	117->127	0.34325	6.5169	190.25	0.0089	32.5337	32.5361
22	126->137	0.23581	6.5527	189.21	0.0147	16.4012	16.5241
	126->138	0.36706					
23	126->136	0.41675	6.6113	187.53	0.0213	4.3493	4.2165
24	126->137	0.35913	6.6784	185.65	0.0666	2.2236	2.0247
25	125->130	0.31533	6.6906	185.31	0.0129	8.977	8.9541
26	126->138	0.25462	6.7276	184.29	0.0088	8.752	8.6057
27	114->127	0.28962	6.7393	183.97	0.0148	-51.379	-51.1592
28	116->127	-0.24213	6.8231	181.71	0.0249	-22.0023	-22.0572
	117->127	0.35171					
29	125->132	0.24885	6.8312	181.50	0.0365	-17.5536	-18.3382
30	119->127	0.41146	6.8807	180.19	0.0051	-0.1173	-0.4237
	120->127	-0.36263					
	121->127	0.25296					
31	126->139	0.39948	6.9154	179.29	0.0072	-12.8761	-12.7316
32	125->128	0.44489	6.9168	179.25	0.0077	30.4567	30.2443
33	126->140	0.35543	6.9494	178.41	0.0019	-5.6117	-5.8519
34	113->128	0.35474	7.0006	177.10	0.0020	-2.8923	-3.0649
	125->128	0.36097					
35	115->127	0.31005	7.0567	175.70	0.0088	3.4291	3.2559
	116->127	-0.24486					
36	116->127	0.2973	7.0756	175.23	0.0018	-3.1316	-3.1428

[a]Number of the excited states; [b]Only transitions with contribution over 10.0% were listed; [c]Configuration-interaction coefficient; [d]Excitation energy; [e]Wavelength; [f]Oscillator strength; [g]Rotatory strength in velocity form (10^{-40} cgs); [h]Rotatory strength in length form (10^{-40} cgs).

Table S11 Key transitions, oscillator strengths, and rotatory strengths in the ECD spectrum of conformer **4a-3** at the CAM-B3LYP-SCRF/6-311+G(2d,p) (methanol, IEFPCM solvent model) level of theory.

Num[a]	*Transition*[b]	*CI-coeff*[c]	*ΔE (eV)*[d]	*λ (nm)*[e]	*f*[f]	R_{vel}[g]	R_{len}[h]
1	126->127	0.69761	3.4737	356.92	0.4091	-62.4245	-62.6581
2	123->127	0.30759	3.9431	314.43	0.0020	-7.6041	-8.1388
	125->127	0.53952					
3	123->129	0.48068	4.3477	285.17	0.0003	-9.1645	-9.4383
	125->129	-0.39704					
4	126->128	0.68483	4.7518	260.92	0.0074	16.4644	16.9064
5	121->128	-0.29358	5.1363	241.39	0.0755	143.7435	143.6696
	122->127	0.51455					
6	121->128	0.51034	5.1654	240.03	0.0694	-6.9033	-7.1321
	122->127	0.33121					
7	126->132	0.60906	5.3941	229.85	0.0698	89.5678	89.9816
8	124->128	0.58863	5.6483	219.51	0.1713	24.9275	23.6054
9	126->129	0.31812	5.6497	219.45	0.0031	6.7563	6.6609
	126->130	0.50971					
10	120->127	0.45028	5.7378	216.08	0.2842	-173.4853	-173.6476
	121->127	0.225					
11	118->127	-0.27449	5.8631	211.47	0.0196	1.9069	1.9004
	119->127	0.25085					
	124->127	0.51991					
12	118->127	-0.34796	5.9179	209.51	0.0016	1.2221	1.2189
	126->129	0.43688					
13	118->127	0.34367	5.9225	209.35	0.0120	-12.0052	-12.0704
	124->127	0.30043					
	126->129	0.35515					
14	123->127	0.49193	5.9298	209.09	0.0278	-11.7209	-11.6937
	125->127	-0.32882					
15	126->131	0.51752	6.0006	206.62	0.0057	5.086	5.1849
	126->134	-0.32723					
16	126->133	0.59445	6.1092	202.95	0.0034	-1.1017	-1.21
17	119->127	0.5296	6.1707	200.92	0.0815	1.6166	1.6241
18	126->134	0.45894	6.2551	198.21	0.0075	-2.4577	-2.5025
	126->137	0.29501					
19	117->127	-0.23273	6.4045	193.59	0.0019	11.3627	11.2209
	121->127	0.43826					
20	126->144	0.26095	6.4187	193.16	0.0445	-35.7523	-35.5744
21	117->127	0.27287	6.5004	190.73	0.0085	11.6368	11.6402
	118->127	-0.23044					
	120->127	-0.28014					

Num[a]	*Transition*[b]	*CI-coeff*[c]	*ΔE (eV)*[d]	*λ (nm)*[e]	*f*[f]	R_{vel}[g]	R_{len}[h]
	121->127	0.38391					
22	126->135	0.41502	6.5338	189.76	0.0380	-4.2376	-4.392
	126->136	-0.2336					
	126->137	0.28656					
23	122->128	0.58045	6.5479	189.35	0.0012	-3.9533	-3.949
24	126->136	0.39939	6.6086	187.61	0.0050	-7.3577	-7.6014
25	126->138	0.49175	6.6519	186.39	0.0043	11.6061	11.6093
26	114->127	0.35071	6.6767	185.70	0.0069	-2.3174	-2.3872
	125->132	0.28358					
27	125->130	0.39332	6.7210	184.47	0.0239	-1.3862	-0.6829
	125->131	0.22675					
28	126->135	-0.30924	6.7341	184.11	0.0097	-10.3374	-10.3456
	126->137	0.31296					
	126->144	-0.24231					
29	114->127	0.23202	6.7937	182.50	0.0078	-11.4534	-11.5118
	116->127	0.27535					
	117->127	0.38828					
30	123->130	0.28299	6.8156	181.91	0.0384	12.202	11.3733
	125->136	-0.24781					
31	126->139	0.34865	6.8649	180.61	0.0107	-9.6658	-9.5366
	126->140	-0.25203					
32	126->141	0.39155	6.9328	178.84	0.0106	-2.4158	-2.4711
	126->142	-0.22371					
33	115->127	0.38222	6.9785	177.67	0.0031	-5.9361	-5.8992
	122->132	-0.24951					
34	124->130	-0.26347	7.0051	176.99	0.0076	-20.0652	-19.4726
	124->131	0.3001					
	124->134	0.30562					
35	116->127	0.42809	7.0104	176.86	0.0022	3.2827	3.447
36	125->128	0.54289	7.0371	176.19	0.0064	18.3801	18.4615

[a]Number of the excited states; [b]Only transitions with contribution over 10.0% were listed; [c]Configuration-interaction coefficient; [d]Excitation energy; [e]Wavelength; [f]Oscillator strength; [g]Rotatory strength in velocity form (10^{-40} cgs); [h]Rotatory strength in length form (10^{-40} cgs).
